# Broad and Potent Neutralizing Antibodies Recognize the Silent Face of the HIV Envelope

**DOI:** 10.1016/j.immuni.2019.04.014

**Published:** 2019-06-18

**Authors:** Till Schoofs, Christopher O. Barnes, Nina Suh-Toma, Jovana Golijanin, Philipp Schommers, Henning Gruell, Anthony P. West, Franziska Bach, Yu Erica Lee, Lilian Nogueira, Ivelin S. Georgiev, Robert T. Bailer, Julie Czartoski, John R. Mascola, Michael S. Seaman, M. Juliana McElrath, Nicole A. Doria-Rose, Florian Klein, Michel C. Nussenzweig, Pamela J. Bjorkman

**Affiliations:** 1 Laboratory of Molecular Immunology, The Rockefeller University, New York, NY 10065, USA; 2 Laboratory of Experimental Immunology, Institute of Virology, Faculty of Medicine and University Hospital of Cologne, University of Cologne, 50931 Cologne, Germany; 3 German Center for Infection Research, partner site Bonn-Cologne, 50931 Cologne, Germany; 4 Division of Biology and Biological Engineering, California Institute of Technology, Pasadena, CA 91125, USA; 5 Westridge High School, 324 Madeline Drive, Pasadena, CA 91105, USA; 6 Vanderbilt Vaccine Center, Department of Pathology, Microbiology and Immunology, Vanderbilt University Medical Center, Nashville, TN 37232, USA; 7 Department of Electrical Engineering and Computer Science, Vanderbilt University, Nashville, TN 37232, USA; 8 Vaccine Research Center, National Institute of Allergy and Infectious Diseases, NIH, Bethesda, MD 20892, USA; 9 Vaccine and Infectious Disease Division, Fred Hutchinson Cancer Research Center, Seattle, WA 98109, USA; 10 Center for Virology and Vaccine Research, Beth Israel Deaconess Medical Center, Harvard Medical School, Boston, MA 02215, USA; 11 Center for Molecular Medicine Cologne (CMMC), University of Cologne, 50931 Cologne, Germany; 12 Howard Hughes Medical Institute, The Rockefeller University, New York, NY 10065, USA

**Keywords:** HIV-1, broadly neutralizing antibody, HIV-1 vaccine, immunotherapy, HIV-1 Env silent face, cryo-EM, humanized mice, glycan recognition, Env trimer

## Abstract

Broadly neutralizing antibodies (bNAbs) against HIV-1 envelope (Env) inform vaccine design and are potential therapeutic agents. We identified SF12 and related bNAbs with up to 62% neutralization breadth from an HIV-infected donor. SF12 recognized a glycan-dominated epitope on Env’s silent face and was potent against clade AE viruses, which are poorly covered by V3-glycan bNAbs. A 3.3Å cryo-EM structure of a SF12-Env trimer complex showed additional contacts to Env protein residues by SF12 compared with VRC-PG05, the only other known donor-derived silentface antibody, explaining SF12's increased neutralization breadth, potency, and resistance to Env mutation routes. Asymmetric binding of SF12 was associated with distinct N-glycan conformations across Env protomers, demonstrating intra-Env glycan heterogeneity. Administrating SF12 to HIV-1-infected humanized mice suppressed viremia and selected for viruses lacking the N448_gp120_ glycan. Effective bNAbs can therefore be raised against HIV-1 Env’s silent face, suggesting their potential for HIV-1 prevention, therapy, and vaccine development.

## Highlights

• Silentface (SF) bNAbs can reach substantial neutralization breadth and potency
• Defined structure of a SF bNAb bound to an Env trimer
• Binding of SF bNAb resulted in trimer asymmetry in the V3 region
• SF antibodies have *in vivo* activity and potential for clinical use

## Graphical Abstract

**Figure:**
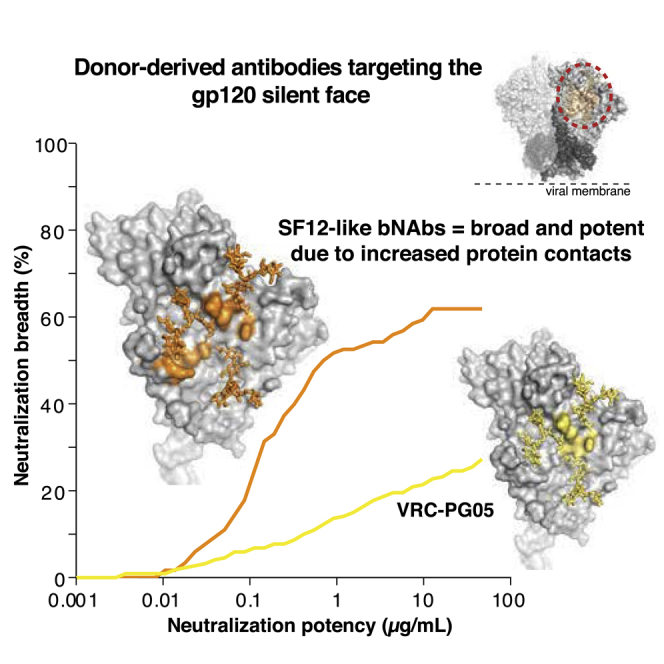

## Teaser

VRC-PG05 was the only donor-derived antibody against the silentface (SF) of HIV-1 envelope described to date. Schoofs et al. identify the antibody SF12 and its relatives, which recognize the center of the SF with a different angle and more extensive protein recognition than VRC-PG05, thereby achieving substantial neutralizing ability and potential for clinical use.

## Introduction

Neutralizing antibodies (NAbs) play a key role in antiviral immunity and are the correlate of protection of most available vaccines (Burton, 2002, Plotkin, 2010). The HIV-1 envelope glycoprotein (Env) is the only potential target for NAbs on the surface of the virus (Burton and Hangartner, 2016, Kwong and Mascola, 2018, Wibmer et al., 2015). Env is a trimeric spike composed of gp120/gp41 heterodimers that has evolved a plethora of immune escape mechanisms to evade antibody recognition. These include instability of the trimer, sparsity of spikes on the virion surface, high sequence divergence across strains, and epitope masking through its extensive glycan shield (Burton and Hangartner, 2016, Haynes, 2015, Klein and Bjorkman, 2010, Kwong and Mascola, 2018).

Consequently, effective humoral responses to HIV-1 typically only emerge several years after infection and only in a subset of HIV-1-infected individuals (Gray et al., 2011, Landais et al., 2016, Mikell et al., 2011, Rusert et al., 2016, Tomaras et al., 2011). Although ∼50% of chronically HIV-1-infected individuals develop some degree of cross-clade serum neutralization, only a small fraction of individuals mounts outstandingly broad and potent antibody responses against the virus (Doria-Rose et al., 2010, Hraber et al., 2014, Landais et al., 2016, Rusert et al., 2016, Sather et al., 2009, Simek et al., 2009). The development and use of single B cell antibody cloning revealed that this activity can usually be attributed to one or a combination of broadly neutralizing antibodies (bNAbs) that target HIV-1 Env (Scheid et al., 2009a, Scheid et al., 2009b, Scheid et al., 2011, Walker et al., 2009, Walker et al., 2010, Wu et al., 2010).

NAbs are proposed to interfere with viral entry in a variety of ways, including blocking receptor engagement, preventing membrane fusion, and enhancing decay of Env spikes (Burton and Hangartner, 2016). Pre-clinical and recent human studies have highlighted the potential of bNAbs for HIV-1 therapy and prevention (Bar et al., 2016, Bar-On et al., 2018, Barouch et al., 2013, Caskey et al., 2015, Caskey et al., 2017, Gautam et al., 2016, Julg et al., 2017, Lynch et al., 2015, Mendoza et al., 2018, Scheid et al., 2016, Shingai et al., 2013). Moreover, structural insights into mechanisms of bNAb binding have been key to designing novel immunogens and strategies for vaccination (Escolano et al., 2017, Jardine et al., 2013, Kwong and Mascola, 2018, McGuire et al., 2013, Sanders et al., 2013, Ward and Wilson, 2017). However, there remain a number of challenges to the elicitation and clinical use of bNAbs. For vaccine efforts, these include unusual structural features of bNAbs such as large insertions/deletions, and/or unusual complementarity determining region (CDR) lengths as well as extensive somatic hypermutation (SHM), all of which are rare features in the human repertoire (Burton and Hangartner, 2016, Haynes and Burton, 2017, Sok and Burton, 2018). For clinical use of bNAbs, viral coverage gaps, manufacturability, and pre-existing bNAb resistance represent potential problems (Escolano et al., 2017, Gruell and Klein, 2018, Sok and Burton, 2018). Thus, there is a continuing need to identify bNAbs that may be more readily elicited by vaccination and that are suitable for clinical use.

Although many bNAbs have been characterized, their targets, or “sites of vulnerability”, on the HIV-1 Env spike appear to be limited (Burton and Hangartner, 2016, Kwong and Mascola, 2018, Sok and Burton, 2018, Ward and Wilson, 2017, West et al., 2014, Wibmer et al., 2015). Numerous monoclonal antibodies recognize the CD4-binding site, the V3-glycan patch, the V2-apex, the membrane proximal external region (MPER), and several epitopes encompassing the gp120-gp41 interface (Burton and Hangartner, 2016, Kwong and Mascola, 2018, Sok and Burton, 2018, Ward and Wilson, 2017, Wibmer et al., 2015). In contrast, VRC-PG05 is the only donor-derived antibody isolated to date that binds to the highly glycosylated “silent face” of gp120 (Zhou et al., 2018). However, VRC-PG05 neutralized only 27% of tested HIV-1 strains and had a relatively high mean IC_50_ of 0.8 μg/mL, leaving uncertain the potential usefulness of this epitope for vaccine design, therapy, or prevention.

Here, we describe silent face (SF) bNAbs targeting a VRC-PG05-related epitope that cover up to 62% of evaluated strains with a mean IC_50_ of 0.20 μg/mL. To characterize the binding mechanism of the new antibodies, we determined the 3.1 Å crystal of the unbound SF12 Fab and a 3.3 Å cryo-EM structure SF12 Fab bound to the clade B B41 Env trimer. We found that SF12 binds the center of the Env silent face with a different orientation and set of contacts than VRC-PG05. The overall breadth and potency achieved by SF12 suggests that the silent face is an additional target for vaccine design and that antibodies to this site may be clinically useful as a complement to other available bNAbs.

## Results

### Isolation of an Antibody Family from Donor 27845 by B Cell Culture and BG505 Sorting

**Figure 1:**
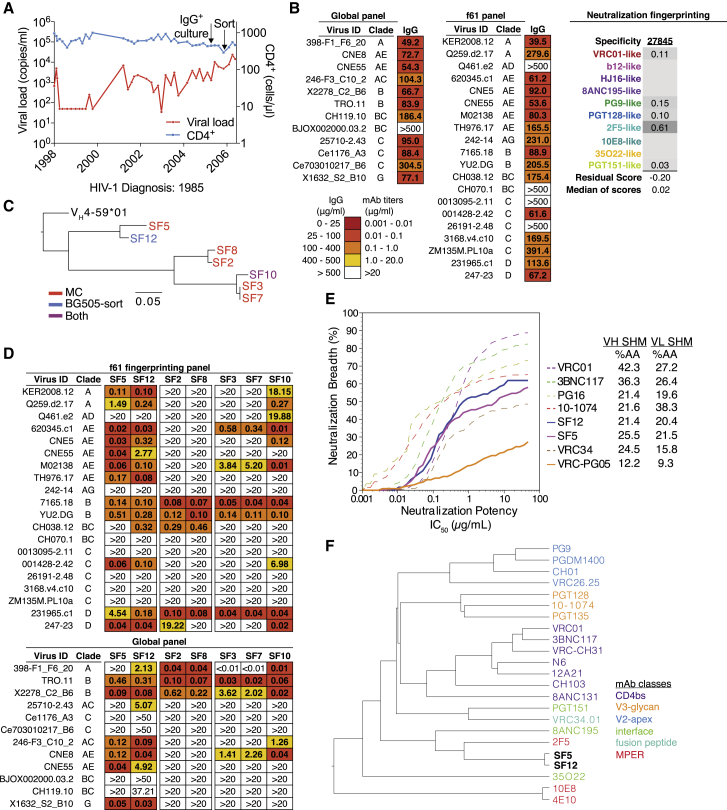
Isolation of Antibody Family from Donor 27845 by B Cell Culture and BG505 Sorting (A) Viral load and CD4^+^ T cell counts of HIV-1-infected subject 27845 over time. Arrows indicate time points of B cell microculture and BG505.SOSIP.664 bait-sorting. (B) Neutralization data of donor 27845’s serum IgG in 2005 against a 12-virus cross-clade panel (global) and a 20-virus fingerprinting panel (f61). Shown are median inhibitory concentrations (IC_50_) in μg/mL. On the right, fingerprinting analysis of f61 serum neutralization. Neutralization testing performed in duplicates, average shown. (C) Maximum-likelihood phylogenetic tree of heavy chain sequences of newly isolated antibody family. MC = Antibodies isolated by B cell microculture, BG505-sort = antibodies isolated by bait-sorting, Both = antibody found both by microculture and bait-sorting. (D) Neutralization of isolated antibody family members (IC_50_) against global and f61 virus panels. Legend as in (B). Neutralization testing performed in duplicates, average shown. (E) Neutralization coverage and potency of SF5 and SF12 on a 119-virus cross clade panel. Neutralization testing performed in duplicates, average shown. (F) Neutralization fingerprinting of SF5 and SF12 in comparison to other known anti-HIV-1 bNAbs. See also Figure S1 and Tables S1 and S2.

Donor 27845 was diagnosed with HIV-1 in 1985 and followed in a cohort of long-term non-progressors at the Fred Hutchinson Cancer Research Center from 1998–2006. Apart from an interventional study during which the subject started and stopped anti-retroviral therapy (ART) at set intervals from 1998–2001, the subject has been off ART (Figure 1A). The individual’s purified immunoglobulin G (IgG) isolated from a 2005 time point was evaluated for neutralization against a 12-virus panel representative of the global epidemic (deCamp et al., 2014) (Figure 1B) and found to be both broad and potent with a coverage of 92% and an average median inhibitory concentration (IC_50_) of 92.3 μg/mL (Figure 1B). To inform potential antibody isolation strategies, neutralization fingerprinting of the subject’s IgG was performed, but the results were inconclusive due to borderline prediction confidence scores (Doria-Rose et al., 2017) (Figure 1B).

Based on the fingerprinting results, we employed an unbiased B cell microculture approach for antibody cloning (Doria-Rose et al., 2015, Huang et al., 2013). From a starting number of 4.4 × 10^4^ memory B cells, we identified seven B cells, six of which were members of a single clone, that showed potent anti-HIV-1 neutralizing activity against two indicator strains. Subsequent single B cell sorting using fluorescently labeled BG505.SOSIP.664 native-like Env trimers (Sanders et al., 2013) yielded two additional members of this antibody family, one of which was identical to an antibody obtained in the B cell culture. The members of the clone utilized V_H_4-59^∗^01 and V_K_3-20^∗^01 heavy and light chain variable gene segments and included CDRH3s and CDRL3s of 23 and 6 amino acids, respectively (Table S1). V_H_ gene segment mutation frequencies ranged from 17%–25% of nucleotides (21%–39% amino acids), and V_K_ gene segment mutation frequencies ranged from 15%–21% of nucleotides (20%–29% amino acids), intermediate rates of SHM for HIV-1 bNAbs (Table S1). Based on heavy chain sequences, the family segregated into three phylogenetic branches (Figure 1C), with the SF5/SF12 branch showing a three-nucleotide CDRH2 insertion.

When tested on two representative panels of 20 (f61 panel) and 12 (global panel) viruses (deCamp et al., 2014, Doria-Rose et al., 2017), members of the V_H_4-59 clone showed diverse levels of activity and breadth (Figure 1D). Two closely related members of the V_H_4-59 clone that were the most active, SF5 and SF12, were then evaluated against a 119-virus panel representative of all major circulating HIV-1 clades (Figure 1E; Table S2) (Freund et al., 2017, Mouquet et al., 2012). SF5 and SF12 neutralized 58% and 62% of viruses in this larger panel, with geometric mean IC_50s_ of 0.25 and 0.20 μg/mL, respectively. Notably, SF12 neutralized all of 18 tested clade AE viruses across the three panels and showed a pattern of neutralizing activity that differed from previously described bNAbs (Figure 1F). Overall, the antibody clone recapitulated the majority of the polyclonal IgG neutralization activity, with the potency correlation between isolated monoclonal antibodies (mAbs) and donor 27845’s IgG resembling those of other elite neutralizers from whom we previously isolated bNAbs (Freund et al., 2017, Scheid et al., 2011).

We next evaluated potential autoreactivity and polyreactivity of SF12 and SF5 using HEp-2 staining (Haynes et al., 2005) and a baculovirus-based polyreactivity assay (Hötzel et al., 2012), respectively (Figure S1). In contrast to bNAbs with known autoreactive and polyreactive properties such as 2F5 and 4E10 (Haynes et al., 2005), we found minimal to no autoreactivity or polyreactivity for SF5 and SF12 (Figures S1A and S1B). In addition, the pharmacokinetics of SF12 in mice were similar to those of 3BNC117, a bNAb that exhibits a typical IgG1 half-life in macaques (Gautam et al., 2016) and humans (Caskey et al., 2015) (Figure S1C). We conclude that the SF antibody family achieves substantial anti-HIV-1 neutralization with an intermediate degree of somatic hypermutation and no evidence for autoreactivity.

### Antibodies SF5 and SF12 Bind a Distinct Epitope on the gp120 Portion of Env

**Figure 2:**
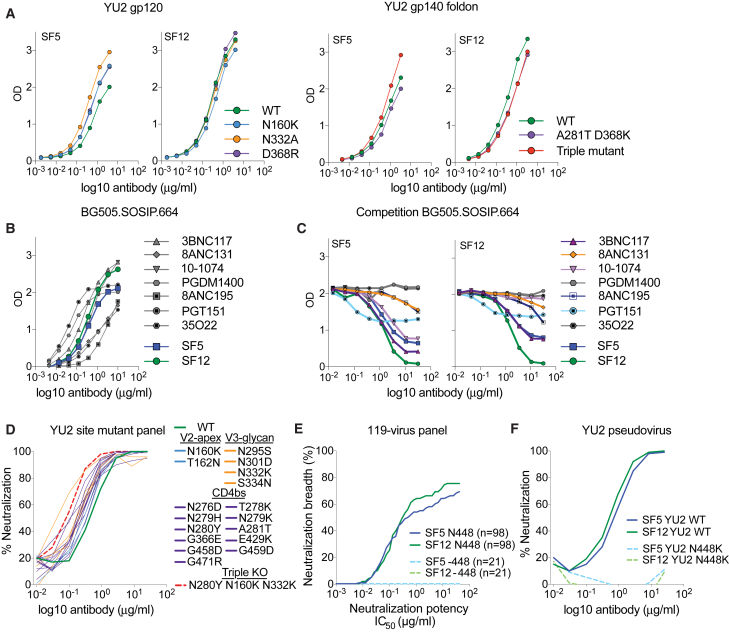
Antibodies SF5 and SF12 Bind a Distinct Epitope on the gp120 Portion of Env (A) ELISA of SF5 and SF12 against a gp120 monomer and a gp140 foldon trimer derived from HIV-1 strain YU2. Wild-type proteins and various site mutants of the proteins in common bNAb epitopes (CD4-binding site, V3-glycan, Apex) were tested. Triple mutant = N160K, A281T + D368K, N332K. Data representative of 3 repeat assays. (B) ELISA of SF5 and SF12 as well as reference bNAbs targeting 6 known epitopes against the BG505.SOSIP.664 trimer. Data representative of 3 repeat assays. (C) Competition ELISA with reference bNAbs targeting 6 known epitopes to evaluate interference with SF5 and SF12 binding to the BG505.SOSIP.664 trimer. Competing antibodies were added in a dilution series starting at 32 μg/mL. SF5 and SF12 were added at a constant concentration of 0.5 μg/mL. Data representative of 3 repeat assays. (D) Neutralization testing of SF12 against a panel of YU2 site mutants covering major epitopes on the HIV-1 spike. Neutralization testing performed in duplicates, average curves shown. (E) Computational analysis of 119-virus cross clade panel neutralization. (F) Neutralization testing of SF5 and SF12 against an HIV-1 pseudovirus based on strain YU2 carrying a mutation at the PNGS N448_gp120_. Testing done in duplicates, average shown.

To map the epitope recognized by SF5 and SF12, we performed ELISAs using HIV-1 Env proteins. Both antibodies bound to monomeric YU2 gp120, indicating that a portion of the epitope is contained within the gp120 subunit of Env (Figure 2A). We subsequently evaluated ELISA binding to site-directed mutants in monomeric YU2 gp120 and an uncleaved YU2 gp140 foldon trimer (Yang et al., 2000) that define common epitopes. Mutation of the CD4-binding site (D368R/D368K_gp120_, A281T_gp120_) (Dosenovic et al., 2015, Horwitz et al., 2013, Olshevsky et al., 1990), the V3 glycan patch (N332A/N332K_gp120_) (Horwitz et al., 2013, Mouquet et al., 2012), and the V2 apex epitope (N160K_gp120_) (Walker et al., 2009), alone or in combination, did not abrogate SF5 or SF12 binding (Figure 2A). SF5 and SF12 also bound to a cleaved soluble native-like BG505.SOSIP.664 trimer (Sanders et al., 2013) (Figure 2B). Taken together, these results suggested that SF5 and SF12 bind an epitope that is present on gp120 monomers and both cleaved and uncleaved Env trimers.

We performed competition ELISAs to assess binding to the BG505.SOSIP.664 trimer using antibodies targeting the CD4-binding site (3BNC117, 8ANC131), the V3 glycan patch (10-1074), the V2-apex (PGDM1400) and the gp120-gp41 interface (8ANC195, PGT151 and 35O22). Both SF5 and SF12 competed strongly with themselves and each other (Figure 2C). 3BNC117, a CD4-binding site antibody that bridges adjacent protomers within a trimer and has a broad contact surface with gp120 (Lee et al., 2017), showed competition with both SF5 and SF12. Incomplete competition was also observed for the CD4-binding site antibody 8ANC131 and for the gp120-gp41 interface antibodies 8ANC195 and PGT151. Moreover, the V3-glycan targeting antibody 10-1074 competed strongly with SF5 but not with SF12 (Figure 2C). We also assessed the neutralizing activity of SF12 on a YU2 pseudovirus mutant panel comprising a number of mutations that impair the activity of CD4-binding site, V3-glycan and V2-apex antibodies using a TZM.bl-based *in vitro* neutralization assay. SF12 neutralizing activity was insensitive to the mutations, including a triple mutant carrying mutations in all three epitopes (N280Y_gp120_, N160K_gp120_, N332K_gp120_) (Figure 2D). These data indicate that SF5 and SF12 bind a distinct epitope near the epitopes for CD4-binding site bNAbs and gp120-gp41 interface bNAbs 8ANC195 and PGT151.

Computational analysis (West et al., 2013) of available neutralization data suggested that SF5/SF12 depend on the presence of a glycan at N448_gp120_ (Figure 2E). To verify that the neutralizing activity of SF5 and SF12 depended on this potential N-linked glycosylation site (PNGS), we showed that these antibodies failed to neutralize a mutant HIV_YU2_ pseudovirus lacking the N448_gp120_ glycan (Figure 2F). The PNGS at position 448_gp120_ is at the center of one of the most highly glycosylated parts of the HIV-1 trimer, also known as the silent face (Wyatt et al., 1998). Although comparisons of synonymous versus non-synonymous mutations suggested that the silent face is under immunologic pressure (Stewart et al., 2001), antibodies that bind to the center of this region have been difficult to isolate. Indeed, VRC-PG05 represented an, until now, unique example of a host-derived bNAb that specifically targets the center of the silent face with a focus on the glycan site at N448_gp120_ (Zhou et al., 2018). The discovery and characterization of SF12 and related silent face bNAbs shows that this epitope can be targeted by antibodies with greater breadth and potency than VRC-PG05.

### Structure of the Natively Glycosylated SF12-Env Complex

**Figure 3:**
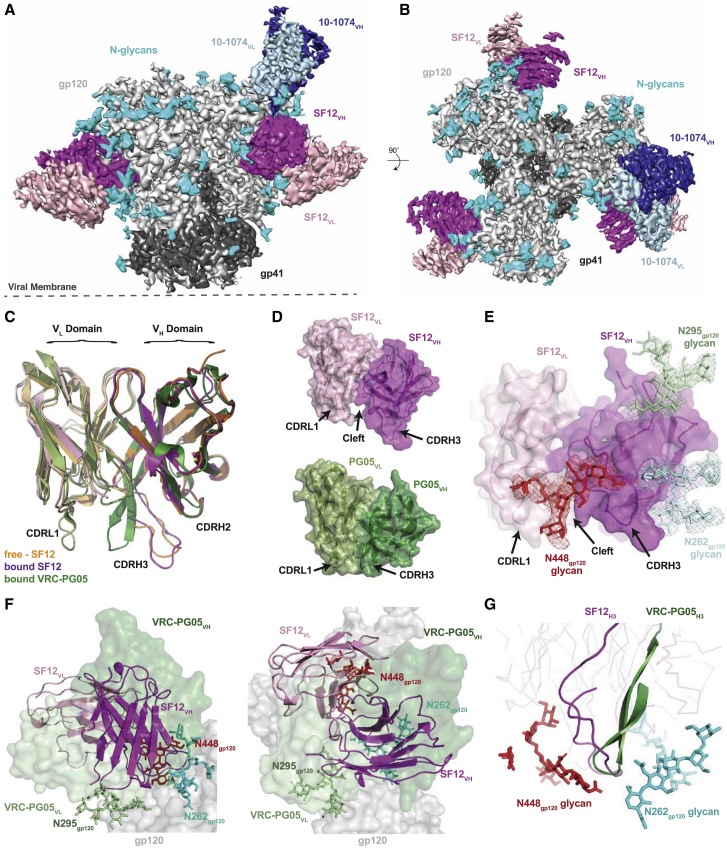
Structural Overview of the SF12-B41-10-1074 complex (A and B) Side-view (A) and top-view (B) of the final 3.3 Å single-particle cryo-EM reconstruction of the SF12-B41-10-1074 complex colored by components (dark gray, gp41; light gray, gp120; magenta, SF12 V_H_; pink, SF12 V_L_; blue, 10-1074 V_H_; light blue, 10-1074 V_L_; cyan, N-glycans). (C) Superposition of V_H_-V_L_ domains (235 Cα atoms) of unliganded SF12 (orange), Env-bound SF12 (magenta), and core gp120-bound VRC-PG05 (green) Fabs, showing differences in CDR conformations between SF12 and VRC-PG05. (D) Surface representation of SF12 (magenta/pink) and VRC-PG05 (green/pale green) Fabs illustrating differences in CDRL1 and CDRH3 loop conformations. (E) Surface representation of Env-bound SF12 Fab showing interactions with the N262_gp120_ (pale blue), N295_gp120_ (pale green) and N448_gp120_ (red) glycans at the SF12-Env interface. Cryo-EM density for individual glycans is shown contoured at 6σ. (F) Comparison of V_H_-V_L_ domain orientations of SF12 (magenta/pink; cartoon) and VRC-PG05 (green/pale green; surface). The V_H_-V_L_ domain orientation of SF12 on Env trimer is related by a 71° rotation and 0.5 Å translation to the VRC-PG05 variable domains after alignment against gp120 (gray; surface). (G) Overlay of CDRH3 loops of SF12 (magenta) and VRC-PG05 (green) after alignment of bound gp120s illustrates CDRH3-mediated recognition of the N448_gp120_ glycan (red; sticks) by both antibodies. See also Figures S2, S3, and S4 and Tables S3 and S4.

We determined a 3.1 Å crystal structure of the SF12 Fab and a 3.3 Å cryo-EM structure of a natively glycosylated clade B B41 SOSIP.664 trimer in complex with the SF12 Fab and a Fab from the V3/glycan patch bNAb 10-1074 (Figures 3A and 3B). Although 10-1074 Fab normally binds with a 3:1 Fab:Env trimer stoichiometry (Gristick et al., 2016), EM class averages showed either three or two SF12 Fabs bound to the Env trimer and only one 10-1074 Fab (Figures S2 and S3). Like VRC-PG05, for which a crystal structure was solved in complex with a monomeric gp120 core (Zhou et al., 2018), the SF12-trimer complex reveals recognition of an epitope focused on the N262_gp120_, N295_gp120_, and N448_gp120_ glycans on the silent face of Env, rationalizing our binding and *in vitro* neutralization results (Figures 2A–2F). Superimposition of the free and Env-bound SF12 Fab structures showed only minor conformational changes resulting from Env glycan interactions with the SF12 Fab in the Env-bound structure, as evidenced by the 1.1 Å root-mean-square deviation (RMSD) relating 245 Cα atoms in the V_H_ and V_L_ domains of the free and bound Fabs (Figure 3C).

We found three distinct differences between the structures of a VRC-PG05 Fab-monomeric CNE55 gp120 core and the SF12-Env trimer complexes (Zhou et al., 2018). First, the longer CDRH3 of SF12 was extended in a different conformation from that of the shorter CDRH3 in VRC-PG05, resulting in a RMSD of 3.1 Å across 130 Cα atoms when superposing the V_H_ domains from the Fab-bound structures (Figure 3C). Second, the SF12 CDRL1 and CDRL3 loops adopted conformations different from those of their VRC-PG05 counterparts (Figure S4). In the SF12 Fab, the CDRH3 and CDRL loops form a groove at the Fab-antigen interface that accommodates the N448_gp120_ glycan, which contrasts with the wedge between the VRC-PG05 CDRH3 and CDRL1 loops that penetrates through Env glycans (Figures 3D and 3E). Third, the orientation of the SF12 Fab differed from that of VRC-PG05 Fab, with the SF12 Fab exhibiting an almost perpendicular binding angle to the silent face epitope compared with the VRC-PG05 orientation (Figures 3F and S4). To evaluate this difference, we calculated the rotation and translation of the V_H_-V_L_ domains of the Fab portions of the SF12-Env trimer and VRC-PG05-gp120 complex structures, finding that the orientations of the SF12 and VRC-PG05 V_H_-V_L_ domains differed, with the axis of the SF12 Fab at a steeper angle (by ∼71°) to the silent face epitope than the axis of VRC-PG05. Despite differences in approach angles to the silent face epitope, SF12 and VRC-PG05 shared a common mode of interaction with the N448_gp120_ glycan, mediated in each case by their CDRH3 loops (Figure 3G). We conclude that SF12 binds a VRC-PG05-related epitope with a different angle of approach and an altered mode of recognition from VRC-PG05.

### SF12 Recognizes a Mostly Glycan-Focused Epitope on HIV-1 Env

**Figure 4:**
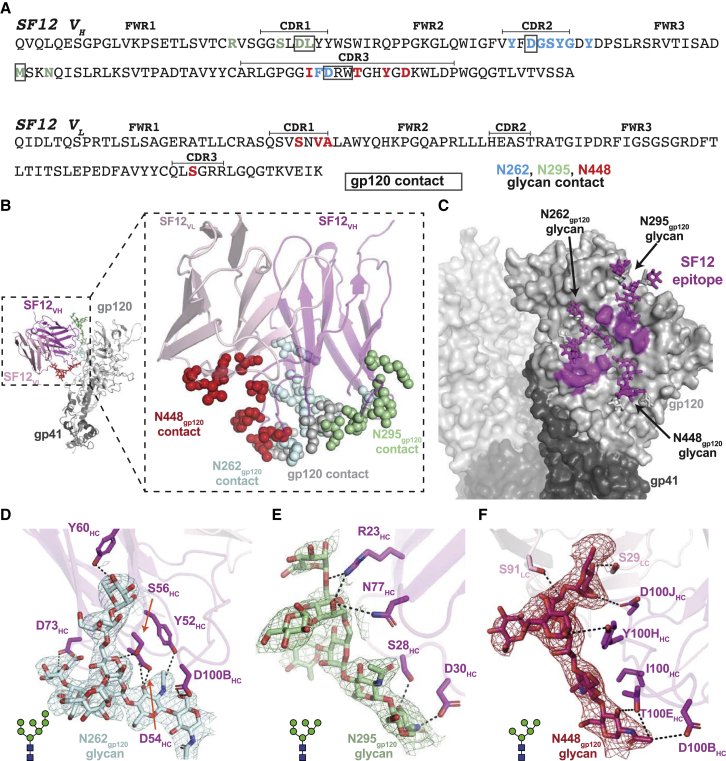
Details of SF12 Epitope and Glycan Recognition (A) Sequence of SF12 variable domains with antibody regions annotated using IMGT sequence analysis (CDR loops are bracketed). SF12 residues that contact N-linked glycans are in blue (N262_gp120_), green (N295_gp120_), and red (N448_gp120_), while gp120-contacting residues are boxed. Contacting residues in the SF12 paratope and epitope were defined as two residues containing any atom within 4 Å of each other. (B) Structure of a SF12-B41 gp120 protomer from the trimer complex, showing paratope residues as spheres (inset). Color scheme is the same as in (A). (C) Surface representation of B41 trimer, with SF12 epitope highlighted in magenta. (D–F) Stick representation of residue level contacts for N262_gp120_ (D), N295_gp120_ (E), and N448_gp120_ (F) glycans. Potential hydrogen bonds are shown as black dashes. Cryo-EM density maps contoured at 6σ are shown for individual glycans. See also Figure S4 and Table S5.

In contrast to the non-natively glycosylated monomeric CNE55 gp120 core that was complexed with VRC-PG05 Fab (Zhou et al., 2018), the relatively high resolution cryo-EM structure of the natively glycosylated SF12-Env trimer complex allowed modeling of N-linked glycans in the B41 Env trimer (Figure 3). Given the asymmetric Fab binding in our complex, we characterized the SF12 epitope and paratope using a gp140 protomer in which the SF12, but not the 10-1074, Fab was bound (Figures 4A–4C). Consistent with differing binding angles and CDR loop conformations, SF12’s footprint on Env differed from that of VRC-PG05, such that interactions with both N-linked glycan and peptide components mapped almost exclusively to the SF12 heavy chain (Figures 4A–4C). For example, ∼7% of the buried epitope surface resulted from interactions with SF12’s light chain (∼1,843 Å^2^ buried surface area (BSA) of epitope against SF12 heavy chain versus ∼135 Å^2^ against the SF12 light chain; Table S5), compared with ∼36% of buried epitope surface at the VRC-PG05 light chain interface. This difference is likely due to the longer CDRL1 and L3 loops on VRC-PG05, which penetrate the glycan-rich epitope (Figures 3C and 3D).

In the Env protomer used for epitope analysis, we interpreted densities for an ordered GlcNAc_2_Man_7_ at N262 gp_120_, a GlcNAc_2_Man_6_ at N295_gp120_, and a GlcNAc_2_Man_5_ at N448_gp120_ (Figures 4D–4F). Similar to VRC-PG05, the N262_gp120_, N295_gp120_, and N448_gp120_ glycans constituted ∼75% of the epitope surface (Table S5), although comparisons must be interpreted cautiously due to (1) the use of a high mannose-only monomeric gp120 core for the VRC-PG05 complex structure (Zhou et al., 2018) versus a natively glycosylated native-like Env trimer for the SF12 complex structure, and (2) the higher resolution (2.4 Å) of the VRC-PG05-gp120 crystal structure than that of the SF12-Env trimer cryo-EM structure (3.3 Å). Mapping key residues involved in SF12-Env interactions identified determinants of glycan recognition mediated by specific regions in the SF12 paratope. For example, SF12’s CDRH1 and framework region 3 (FWR3) interacted exclusively with the N295_gp120_ glycan, while CDRH2 solely engaged the N262_gp120_ glycan (Figures 4D and 4E). SF12 interactions with the N448_gp120_ glycan were mediated mainly by CDRH3, with additional contacts observed with the light chain CDRL1 and CDRL3 loops (Figure 4F).

Because the frequency of SHM in SF12 is lower than typical for many HIV-1 bNAbs (Figure 1E; Table S1), we analyzed the contributions of mutated amino acid residues in the SF12 paratope to epitope recognition. Of the 17 V gene segment-encoded residues that contact the epitope, 9 arose through SHM, including an insertion in CDRH2 (Figure S5A). Consistent with glycans comprising most of the SF12 epitope, SHMs were mostly observed for residues at the antibody-glycan interface. However, unlike VRC-PG05, where SHMs mainly resulted in the removal of bulky tyrosine residues to accommodate glycans (Zhou et al., 2018), SF12 utilized tyrosines, as well as bulky hydrophilic residues, to facilitate interactions with the glycopeptide epitope (Figure S5). This demonstrates that SHMs adding bulky residues to the paratopes of antibodies against the glycan-rich silent face of HIV-1 Env are not necessarily an impediment to broad and potent neutralization by these antibodies (Figures 4D–4F and S5A).

**Figure 5:**
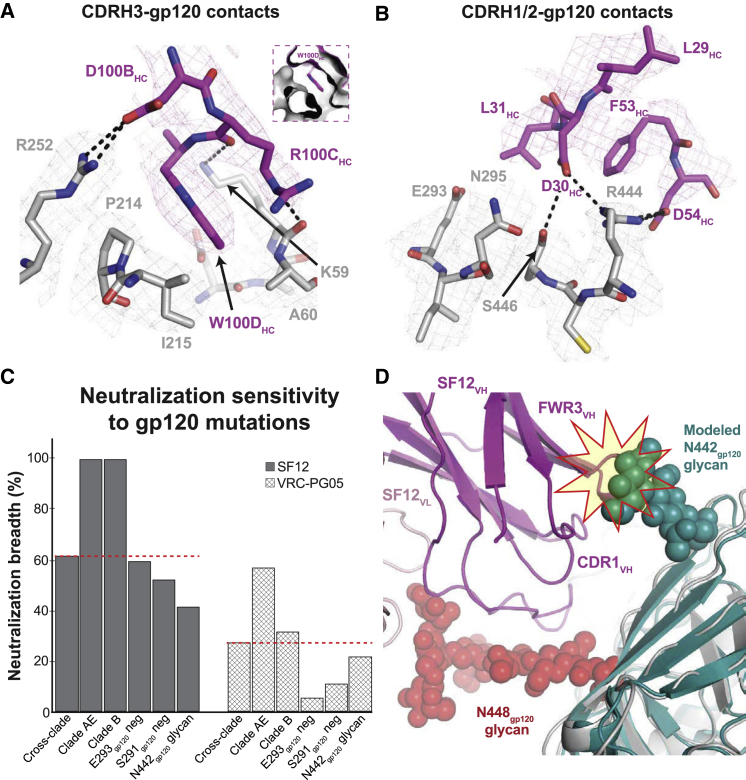
SF12 Engages Two Distinct Regions of gp120 Peptide Epitope (A) Stick representation of SF12 CDRH3 (magenta) and gp120 (gray) contacts at the SF12-Env interface. Trp100D_HC_ inserts into a hydrophobic pocket (inset) stabilized by potential hydrogen bond interactions (black dashes) with neighboring residues. (B) Stick representation of SF12 CDRH1 and H2 residues (magenta) contacting gp120 residues (gray). Potential hydrogen bonds are shown as black dashes. Density maps for SF12 and gp120 residues are shown as magenta and gray meshes, respectively, contoured at 8σ. (C) Comparison of SF12 and VRC-PG05 neutralization breadth for different viral characteristics. The red dashed line indicates neutralization breadth for SF12 (62%) and VRC-PG05 (27%) against a cross-clade panel. (D) Modeling of the N442_gp120_ glycan from clade C 426c SOSIP trimer (teal; PDB: 6MYY) was achieved by aligning gp120 coordinates from the two structures. Potential clashes with SF12 heavy chain (magenta) regions are highlighted. See also Figure S5.

The protein component of the SF12 epitope (∼25% of the epitope surface) mapped to two regions of gp120 (Figures 5 and S4). The first region involved residues from the gp120 β4 and β7 strands and the N terminus of gp120 that were engaged by regions of CDRH3 that penetrated the glycan shield (Figure 5A). In this interaction, SF12 CDRH3 residues R100C_HC_ and D100B_HC_ formed potential hydrogen bonds with Env residues K59_gp120_ and R252_gp120_, respectively (Figure 5A). These interactions contributed to the formation of a hydrophobic pocket on gp120 into which SF12 residue Trp100D_HC_ inserted, shielding this exposed hydrophobic residue at the tip of the CDRH3 loop (Figure 5A, inset). The second protein component of the SF12 epitope resembled part of the VRC-PG05 gp120 protein epitope, involving residues from gp120 β12 and β22 that interacted with the SF12 CDRH1 and H2 loops (Figure 5B). In this region, SF12 utilized aspartates at positions 30_HC_ and 54_HC_ to mediate contacts with gp120 residues N295_gp120_, R444_gp120_, and S446_gp120_ (Figure 5B). Unlike VRC-PG05, SF12 directly engaged the protein component of the epitope, forming extensive hydrogen bonds with surrounding residues. The increased epitope surface area contributed by gp120 peptide components (∼25% for SF12 versus ∼12% for VRC-PG05) likely contributes to the observed differences in neutralization potency and breadth for the two antibodies (Figure 1E). Overall, our structure of SF12 bound to a natively glycosylated Env trimer allows detailed insights into SF12 Env-glycan interactions and demonstrates that SF12 forms more extensive protein contacts with Env than VRC-PG05.

### Resistance to SF12 Is Driven by Glycan Contacts

**Figure 6:**
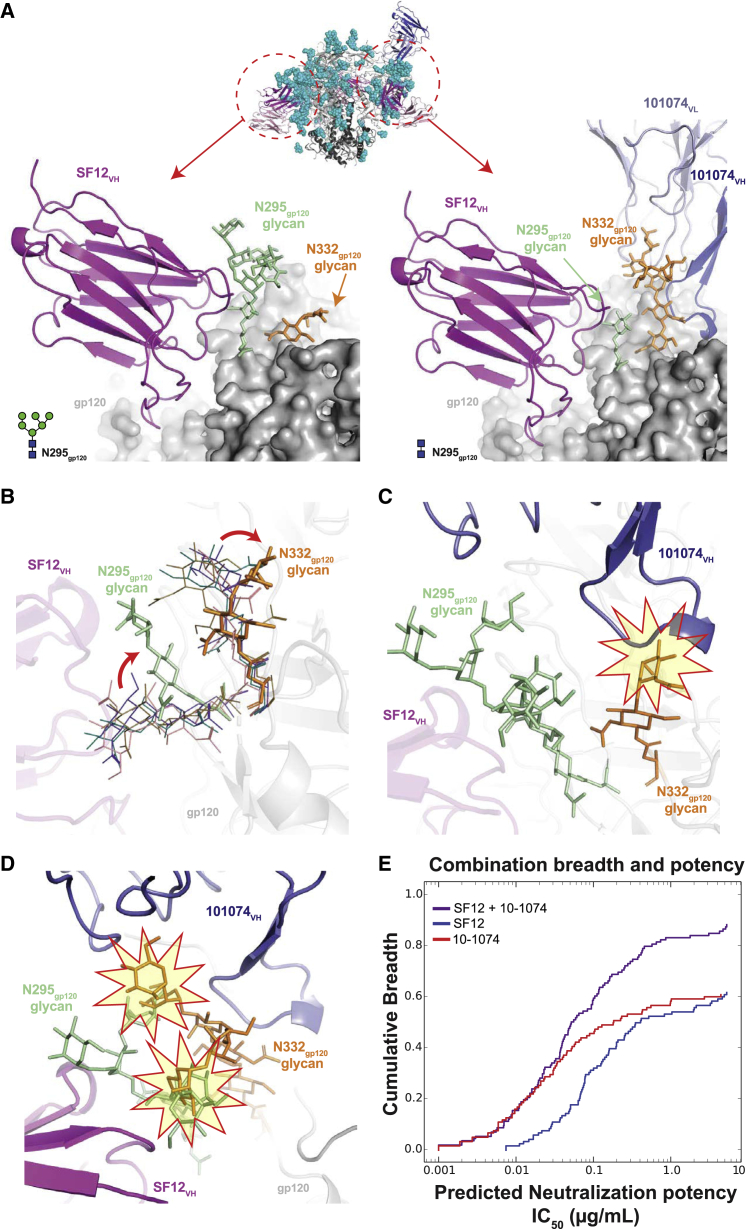
SF12-B41-10-1074 Structural Asymmetry Is Explained by N295_gp120_ Glycan Heterogeneity (A) Comparison of cryo-EM density for N295_gp120_ (green) and N332_gp120_ (orange) glycans across protomers within the SF12-B41-10-1074 trimer complex. In each protomer, SF12 (magenta) was bound, but 10-1074 (blue) binding was only observed when the N295_gp120_ glycan was modeled as GlcNAc_2_ (right panel). (B) Overlay of N295_gp120_ and N332_gp120_ glycans after aligning gp120 protomers from cryo-EM structures of SF12-B41-10-1974, PDB: 6CUE, PDB: 6DCQ, PDB: 5V8M, and PDB: 6CRQ. Positions for the N295_gp120_ and N332_gp120_ glycans in the SF12-bound Env (stick representation) and all other models (line representation) are shown. SF12-induced conformational changes are indicated by the red arrow. (C) Modeling of the 10-1074 Fab (blue cartoon) onto the SF12-gp120 protomer (A: left panel). Potential clashes between 10-1074 CDRH3 and the N332_gp120_ glycan are highlighted. (D) Alignment of gp120 portions of the SF12-bound (A: left panel) and SF12 plus 10-1074-bound (A: right panel) protomers. Potential clashes involving the N295_gp120_ and N332_gp120_ glycans (highlighted stars) when both glycans are processed beyond a core pentasaccharide are shown. (E) Predictive neutralization profiles for combination therapy with SF12 and 10-1074 bNAbs at a 10 μg/mL concentration. See also Figure S5 and Tables S6 and S7.

To confirm our structural findings and assess possible mechanisms of resistance to SF12 and SF5 neutralization, we created a series of mutant BG505 and YU2 pseudoviruses and evaluated their sensitivity to neutralization in TZM.bl assays. Disruption of the PNGS at N448_gp120_ (N448K, N448S) or N262_gp120_ (N262S, N262W) abrogated SF5 and SF12 neutralization (Table S6). In contrast, removal of the glycan at 295_gp120_ slightly improved neutralization by both SF5 and SF12 for the BG505 and YU2 strains, indicating that these antibodies accommodate, rather than make favorable contacts with, the glycan at N295_gp120_. This result was consistent with neutralization data against strains lacking the N295_gp120_ glycan (Table S7) and the SF12-B41 complex structure, as one gp140 protomer in the trimer showed density for GlcNA_2_ at the N295_gp120_ glycan despite the presence of bound SF12 Fab (Figure 6A).

To explore protein-protein interactions at the SF12-Env interface, we examined mutations in positions 214_gp120_ (P214I, P214Q), 291_gp120_ (S291P, S291T), 293_gp120_ (Q/V293E, Q/V293K, Q/V293R), and 444_gp120_ (R444T) in the Envs of the BG505 and YU2 pseudoviruses. Unlike VRC-PG05, where a dominant means to escape antibody neutralization was achieved by mutating E293_gp120_ to disrupt a critical contact with VRC-PG05’s CDRL1 and CDRH3 loops (Zhou et al., 2018), analysis of SF12 neutralization potency against the mutant pseudoviruses showed that SF12 remained potent against pseudoviruses with E293_gp120_ substitutions (Figure 5C; Tables S6 and S7). A 2-fold decrease in sensitivity to SF12 was observed for the R444T mutation, likely due to disruption of a hydrogen-bonding network between Env residue R444_gp120_ and SF12 residues D30_HC_ and D54_HC_. Env mutations at positions 214_gp120_ and 293_gp120_ preferentially affected the SF5 clonal variant, which is less potent than SF12. Effects due to mutations at the 293_gp120_ residue in both BG505 and YU2 backbones are likely explained by the presence of R31_HC_ in the SF5 CDRH1, which would directly engage with residue E293_gp120_ (Figure S5B).

Interestingly, a threonine at position 444_gp120_ is a strong predictor of resistance to SF12/SF5 neutralization, consistent with increased activity against clade AE and B viruses, which show a <1% frequency for a threonine at this position (Figure 5C; Table S7). Computational analysis of viral strains containing residue T444_gp120_ (37% of 3260 Env sequences in Antibody Database) (West et al., 2013) showed that 78% of the T444_gp120_-containing sequences included an asparagine at position 442_gp120_ to create a 442_gp120_ PNGS. To determine whether a glycan at position N442_gp120_ would disrupt SF12 binding, we modeled coordinates for the N442_gp120_ glycan from the clade C 426c DS-SOSIP structure (Borst et al., 2018) after superposing the PDB 6MYY gp120 onto the gp120 of our Env trimer structure and adding in the N442_gp120_ glycan. In the conformation observed on the 426c Env structure, the N442_gp120_ glycan would clash with SF12 heavy chain CDRH1 and FWR3 components, sterically hindering access to its epitope (Figure 5D). This likely explains SF12/SF5’s decreased breadth and potency against clade C viruses, 85% of which encode T444_gp120_ (Table S7). However, binding of SF12 could shift the position of the glycan, as seen for the N295_gp120_ glycan (Figures 6A and 6B), since SF12 shows neutralizing activity against some N442_gp120_ glycan-containing viruses (Table S7). Our results indicate that resistance to SF12/SF5 is mediated mainly through mutation of N-glycan sites, rather than Env protein residues, as seen for VRC-PG05.

### Heterogeneity of Glycan N295 Explains SF12-Env Complex Asymmetry

Given that previous V3-targeting bNAb structures showed symmetric binding of three V3-glycan patch Fabs per trimer (Ward and Wilson, 2017), including a recent cryo-EM structure of a BG505 DS-SOSIP bound by three PGT122 Fabs (Dingens et al., 2018), we sought to understand the asymmetric binding of the V3-directed 10-1074 Fab in the SF12-B41-10-074 structure (Figures 3A and 3B), noting that potential Env trimer asymmetry could not have been detected in the VRC-PG05 Fab-CNE55 complex structure, which was solved using a monomeric gp120 core (Zhou et al., 2018). Because asymmetric binding of SF12 and 10-1074 Fabs to the B41 Env trimer prevented the use of symmetry restraints during reconstruction and model building, density for each glycan at a PNGS was interpreted independently across gp140 protomers. Interestingly, glycan heterogeneity was observed at the N295_gp120_ glycan, which correlated with the presence or absence of the 10-1074 Fab (Figure 6A).

Alignment of SF12-bound gp120 protomers in the absence and presence of 10-1074 Fab (Figure 6A, left and right panels, respectively), revealed a low (<0.2 Å) RMSD for 452 gp120 Cα residues, suggesting that the lack of 10-1074 binding was not due to alteration in the protein portion of the V3 epitope. However, the N332_gp120_ glycan exhibited an altered conformation in the SF12-gp140 protomers without a bound 10-1074 Fab. Indeed, superposition of gp120 coordinates from cryo-EM structures that included coordinates for the N295_gp120_ and N332_gp120_ glycans showed that SF12 binding induced conformational changes that resulted in the N332_gp12i0_ glycan occluding the underlying protein portion of the V3-glycan patch epitope (Figure 6B). This conformation for the N332_gp120_ glycan would prevent 10-1074 binding due to steric clashes (Figure 6C). Moreover, the N295_gp120_ glycan conformation in the SF12-bound Env protomer and the N332_gp120_ glycan conformation in the 10-1074-bound Env protomer would be incompatible if both glycans were present in fully processed forms (Figure 6D). Taken together, this explains why a 10-1074 Fab could be accommodated on one protomer in our trimer complex, because the N295_gp120_ glycan appeared to be under-processed in the 10-1074-bound protomer (GlcNAc_2_ versus GlcNAc_2_Man_6_ observed in the other protomers).

Moreover, Fab interactions with the N295_gp120_ glycan likely explains observed differences in SF12 and SF5 binding properties, as SF5, but not SF12, was strongly competed by 10-1074 by ELISA (Figure 2B). SF5 includes a tyrosine in heavy chain FWR3 (Y77_HC_), which arose through SHM to engage the core GlcNAc_2_ of the N295_gp120_ glycan (Figure S5C). It is possible that during accommodation of 10-1074 binding, movement of the N295_gp120_ glycan toward SF5 Fab would clash with Y77_HC_, compared to the less bulky N77_HC_ in SF12 (Figure S5D). Despite the possibility for incompatible modes of binding to the same Env protomer with fully processed N-linked glycans at 295 and 332, SF12 and 10-1074 delivered as combination therapy could potentially achieve 90% breadth, as their neutralization patterns complement each another (Figure 6D). In sum, our SF12-Env trimer structure revealed N-glycan heterogeneity across different protomers of the same trimer, which was associated with differential binding to bNAbs.

### Evaluation of SF12 in HIV-1-Infected Humanized Mice

**Figure 7:**
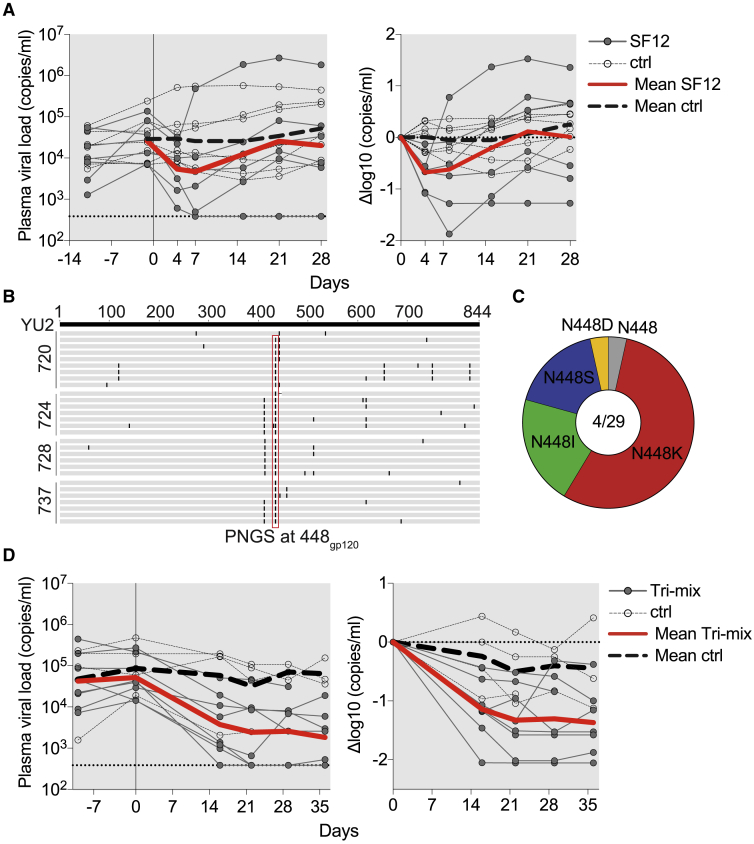
*In Vivo* Evaluation of SF12 IgG in HIV_YU2_-Infected Humanized Mice (A) SF12 monotherapy of humanized mice infected with HIV_YU2_. The left graph shows absolute viremia (y axis) in mice treated with SF12 (n = 7, dark gray, full circles) or untreated control mice (n = 7, empty circles) over the course of the experiment (x axis, days). Mice were infected 3 weeks prior to therapy initiation and received 1 mg of IgG as a loading dose followed by twice-weekly administration of 0.5 mg for 3 weeks. The dotted line at bottom indicates the limit of accuracy of the qPCR assay (384 copies/mL). The right graph shows relative log drop after initiation of SF12 therapy (Δlog10 copies/mL). Thick red lines and thick dashed gray lines indicate the mean viral load of treated and untreated mice, respectively. Data from one independent experiment. (B) Amino acid alignment of gp160 of wild-type YU2_gp160_ (top) with Env gp160 sequences obtained by single genome sequencing from plasma of SF12-treated mice 4 weeks posttherapy initiation. Each line represents one sequence; mouse identification numbers indicated on left. (C) Pie chart showing the amino acid distribution at position N448_gp120_ in mice that received SF12 at 4 weeks posttherapy initiation. Numbers inside pie chart correspond to number of mice sequenced/number of sequences obtained. (D) Antibody tri-mix (SF12, 10-1074, 3BNC117) therapy of HIV-1_YU2_-infected mice (n = 8). Mice (n = 4) with comparable viral loads and matched stem cell donors served as controls. Data from one independent experiment. Graphs as in (A).

We assessed the *in vivo* anti-HIV-1 activity of SF12 in humanized mice. Results from studies in mice are relevant to humans because clinical trials of HIV-1 bNAbs administered to infected patients showed that viral escape mutations are similar, if not identical, to those found in HIV-1-infected humanized mice (Bar-On et al., 2018, Caskey et al., 2015, Caskey et al., 2017, Horwitz et al., 2013, Klein et al., 2012, Mendoza et al., 2018, Scheid et al., 2016). Mice were infected with HIV-1_YU2_ (Zhang et al., 2002) (n = 7) and subcutaneously administered 1 mg of SF12 IgG followed by 0.5 mg of SF12 every 3 days for 3 weeks. Untreated HIV-1_YU2_-infected mice (n = 7) with comparable viral loads and matched stem cell donors served as controls. Mice treated with SF12 showed an average drop in viremia of 0.68 log_10_ copies/mL (range: 0.13–1.87 log_10_ copies/mL) with viral rebound occurring within 3 weeks after initiation of therapy (Figure 7A). To document escape mutations, single genome sequencing of gp160 was performed on mouse plasma samples 4 weeks after initiation of SF12 therapy. Of 29 independent isolates from 4 SF12-treated mice, we found mutations that abrogated the PNGS at position N448_gp120_ in 28 sequences (97%). These mutations exclusively affected position 448_gp120_ and mutated the N at position 448_gp120_ to K, S, D, or I through single nucleotide changes, with the mutation to K being most frequent (Figures 7B and 7C). We conclude that antibody SF12 can reduce viremia and exerts strong selective pressure on HIV-1 *in vivo*.

While antibody therapy with two effective bNAbs (PG16, NIH45-46^G54W^) failed to mediate long-term viral suppression (Klein et al., 2012), a triple antibody regimen comprising 3BNC117, 10-1074, and PG16 suppressed viremia in infected humanized mice for several weeks (Horwitz et al., 2013). To determine whether SF12 can contribute to control of viremia in humanized mice as part of a triple combination regimen, we treated HIV-1_YU2_-infected mice (n = 8) with a tri-mix of bNAbs SF12, 10-1074, and 3BNC117. Mice (n = 4) with comparable viral loads and matched stem cell donors served as controls. Mice receiving tri-mix therapy dropped 1.36 log_10_ copies/mL (range: 0.38–2.05 log_10_ copies/mL) during the 5 weeks of treatment. During the observation period, viremia was suppressed in all but one of the treated mice (Figure 7D). Thus, a triple antibody combination regimen that includes SF12 to restrict overall viral escape can mediate long-term control of HIV-1_YU2_ viremia *in vivo*.

## Discussion

Anti-HIV-1 bNAbs can control and prevent HIV-1 infection in humanized mice and macaques (Baba et al., 2000, Barouch et al., 2013, Hessell et al., 2009a, Hessell et al., 2009b, Klein et al., 2012, Klein et al., 2014, Mascola et al., 2000, Shingai et al., 2013, Shingai et al., 2014). In addition, bNAbs against two epitopes, the CD4-binding site and the V3-glycan patch, have been evaluated in humans: the antibodies were well-tolerated, exhibited typical IgG half-lives, and were effective in lowering viremia and preventing viral rebound in subjects undergoing analytical treatment interruption (Caskey et al., 2015, Caskey et al., 2017, Lu et al., 2016, Lynch et al., 2015, Mendoza et al., 2018, Scheid et al., 2016, Schoofs et al., 2016). Anti-viral effects of bNAbs in pre-clinical models and humans are mediated by a combination of virus neutralization and Fcγ receptor-mediated elimination of infected cells (Halper-Stromberg et al., 2014, Hessell et al., 2007, Lu et al., 2016), highlighting the potential of Fc effector functions as modulators of bNAb efficacy. Fc-modified bNAb variants with increased affinity for the neonatal Fc receptor showed half-lives of more than 70 days in humans (Gaudinski et al., 2018), suggesting that passive administration of anti-HIV-1 bNAbs may be a practical therapeutic strategy.

However, administration of individual bNAbs, like monotherapy with anti-retroviral drugs, leads to emergence of resistant viral variants. In contrast, combinations of bNAbs can control infection for prolonged periods of time in mice, macaques and humans (Bar-On et al., 2018, Barouch et al., 2013, Horwitz et al., 2013, Klein et al., 2012, Mendoza et al., 2018, Shingai et al., 2013). Similarly, a fully protective vaccine will likely require elicitation of bNAbs targeting multiple HIV-1 Env epitopes. This highlights a need to identify and clinically develop antibodies to new epitopes on the Env spike. Here, we studied the antibody response of a long-term non-progressor, identifying bNAb SF12 as part of a family of antibodies that target a glycan-focused epitope on the silent face of gp120. While the silent face of Env had not been considered a priority target for bNAbs or for vaccine development, the discovery of potent and broad antibodies such as SF12 suggests that targeting this site could contribute to therapies and vaccines.

The cryo-EM structure of SF12 bound to B41 Env trimer demonstrated that SF12 makes extensive contacts with N-glycans at positions 262_gp120_, 295_gp120_ and 448_gp120_. While our neutralization and structural data indicated that the PNGS at 295_gp120_ is dispensable for SF12 neutralization, we found the highly conserved glycans at N262_gp120_ and N448_gp120_ to be obligate contacts. Although SF12’s epitope is heavily glycan-focused, the antibody also contacts the protein backbone of Env, utilizing CDRHs 1 and 2 to mediate interactions at positions 293_gp120_, 444_gp120_ and 446_gp120_. In addition, SF12’s longer CDRH3, as well as its different angle of Env approach relative to VRC-PG05, allow SF12 to form additional protein contacts at positions 59_gp120_, 214_gp120_, and 252_gp120_, which likely explain the enhanced breadth and potency of SF12 compared to VRC-PG05, the only previously characterized antibody against the Env silent face (Zhou et al., 2018).

Consistent with previous observations suggesting that the N262_gp120_ glycan plays a crucial role in CD4-mediated viral entry (Moore et al., 1994) and that the N448_gp120_ glycan (>85% conserved) is less critical for viral infectivity (Behrens et al., 2016, Falkowska et al., 2014), our *in vivo* studies showed loss of N448_gp120_ as the major escape pathway upon SF12 treatment. Interestingly, the protein-protein contacts made by SF12 did not contribute to routes of viral escape, which contrasts with HIV-1’s dual mechanism of escape from VRC-PG05 by altering either the N448_gp120_ glycan or the glycan-proximal residue E293_gp120_. Indeed, viral evasion through alteration of the glycan shield or sequence diversity dominates most bNAb epitopes (Falkowska et al., 2014, Mouquet et al., 2012, Wagh et al., 2016, Walker et al., 2009). Thus, SF12 may represent one of the few glycopeptide recognizing bNAbs where sequence variability at the protein-protein interface does not induce viral resistance to SF12 neutralization.

Despite the highly conserved N262_gp120_ and N448_gp120_ glycans comprising its epitope, SF12 showed a gap in coverage that could not be explained by the removal of the N448_gp120_ glycan alone. Computational analysis of data from a 119-isolate viral panel suggested that glycosylation at position N442_gp120_ contributes to decreased SF12 breadth, likely through shielding of the N262_gp120_ and N448_gp120_ glycans, making it more difficult for SF12 to bind. SF12’s distinct epitope makes it potentially useful for therapy or prevention as part of a combination of bNAbs, particular in regions of the world with a high prevalence of AE or B HIV-1 clades (both clades lack the N442_gp120_ glycan). Indeed, the SF12 class of bNAbs is particularly potent and broad against clade AE viruses, which represent a coverage gap of the V3-glycan bNAbs, achieving 100% breadth and 10-fold increased potencies compared with VRC01. Furthermore, given its glycan-focused epitope, it may be possible to further improve SF12/SF5 potency, breadth, and resistance to escape using structure-based rational design as has been demonstrated for other anti-HIV-1 bNAbs (Diskin et al., 2011, Diskin et al., 2013, Rudicell et al., 2014, Xu et al., 2017).

Our data support the hypothesis that evolutionary pathways to silent face recognition are diverse. In addition to being isolated from two different donors, SF12 and VRC-PG05 arose from highly divergent V_H_ (V_H_4-59^∗^01 versus V_H_3-7^∗^01) and V_K_ genes (V_K_3-20^∗^01 versus V_K_4-1^∗^01) (Lefranc et al., 2009). Moreover, SF12 uses a long CDRH3 of 23 residues and a relatively short CDRL3 of 6 residues, while VRC-PG05 uses a moderately long 17-residue CDRH3 and an average 8-residue CDRL3. Sequence conservation between the CDR3s of the two antibodies is low with no discernible motifs in common, which contrasts other anti-HIV-1 bNAb families that show strong CDRH3 or CDRL3 length restrictions and/or recurring sequence motifs (McCoy and Burton, 2017). In addition, SF12 shows an intermediate rate of SHM compared with other HIV-1 bNAbs, with 21 and 19 amino acid substitutions in the heavy and light chains, respectively. This rate of mutation is comparable to or lower than rates found in V3-glycan patch antibodies, which are a heavily pursued vaccination target with a similar breadth of coverage (Escolano et al., 2017, McCoy and Burton, 2017). Taken together, these differences illustrate the divergence of solutions evolved by the SF12/SF5 and VRC-PG05 bNAbs to target the silent face epitope, whereby SF12’s maturation generated an antibody capable of accommodating Env sequence diversity at the protein surface.

In the context of eliciting silent face bNAbs, SF12 represents a promising path forward based on its breadth, potency, and relatively few SHMs. However, eliciting SF12-like bNAbs may face some of the same hurdles as other potential Env target sites, given its long CDRH3 and an insertion in CDRH2. In addition, the SF12-Env structure revealed that, despite targeting non-overlapping epitopes, SF12 binding induced a conformational change in the N295_gp120_ glycan that affected binding of the V3-glycan bNAb 10-1074. Thus, trimer-based immunogen design strategies, such as those employed to elicit V3-glycan targeting bNAbs (Escolano et al., 2017), should consider how alterations in the glycan shield around the silent face glycan patch may prevent maturation of SF12-like bNAbs.

Zhou et al. (2018) estimated VRC-PG05-like bNAbs are present in 10% of individuals in a 38-donor cohort. It is possible that SF antibodies were missed in previous studies, since the focus was on isolating antibodies to well-characterized epitopes. In particular, protein-based sorting strategies might not have allowed identification of these antibodies as also seen in this study, in which only two members of the clone were isolated by sorting using BG505 Env trimer as bait.

In summary, our results show that the glycan-rich silent face of HIV-1 Env can be targeted by bNAbs with potencies and breadths approaching those of antibodies against some of the more well-characterized epitopes. Overall, our findings extend current understanding of the recognition of glycan-focused epitopes on HIV-1 Env and expand the armamentarium of bNAbs available for HIV-1 therapy, prevention, and immunogen design.

## STAR★Methods

### Key Resources Table

**Table:** 

REAGENT or RESOURCE	SOURCE	IDENTIFIER
**Antibodies**		

Monoclonal anti-HIV-1 Env SF5	Michel C. Nussenzweig, The Rockefeller University (This Paper)	N/A
Monoclonal anti-HIV-1 Env SF12	Michel C. Nussenzweig, The Rockefeller University (This Paper)	N/A
Monoclonal anti-HIV-1 Env 3BNC117	NIH AIDS Reagent Program	Cat# 12474
Monoclonal anti-HIV-1 Env 10-1074	NIH AIDS Reagent Program	Cat# 12477
Monoclonal anti-HIV-1 Env PGDM1400	Dennis R. Burton, Scripps; Sok et al., 2014	N/A
Monoclonal anti-HIV-1 Env 8ANC131	Michel C. Nussenzweig, The Rockefeller University; Scheid et al., 2011	N/A
Monoclonal anti-HIV-1 Env 8ANC195	Michel C. Nussenzweig, The Rockefeller University; Scheid et al., 2011	N/A
Monoclonal anti-HIV-1 Env PGT151	Dennis R. Burton, Scripps; Falkowska et al., 2014	N/A
Monoclonal anti-HIV-1 Env 35O22	NIH AIDS Reagent Program	Cat# 12586
Monoclonal anti-human IgM-PE-Cy5, Clone G20-127	BD Biosciences	Cat# 551079; RRID: AB_394036
Monoclonal anti-human IgD-FITC, Clone IA6-2	BD Biosciences	Cat# 555778; RRID: AB_396113
Monoclonal anti-human CD3-APC-Cy7, Clone SK7	BD Biosciences	Cat# 557832; RRID: AB_396890
Monoclonal anti-human CD19-PE-Cy7, Clone HIB19	BD Biosciences	Cat# 560728; RRID: AB_1727438
Monoclonal anti-human CD16-PB, Clone N/A	Mario Roederer, NIH	N/A
Monoclonal anti-human CD19-BV421, Clone HIB19	Biolegend	Cat# 302233; RRID: AB_10897802
Monoclonal anti-human CD20-BV421, Clone 2H7	Biolegend	Cat# 302329; RRID: AB_10933088
Monoclonal anti-human CD3-PerCP-Cy5.5, Clone OKT3	Biolegend	Cat# 317336; RRID: AB_2561628
Monoclonal anti-human CD14-PerCP-Cy5.5, Clone HCD14	Biolegend	Cat# 325622; RRID: AB_893250
Monoclonal anti-human CD335-PerCP-Cy5.5, Clone 9E2	Biolegend	Cat# 331920; RRID: AB_2561665
Monoclonal anti-human CD66b-PerCP-Cy5.5, Clone G10F5	Biolegend	Cat# 305108; RRID: AB_2077855
Monoclonal anti-human anti-human-IgM-BV605, Clone MHM-88	Biolegend	Cat# 314523; RRID: AB_2562373
Monoclonal anti-human anti-human-IgG-APC, Clone G18-145	BD Biosciences	Cat# 550931; RRID: AB_398478
Anti-6X His tag antibody	Abcam	Cat# ab9108; RRID: AB_307016
Goat Anti-Human IgG Fc, Multi-Species SP ads-HRP	Southern Biotech	Cat# 2014-05; RRID: AB_2795580
Peroxidase AffiniPure Goat Anti-Human IgG, Fcγ fragment specific	Jackson ImmunoResearch	Cat# 109-035-098; RRID: AB_2337586

**Bacterial and Virus Strains**		

f61 Panel of 20 HIV-1 Env-pseudotyped viruses for neutralization fingerprinting	Nicole Doria-Rose, NIH; Doria-Rose et al., 2017	N/A
Global Panel of 12 HIV-1 Env-pseudotyped viruses	NIH AIDS Reagent Program; deCamp et al., 2014	Cat# 12670
119 HIV-1 Env-pseudotyped viruses cross-clade panel	Michael S. Seaman, BIDMC; Freund et al., 2017, Mouquet et al., 2012	N/A
YU2 HIV-1 Env-pseudotyped viruses carrying mutations in common anti-HIV-1-mAb binding sites	Florian Klein, University of Cologne	N/A
Replication-competent HIV_YU2_ (YU2-envelope in pNL/HXB) for mouse experiment	Paul D. Bieniasz, The Rockefeller University; Zhang et al., 2002	N/A

**Biological Samples**		

PBMCs from Donor 27845	M. Juliana McElrath, Fred Hutchinson Cancer Research Center	N/A
Plasma from Donor 27845	M. Juliana McElrath, Fred Hutchinson Cancer Research Center	N/A
Human cord blood/placental tissue (for isolation of Human CD34+ cells)	Department of Gynecology and Obstetrics, University Hospital of Cologne	N/A

**Chemicals, Peptides, and Recombinant Proteins**		

Dulbecco’s Modified Eagle Medium (DMEM)	GIBCO	Cat# 11960-044
Fetal bovine serum (FBS)	Sigma-Aldrich	Cat# F9665
Penicillin/Streptomycin	GIBCO	Cat# 15140-122
Sodium Pyruvate	GIBCO	Cat# 11360-070
L-Glutamine	Thermo Fisher Scientific	Cat# 25030024
Gentamicin	Sigma-Aldrich	Cat# G1397-10ML
HEPES	Biochrom	Cat# L1613
Freestyle 293 Expression Medium	Thermo Fisher Scientific	Cat# 12338001
Interleukin 2 (IL-2)	Roche	Cat# 11147528001
Interleukin 21 (IL-21)	Life Technologies	Cat# PHC0211
Streptavidin-PE	BioLegend	Cat# 405203
BG505-SOSIP.664.Avi	John P. Moore, Weill Cornell Medical College; Sok et al., 2014	N/A
Superscript III Reverse Transcriptase	Thermo Fisher Scientific	Cat# 18080044
RNasin Plus RNase inhibitor	Promega	Cat# N2615
Random primers	Invitrogen	Cat# 48190-011
HotStarTaq DNA Polymerase	QIAGEN	Cat# 203203
Polyethylenimine (PEI), branched 25 kDa	Sigma	Cat# 408727
Protein G Sepharose 4 Fast Flow	GE Healthcare	Cat# 17-0618-05
BG505-SOSIP.664.His	John P. Moore, Weill Cornell Medical College; de Taeye et al., 2015, Sanders et al., 2013	N/A
YU2 gp120 monomer	John R. Mascola, NIH	N/A
YU2 gp120 monomer mutants (N160K, N332A, D368R)	Michel C. Nussenzweig, The Rockefeller University	N/A
YU2 gp140 foldon trimer	Richard Wyatt, The Scripps Research Institute; Yang et al., 2000	N/A
YU2 gp140 foldon trimer mutants (A281T+368K, N160K+A281T+368K)	Michel C. Nussenzweig, The Rockefeller University	N/A
ABTS 1-Step Solution	Thermo Fisher Scientific	Cat# 002024
Peroxidase Streptavidin	Jackson Immuno	Cat# 016-030-084
T4 DNA Polymerase	New England Biolabs	Cat# M0203L
Platinum Taq Green Hot Start	Thermo Fisher	Cat# 11966034
Fugene 6 Transfection Reagent	Promega	Cat# E2691
B41 SOSIP.664 v4.2	Pugach et al., 2015	N/A
SF12 Fab	Michel C. Nussenzweig, The Rockefeller University (This Paper)	N/A
10-1074 Fab	Mouquet et al., 2012	N/A

**Critical Commercial Assays**		

LIVE/DEAD Fixable Aqua Dead Cell Stain Kit	Invitrogen	Cat# L34957
TOPO TA cloning Kit	Thermo Fisher Scientific	Cat# K457501
BirA-500: BirA biotin-protein ligase standard reaction kit	Avidity	Cat# BirA500
FluoReporter Mini-Biotin-XX Protein Labeling Kit	Thermo Fisher Scientific	Cat# F6347
QuikChange II XL Site-Directed Mutagenesis Kit	Agilent	Cat# 200521
Q5-Site-Directed Mutagenesis Kit	New England BioLabs	Cat# E0554S
NOVA Lite® HEp-2 ANA IgG (H&L) Immunoglobulin (External Evan’s Blue)	Inova Diagnostics	Cat# 704230

**Deposited Data**		

Silent face antibody family nucleotide sequences	GenBank	GenBank: MK722158–MK722171
SF12–B41 SOSIP.664–10-1074 coordinates	PDB	PDB: 6OKP
SF12 Fab coordinates	PDB	PDB: 6OKQ
SF12–B41 SOSIP.664–10-1074 complex cryoEM maps (class 1 and class 2)	EMDB	EMDB: 20100, 20101

**Experimental Models: Cell Lines**		

Mouse: 3T3-msCD40L Cells	NIH AIDS Reagent Program	Cat# 12535
Human: HEK293EBNA1-6E (293-6E)	National Research Council Canada	NRC File 11565
Human: HEK293T	ATCC	Cat# CRL-11268
Human: HeLa-derived TZM-bl	NIH AIDS Reagent Program	Cat# 8129
CHO Flp-In™ cells	Invitrogen	Cat# R75807

**Experimental Models: Organisms/Strains**		

NOD-Rag1^null^ IL2rg^null^ (NRG) mice	The Jackson Laboratory	Stock No. 007799

**Oligonucleotides**		

Human immunoglobulin variable region amplification primers for VH-, Vκ−, and Vλ	Michel C. Nussenzweig, The Rockefeller University; Scheid et al., 2011, Doria-Rose et al., 2015	N/A
HIV-1 qPCR Primer and Probe Set	Michel C. Nussenzweig, The Rockefeller University; Horwitz et al., 2017	N/A
HIV-1 YU2 env single genome sequencing primers	Michel C. Nussenzweig, The Rockefeller University; Horwitz et al., 2017	N/A

**Recombinant DNA**		

Human Expression vectors Igγ1, Igκ, Igλ, Ig-Fab heavy chain	Michel C. Nussenzweig, The Rockefeller University; Tiller et al., 2008	N/A
HIV-1_BG505.T332N_ gp160 env expression plasmid	Rogier W. Sanders, Academic Medical Center, Netherlands	N/A
HIV-1_BG505.T332N_ gp160 env expression plasmids carrying silent face antibody binding mutations	This Paper	N/A
HIV-1_YU2_ Env gp160 env expression plasmid	Joseph Sodroski, Dana-Farber Cancer Institute	N/A
HIV-1 Env YU2 expression plasmid carrying silent face antibody binding mutations	This Paper	N/A
HIV-1 SG3 ΔEnv Non-infectious Molecular Clone (pSG3ΔEnv)	NIH AIDS Reagent Program	Cat# 11051

**Software and Algorithms**		

IgBLAST	National Library of Medicine; Ye et al., 2013	https://www.ncbi.nlm.nih.gov/igblast/
IMGT	International ImMunoGeneTics Information System; Lefranc et al., 2009	http://www.imgt.org
Geneious v8.1.9	Biomatters Ltd.	N/A
Prism 7	GraphPad	N/A
Pymol	Schrodinger, LLC 2015	RRID: SCR_000305
UCSF Chimera	Pettersen et al., 2004	https://www.cgl.ucsf.edu/chimera/
Phenix	Adams et al., 2010	https://www.phenix-online.org
Coot	Emsley and Cowtan, 2004	http://www2.mrc-lmb.cam.ac.uk/personal/pemsley/coot/
Relion	Scheres, 2012	https://www2.mrc-lmb.cam.ac.uk/relion/index.php?title=Main_Page
CCP4 suite	Winn et al., 2011	http://www.ccp4.ac.uk/index.php
XDS	Kabsch, 2010	http://xds.mpimf-heidelberg.mpg.de/
Antibody Database v2.0	Pamela J. Bjorkman, California Institute of Technology; West et al., 2013	N/A

**Other**		

HiLoad 16/600 Superdex 200 pg column	GE Healthcare	Cat# 28989335
2G12 5 ml column made in-house using using NHS-activated HP resin and 2G12 IgG	GE Healthcare	Cat# 17071601
Protein A column	GE Healthcare	Cat# 17040301
300 Mesh Quantifoil R2/2 copper grids	EM Resolutions	QR22300Cu25

### Contact for Reagent and Resource Sharing

Further information and requests for resources and reagents should be directed to and will be fulfilled by the Lead Contact, Pamela J. Bjorkman (bjorkman@caltech.edu). The Bjorkman laboratory cannot lawfully distribute clones in the pTT5 vector. Those wishing to obtain these clones must first obtain a license from the National Research Council of Canada.

### Experimental Model and Subject Details

#### Human subjects

Donor 27845 is an adult male who was diagnosed with HIV-1 in 1985. He was a study participant in Seattle Vaccine Unit Observational Protocols “Immune Determinants Favoring Non-Progression in HIV-1 Infection” and “Evaluation of HIV-Specific Immunological and Virological Responses of HIV-1 Multiply-Exposed Seronegative Individuals” (P.I. MJ McElrath) at the Fred Hutchinson Cancer Research Center and was followed from 1998 - 2006. Apart from an NIH interventional study during which Donor 27845 started and stopped anti-retroviral therapy (ART) from 1998-2001, the subject has been off ART. Samples for this study were obtained from 2005 and 2006. During the time of follow-up, viral loads ranged from 35 – 23,300 copies/ml (median: 1,640 copies/ml) and CD4^+^ T cell counts ranged from 291 to 1,000 cells/mm^3^ (median: 590 cells/mm^3^). Studies and procedures were approved by the Fred Hutchinson Cancer Research Center Internal Review Board (FWA00001920). Samples for analysis were obtained under protocol MNU-0628 approved by the Rockefeller University Institutional Review Board.

#### Humanized mice

NOD-Rag1^null^ IL2rg^null^ (NRG) mice were purchased from The Jackson Laboratory, and were subsequently bred and maintained in the Dezentrales Tierhaltungsnetzwerk Weyertal at University of Cologne. NRG mice were fed ssniff complete feed 1124 during breeding and ssniff complete feed 1534 during maintenance, and kept under a 12 hr light/dark cycle with specific-pathogen-free (SPF) conditions. To determine the pharmacokinetics of antibody SF12, 6-week old non-humanized NRG mice (n = 3 per antibody, male and female mice in both groups) were injected intravenously via the tail-vein with 250 μg of antibody (SF12 or 3BNC117). Mice were bled on days 1, 3, 6, 9 and 14 after injection from the facial vein into Z-Gel Serum tubes (Sarstedt). Serum levels were determined using a previously described total IgG ELISA (Klein et al., 2012). Humanized mice for treatment experiments were generated using a previously described protocol with slight modifications (Klein et al., 2012, Traggiai et al., 2004). In brief, 1-5 days old NRG mice were sublethally irradiated, and 3-6 hours later injected intrahepatically with CD34^+^ hematopoietic stem cells. CD34+ cells were enriched by magnetic bead-based positive selection (Miltenyi) from PBMCs obtained from human cord blood and by placental perfusion under a protocol approved by the ethics committee of the Medical Faculty of the University of Cologne (protocol #16-110). All cord blood and tissue donors provided written informed consent. Humanization screening was performed at 12 weeks post injection by flow cytometry as previously described (Klein et al., 2012). For treatment experiments, humanized mice were infected with HIV-1_YU2_ (Zhang et al., 2002) (produced in 293T cells) intraperitoneally (Horwitz et al., 2013, Klein et al., 2012). To determine viral loads, plasma viral RNA was measured using a quantitative PCR (qPCR) assay (Horwitz et al., 2013, Horwitz et al., 2017) based on pol using primers HIV-1 Pol region F 5′-TAATGGCAGCAATTTCACCA-3′ and HIV-1 Pol region R 5′-GAATGCCAAATTCCTGCTTGA-3′, and probe 5′-/56-FAM/CCCACCAAC/ZEN/ARGCRGCCTTAACTG/3IABkFQ/-3′. The limit of accuracy of the assay (based on the standard curve used) was 384 copies/ml. Plasma viral loads were determined twice before experiment start and only mice with viral loads over 4,000 copies/ml were included in experiments. Male and female mice (18-67 weeks old) were used for treatment experiments. Treatment groups were matched primarily based on viral load and stem cell donor (previously identified as key determinants of viral load kinetics), with equal or comparable distributions of age and sex across groups. For antibody treatments, 1 mg of each antibody was administered subcutaneously as a loading dose followed by twice-weekly injections of 0.5 mg of each antibody in PBS subcutaneously for a total of 3 weeks (monotherapy) or 5 weeks (tri-mix). All mouse experiments were authorized by the State Agency for Nature, Environment and Consumer Protection (LANUV) of North Rhine-Westphalia.

#### Cell lines

HEK293T cells were obtained from the American Type Culture Collection (ATCC) and maintained in Dulbecco’s modified Eagle Medium (DMEM, GIBCO) with 10% fetal bovine serum (FBS, Sigma Aldrich), 1x Penicillin/Streptomycin (GIBCO), 1 mM Sodium Pyruvate (GIBCO), 2 mM L-Glutamine (Thermo Fisher Scientific) at 37°C/5% CO_2_. The HeLa-derived TZM-bl reporter cell line was sourced from the NIH AIDS Reagent Program and maintained in DMEM containing 10% fetal bovine serum, 1 mM Sodium Pyruvate, 2 mM L-Glutamine (Thermo Fisher Scientific), 50 μg/ml Gentamicin (Sigma-Aldrich), and 25 mM HEPES (Biochrom) at 37°C/5% CO_2_. HEK293EBNA1-6E (293-6E) cells were obtained from the National Research Council Canada (NRC) and maintained in Freestyle 293 Expression Medium (Thermo Fisher Scientific) containing 0.2% Penicillin/Streptomycin at 37°C /5% CO_2_ with shaking at 90-120 rpm. The sex of these cell lines is unknown. CHO Flp-In™ cells (Invitrogen) were a kind gift from the lab of John Moore (Cornell University) and maintained in Ham’s F-12 Medium supplemented with 10% heat-inactivated FBS (Sigma-Aldrich), 200 U/ml penicillin/streptomycin, 2 mM L-glutamine, 20 mM HEPES, 0.1 mM non-essential amino acids, 1 mM sodium pyruvate (GIBCO), and further supplemented with 100 μg/ml Zeocin (Invitrogen).

### Methods Details

#### IgG isolation for polyclonal IgG neutralization testing

IgG from subject 27845 was purified from heat-inactivated (1h 56°C) plasma (late 2005 time point) using Protein G Sepharose 4 Fast Flow (GE Healthcare), buffer exchanged into phosphate buffered saline (PBS) using an Amicon Ultra 30 kDa (Millipore), and sterile-filtered.

#### Neutralization fingerprinting analysis

Neutralization fingerprinting (Figure 1B) of the polyclonal antibody response of subject 27845 was done using a panel of 20 diverse HIV-1 strains (Doria-Rose et al., 2017). In brief, the neutralization fingerprint of a serum/polyclonal IgG (the potency-defined pattern of neutralization of a set of diverse viral strains) is represented as a combination of the neutralization fingerprints of a reference set of bNAbs, grouped in ten epitope-specific clusters. Using this method, the prevalence of each of the ten antibody groups can be estimated for the given serum/polyclonal IgG, with prevalence scores ranging between 0 (low) and 1 (high). Additionally, two measures (Residual score and Median of scores) are computed as a way to estimate prediction confidence for the prevalence scores (Georgiev et al., 2013, Raju et al., 2019). For neutralization fingerprinting of monoclonal antibodies (Figure 1F), a set of 80 viruses for which data was available for all antibodies was used. The tree was constructed with a distance metric based on the similarity of the neutralization patterns of the different antibodies. First, the correlations between the neutralization fingerprints (the antibody-specific pattern of neutralization of a set of diverse HIV-1 strains) were computed for each pair of antibodies. The antibody-antibody correlation matrix was then used as input to a hierarchical clustering algorithm to generate a neutralization fingerprinting-based antibody tree. Generally, antibodies that cluster closely together in the tree may indicate similar patterns of neutralization sensitivity/resistance for the given set of strains.

#### B cell microculture

Sorting and culturing of memory B cells was performed according to a previously published protocol (Doria-Rose et al., 2015, Huang et al., 2013). In brief, peripheral blood mononuclear cells (PBMCs) were stained with LIVE/DEAD Fixable Aqua (Invitrogen), CD19-PE-Cy7, CD16-Pacific Blue, CD3-APC-Cy7, IgM-PE-Cy5 and IgD-FITC. Gating was done on IgM^-^ and IgD^-^ negative B cells, and these were bulk sorted using a FACS Aria II cytometer. Bulk B cells were then diluted and plated at 2 B cells per well in 384-well plates. B cells were cultured for two weeks in the presence of IL-2 (Roche), IL-21 (Life Technologies) and CD40L-expressing NIH 3T3 cells as described (Doria-Rose et al., 2015, Huang et al., 2013). To assess culture success, a total IgG ELISA was performed on supernatants after two weeks of culture to determine the number of wells with positive IgG production. Cell supernatants were then screened in a microneutralization TZM.bl assay against viruses BaL.26 and BG505.T332N. Wells with neutralization > 50% against one or both strains were amplified using various sets of previously described primer sets for heavy chain and light chain (Doria-Rose et al., 2015, Scheid et al., 2011). Clone specific primers in the leader region were designed when necessary to obtain fully native sequences of the entire framework 1 region (FWR1). Positive bands were sanger sequenced using reverse amplification primers. In the case that multiple B cells were present (double peaks were obtained), bands were subcloned using the TOPO-TA kit (Invitrogen), colony PCR was performed, and bands were again sequenced by Sanger sequencing. Antibody sequences were analyzed using both IgBLAST and the international ImMunoGeneTics information system (IMGT) (Lefranc et al., 2009, Ye et al., 2013). Obtained heavy and light chain genes were cloned into human Igγ1-, Igκ or Igλ-expression vectors using sequence and ligation independent cloning (SLIC) (Jeong et al., 2012, Tiller et al., 2008, von Boehmer et al., 2016). The correct heavy and light chain pairing of the SF5/SF12 antibody family was confirmed by single B cell BG505 bait sorting data.

#### Single B cell bait-sorting

BG505 SOSIP.664-Avi for B cell sorting was produced in CHO cells and purified using a PGT145 immunoaffinity column as described (Pugach et al., 2015, Sok et al., 2014). Biotinylation of BG505 was done using BirA-ligase (Avidity) according to the manufacturer’s instructions. An aliquot of BG505 SOSIP.664-Avi-biotin was freshly coupled to Streptavidin-PE (Invitrogen) using 2.5 μg and 1 μl (0.2 mg/ml) of Streptavidin-PE in a total volume of 10 ul PBS. For the sort, 20 million PBMCs were freshly thawed and stained with the following fluorophore-coupled anti-human antibodies: IgG-APC, IgM-BV605, CD19-BV421, CD20-BV421, CD3-PerCP-Cy5.5, CD14 PerCP-Cy5.5, CD335 PerCP-Cy5.5, CD606 PerCP-Cy5.5 and 1:20 Streptavidin-PE coupled BG505.SOSIP.664 mix described above. Staining was performed for 30 mins at 4°C. Sorting was done on a FACS Aria II. The gating first included singlets, followed by exclusion of unwanted cells (CD3^-^, CD14^-^, CD335^-^, CD606^-^), selection for B cells (CD19^+^, CD20^+^) and finally sorting of single IgG^+^ PE^+^ cells into 96-well plates containing lysis buffer. B cell antibody genes were amplified and Sanger sequenced. Antibody sequences were analyzed using both IgBLAST and the international ImMunoGeneTics information system (IMGT) (Lefranc et al., 2009, Ye et al., 2013). Sequences of interest were cloned into human Igγ1-, Igκ or Igλ-expression vectors by SLIC as described above.

#### Phylogenetic analysis of SF family heavy chain sequences

The IgV_H_4^∗^59^∗^01 *Homo sapiens* allele sequence was obtained from the international ImMunoGeneTics information system (IMGT) (Lefranc et al., 2009). Antibody heavy chain nucleotide sequences of the SF family were aligned with the IgV_H_4^∗^59^∗^01 sequence in Geneious R8 (v8.1.9) using ClustalW. The maximum-likelihood tree was generated using the RAxML plugin (v 7.2.8) with a GTR Gamma model using the ‘Rapid Bootstrapping and search for best-scoring ML tree’ function with 100 bootstrap replicates. The best-scoring ML tree was then formatted using FigTree (v1.4.3).

#### Antibody production for ELISA, neutralization assays and *in vivo* experiments

293-6E cells were maintained in Freestyle 293 Expression Medium (Thermo Fisher Scientific) containing 0.2% Penicillin-Streptomycin (Thermo Fisher Scientific). Paired heavy and light chain expression constructs were transfected into 293-6E cells (NRC) using branched polyethylenimine (PEI) 25 kDA (Sigma). After 7 days of culture, cells were spun down at 4200 g for 40 mins at 4°C and supernatants were filtered through 0.22 μM aPES (Thermo Nalgene Rapid-Flow). Antibodies were then purified from filtered supernatants using Protein G Sepharose 4 Fast Flow (GE Healthcare) according to standard protocols. Antibodies were buffer exchanged and concentrated into PBS using Amicon Ultra centrifugal filter (Millipore) with either a 30 or 50 kDA molecular weight cutoff (MWCO).

#### Enzyme-linked immunosorbent assay (ELISA) of (mutant) YU2 gp120/gp140 proteins

Wild-type and mutant His-tagged YU2 gp120/gp140 proteins were expressed by transient transfection of 293-6E cells and purified using Ni-NTA according to manufacturer’s instructions. Corning Costar 96-Well Assay high-binding plates were coated for 1h at 37°C with 2 μg/ml of the respective protein (YU2 gp120 WT, YU2 gp120 D368R_gp120_, YU2 gp120 N332A_gp120_, YU2 gp120 N160K_gp120_ and YU2 gp140 WT, YU2 gp140 A281T_gp120_/D368K_gp120_, YU2 gp140 N160K_gp120_ A281T_gp120_/D368K_gp120_ and N332K_gp120_ (triple mutant)) using a volume of 50 μl/well. Plates were washed 6x using PBS-Tween20 (0.05%), and subsequently blocked using 3% BSA in PBS for 1h at 37°C (200 μl/well). After washing, serially-diluted antibodies were added (starting at 4 or 10 ug/ml, 1:3 dilution series) at 50 μl/well in 1% PBS/BSA and incubated for 1h at room temperature or 37°C. After another wash step, anti-human IgG (Southern Biotech or Jackson Immunoresearch) was added at 1:5000 (50 μl/well) in 1% PBS/BSA for 30 mins at 37°C. Development was done using 100 μl/well ABTS 1-Step Solution (Thermo Fisher Scientific), and absorbance was measured at 405 nm on a FluoStar Omega or 415 nm on a Tecan Sunrise.

#### BG505 SOSIP.664-His ELISAs

Corning Costar 96-Well Assay high-binding plates were coated overnight at room temperature or for 1h at 37°C with 2 μg/ml anti-His-tag antibody (Abcam) in PBS (50 μl/well). Plates were washed 6x using PBS-Tween20 (0.05%), and subsequently blocked using 3% BSA in PBS or 2% milk powder in PBS for 1h at 37°C (200 μl/well). After washing, purified BG505 SOSIP.664-His (de Taeye et al., 2015, Sanders et al., 2013) was added at 2 μg/ml in 1% BSA in PBS (50 μl/well), and incubated for 1h at 37°C, followed by another washing step. Next, serially-diluted antibodies were added (starting at 4 or 10 ug/ml, 1:3 dilution series) at 50 μl/well in 1% PBS/BSA, and incubated for 1h at room temperature or 37°C. After washing, anti-human IgG (Southern Biotech) was added at 1:5000 in 1% BSA in PBS (50 μl/well) for 30 mins at 37°C. Post washing, development was done using 100 μl/well ABTS 1-Step Solution (Thermo Fisher Scientific), and absorbance was measured at 405 nm on a FluoStar Omega or 415 nm on a Tecan Sunrise.

#### Competition ELISAs

Antibodies SF5 and SF12 were biotinylated using the FluoReporter Mini-Biotin-XX Protein Labeling Kit (Thermo Fisher Scientific). Corning Costar 96-Well Assay high-binding plates were coated overnight at room temperature or for 1h at 37°C with 2 μg/ml anti-His-tag antibody (Abcam) in PBS (50 μl/well). Plates were washed 6x using PBS-Tween20 (0.05%), and subsequently blocked using 3% BSA in PBS for 1h at 37°C (200 μl/well). After washing, BG505 SOSIP.664 was added at 2 μg/ml in 1% BSA in PBS (50 μl/well), and incubated for 1h at 37°C, followed by another washing step. Next, serially-diluted competitor antibodies (starting at 32 μg/ml, 1:3 dilution series) were added at 50 μl/well in 1% PBS/BSA and incubated for 1h at room temperature. Plates were washed and biotinylated SF5 or SF12 were added at 0.5 μg/ml (50 μl/well in 1% PBS/BSA) and incubated for 1h room temperature. After another wash step, Streptavidin-HRP (1:1000) was added at 50 μl/well in 1% PBS/BSA for 30 mins at room temperature. Development was done using 100 μl/well ABTS 1-Step Solution (Thermo Fisher Scientific), and absorbance was measured at 405 nm on a FluoStar Omega or 415 nm on a Tecan Sunrise.

#### Generation of mutant HIV_YU2_ and HIV_BG505_ pseudoviruses

Point mutations were introduced into the HIV_YU2_ and HIV_BG505T332N_ gp160 expression plasmids using the QuikChange site-directed mutagenesis kit (Agilent Technologies) or the Q5 Site-directed mutagenesis kit (NEB) according to manufacturer’s specifications. Pseudoviruses were produced by co-transfection with pSG3ΔEnv into HEK293T according to an established protocol (Sarzotti-Kelsoe et al., 2014).

#### *In vitro* neutralization assays

Neutralization activities of polyclonal IgG and monoclonal antibodies were determined using a luciferase-based TZM.bl assay (Li et al., 2005, Sarzotti-Kelsoe et al., 2014, Seaman et al., 2010), which measures the reduction of Tat-induced luciferase expression in TZM-bl reporter cells during a single round of infection. Samples were assayed at least in in duplicate. Polyclonal IgG neutralization assays were done using a starting concentration of 500 μg/ml and monoclonal antibodies were assayed at starting concentrations of 10, 25 or 50 μg/ml. IC_50_s and IC_80_s were derived 5-parameter curve fitting. Neutralization was also assessed against murine leukemia virus (MuLV) to detect unspecific activity (Sarzotti-Kelsoe et al., 2014). Neutralization data for Figures 1E and 2E were analyzed and graphed using Antibody Database (v 2.0) (West et al., 2013).

#### Autoreactivity and polyreactivity assays

Autoreactivity of antibodies SF5 and SF12 and reference antibodies 4E10 and 2F5 was determined using the commercially-available HEp-2 based assay NOVA Lite kit (Inova Diagnostics) at an IgG concentration of 25 μg/ml. Slides were photographed on a Leica DMI 6000 B with an exposure of 800 ms, Gain of 10 and Intensity of 100%. Measurements were done in duplicate. Representative images are shown in Figure S1.

Polyreactivity assays were conducted using ELISA detection of non-specific binding to baculovirus extracts as described (Hötzel et al., 2012). Briefly, a solution of 1% baculovirus particles in 100mM sodium bicarbonate buffer pH 9.6 was absorbed onto the wells of a 384-well ELISA plate (Nunc Maxisorp) using a Tecan Freedom Evo liquid handling robot, and the plate was incubated overnight at 4°C. The plate was then blocked with 0.5% BSA in PBS for 1 hour at room temperature. Purified IgGs (diluted to 1 μg/mL in PBS, 0.5% BSA) were added to the blocked assay plate and incubated for 3 hours at room temperature. Bound IgG was detected as the luminescence signal at 425 nm using an HRP-conjugated anti-human IgG (H&L) secondary antibody (Genscript) and SuperSignal ELISA Femto Maximum Sensitivity Substrate (Thermo Fisher Scientific).

#### Single genome sequencing of plasma HIV-1 Env genes

Amplification of genes encoding HIV-1 Env gp160 from single viral genomes was carried out as described previously with slight modifications (Keele et al., 2008, Salazar-Gonzalez et al., 2008). In brief, complementary DNA (cDNA) was synthesized using primer YB383 5′-TTTTTTTTTTTTTTTTTTTTTTTTRAAGCAC-3′ and Superscript III (Invitrogen) according to manufacturer’s instructions. cDNA was then serially diluted and HIV-1 Env was amplified in two rounds of nested PCR using Platinum Taq Green Hot Start (Thermo Fisher Scientific) and primers specifically adapted for HIV_YU2NL4-3_ (1^st^ round primers were YB383 and YB50 5′- GGCTTAGGCATCTCCTATGGCAGGAAGAA-3′; 2^nd^ round primers YB49 5′-TAGAAAGAGCAGAAGACAGTGGCAATGA-3′, YB52 5′-GGTGTGTAGTTCTGCCAATCAGGGAAGWAGCCTTGTG-3′). Positive Bands from amplifications with less than 30% efficiency were PCR-purified using the Nucleospin Gel and PCR-Clean Up kit (Macherey Nagel) and Sanger sequenced using a set of 8 primers. Env sequences were assembled using the Geneious 8.1.9 (Biomatters) de-novo assembly tool. Assembled sequences were cross-checked against sequencing traces again to validate assemblies, and sequences with full coverage of gp160 Env were used in downstream analyses.

#### Protein expression and purification for structural studies

Fabs from SF12, SF5 and 10-1074 IgGs were produced as described (Scharf et al., 2015). Briefly, Fabs were expressed by transiently transfecting HEK293-6E cells with vectors encoding the appropriate light chain and C-terminal 6x-His tagged heavy chain genes. Secreted Fabs were purified from cell supernatants using Ni^2+^-NTA affinity chromatography (GE Healthcare), followed by size exclusion chromatography (SEC) with a Superdex200 16/60 column (GE Healthcare). Purified Fabs were concentrated and maintained at 4°C in storage buffer (20 mM Tris pH 8.0, 150 mM NaCl, 0.02% sodium azide).

A gene encoding soluble B41 SOSIP.664 gp140 trimer, including SF501C_gp120,_ T605C_gp41_, and I559P_gp41_ substitutions, an enhanced gp120–gp41 cleavage site (REKR to RRRRRR), and a stop codon after residue 664_gp41_ (Env numbering according to HXB2 nomenclature), was stably expressed in Chinese hamster ovary cells as described (Chung et al., 2014, Pugach et al., 2015). Secreted Env trimers expressed in the absence of glycosylation inhibitors were isolated from cell supernatants using 2G12 immunoaffinity chromatography by covalently coupling 2G12 IgG monomer to an activated-NHS Sepharaose column (GE Healthcare) as described (Scharf et al., 2015). Trimers were eluted using 3M MgCl_2_ and immediately dialyzed into storage buffer before SEC purification with a Superdex200 16/60 column (GE Healthcare) against the same buffer. Peak fractions pertaining to SOSIP trimers were pooled and repurified using the same column and buffer conditions. Individual fractions were stored separately at 4°C.

#### Crystal structure of SF12 Fab

Purified Fab was concentrated to 10-15 mg/mL by centrifugation with a 30-kDa concentrator (Amicon). Initial matrix crystallization trials were performed at room temperature using the sitting drop vapor diffusion method by mixing equal volumes of protein sample and reservoir using a TTP LabTech Mosquito robot and commercially-available screens (Hampton Research and QIAGEN). Initial hits were optimized and crystals were obtained in 0.1 M HEPES pH 7.0, 1.6 M Sodium Formate at 20°C. Crystals were cryo-protected stepwise to 3.0 M Sodium Formate before being cryopreserved in liquid nitrogen.

X-ray diffraction data were collected for SF12 Fab at the Stanford Synchroton Radiation Lightsource (SSRL) beamline 12-2 on a Pilatus 6M pixel detector (Dectris). Data from a single crystal were indexed and integrated in XDS (Kabsch, 2010) and merged with AIMLESS in the *CCP4* software suite (Winn et al., 2011). Structures were determined by molecular replacement in PHASER (McCoy et al., 2007) using a single search with coordinates of the F105 Fab (PDB 1U6A), which had ∼85% sequence identity to SF12 after removal of CDR loops. Models were refined using B-factor refinement in CNS (Brunger, 2007) and Phenix (Adams et al., 2010), followed by several cycles of manual building with B factor sharpening in Coot (Emsley et al., 2010).

#### Cryo-EM sample preparation

Complexes of B41-SF12-101074 were assembled by incubating purified SF12 Fab with B41 SOSIP.664 trimer at a 1.2:1 Fab:gp120-protomer molar ratio. Following overnight incubation at RT, 10-1074 Fab was incubated with the complex at a 1.2:1 Fab:gp120-protomer molar ratio for 5 h. SF12–B41–10-1074 complexes were diluted to 0.5-0.8 mg/ml in TBS and 3μL was added to Quantifoil R2/2 300 mesh copper grids (Electron Microscopy Services) that had been freshly glow-discharged using a PELCO easiGlow (Ted Pella). Samples were immediately vitrified in 100% liquid ethane using a Mark IV Virtoblot (Thermo Fisher Scientific) by blotting for 2.5-4 s with Whatman No. 1 filter paper at 20°C and 100% relative humidity.

#### Cryo-EM data collection and processing

Single-particle cryo-EM data were collected on a Titan Krios transmission electron microscope (Thermo Fisher Scientific) operating at 300 kV, using the EPU automated image acquisition software (Thermo Fisher Scientific). Movies were collected on a Gatan K2 Summit direct electon detector (DED) operating in counting mode at a nominal magnification of 130,000x (1.09 Å/pixel) using a defocus range of −1.2 μm to −3.0 μm. Movies were collected over an 8 s exposure with an exposure rate of ∼4.8 e^-^ /pixel/s, resulting in a total dose of ∼40 e^-^/Å^2^.

Movies were motion corrected and doseweighted using the MotionCor2 frame alignment program in RELION-3 (Zivanov et al., 2018). Non-doseweighted summed images were used for CTF determination using Gctf (Zhang, 2016), and reference-free particle picking from 18 micrographs was achieved using Laplacian-of-Gaussian filtering in RELION-3 (Zivanov et al., 2018). An initial stack of 1,907 particles was 2D classified and the best classes representing top-down and side views of Env-trimers was used for subsequent automated template-based picking in RELION-3. 676,161 particles were extracted from 2,209 dose-weighted micrographs, binned 4x4 (4.36 Å/pixel), and subjected to reference-free 2D classification in RELION-3 and a 240 Å circular mask. A total of 371,665 particles corresponding to class averages that displayed secondary-structural elements and represented views different views of Fab bound Env-trimer were extracted and re-centered prior to heterogenous ab inito model generation using cryoSPARC v2.2 (Punjani et al., 2017).

The generated volume was low-passed filtered to 40 Å and used as an initial model for 3D auto-refinement in RELION-3. Due to the observed low occupancy of 10-1074 Fab, particles were re-extracted unbinned (1.09 Å/pixel) and 3D classified (C1 symmetry, k = 8) with a soft mask generated from the initial model (5-pixel extension, 10-pixel soft cosine edge). Classification resulted in two distinct classes comprising three or two SF12 Fabs bound per trimer and one 10-1074 Fab bound per trimer. Particles from each class were then separately refined, followed by 3D auto-refinement using a soft mask in which Fab constant domains were masked out. Class 1 (301,920 particles) refined to a final estimaled resolution of ∼3.28 Å (SF12_3_—B41–10-1074_1_; C1 symmetry) and class 2 (55,136 particles) refined to a final estimated resolution of ∼4.36 Å (SF12_2_—B41–10-1074_1_; C1 symmetry) according to gold-standard FSC (Bell et al., 2016).

#### Modeling and refinement of cryo-EM structures

For the final reconstruction of class 1 (SF12_3_—B41–10-1074_1_; C1 symmetry), initial coordinates were generated by docking reference models into the cryo-EM density using UCSF Chimera v1.13 (Goddard et al., 2007) (gp120-gp41, PDB 6CH9; 10-1074 Fab, PDB 4FQQ; SF12 Fab, this work). Initial models were then refined into the EM maps using one round of rigid body, morphing, and simulated annealing followed by subsequent rounds of B-factor refinement in Phenix (Adams et al., 2010). Models were manually built following iterative rounds of real-space and B-factor refinement in Coot (Emsley et al., 2010) and Phenix (Adams et al., 2010) with secondary structure restraints. Modeling of glycans was achieved by interpreting cryo-EM density at PNGS in Coot using a map with a −75 Å^2^ B-factor sharpening value, contoured at 6σ due to the lower resolution of glycans at the periphery of the structure. Validation of model coordinates was performed using MolProbity (Chen et al., 2010) and Privateer (Agirre et al., 2015).

#### Structural and bioinformatic analyses

Superpositions and figures were rendered using PyMOL (Version 1.5.0.4 Schrodinger, LLC), and protein electrostatic calculations were done using APBS and PDB2PQR webservers (Unni et al., 2011). Buried surface areas (BSAs) were determined with PDBePISA using a 1.4Å probe (Krissinel and Henrick, 2007). Potential hydrogen bonds were assigned using a distance of < 3.6Å and an A-D-H angle of > 90°, while the maximum distance allowed for a van der Waals interaction was 4.0Å. Putative H-bonds, van der Waals assignments and total BSA should be considered tentative, owing to the relatively low structure resolutions. Computational analysis of neutralization panel data (Table S7) was done as previously described (West et al., 2013). For determining the difference in orientation of the antibody variable domains of the SF12-Env and VRC-PG05-Env complexes, those structures were aligned on gp120, and then the transformation relating the V_H_-V_L_ domains was calculated by using TM-align (Zhang and Skolnick, 2005). The corresponding screw transformation was calculated as described (Siciliano and Khatib, 2008) and visualized using Antibody Database (West et al., 2013).

### Data and Software Availability

The accession numbers for the nucleotide sequences of SF-family members are GenBank: MK722158–MK722171. The accession numbers for the cryo-EM reconstructions of the SF12–B41–10-1074 complexes comprising three or two SF12 Fabs are Electron Microscopy Data Bank (EMDB): EMD-20100 and EMD-20101, respectively. The accession numbers for coordinates for atomic models of the cryo-EM SF12–B41–10-1074 complex (class 1: three SF12 Fabs and one 10-1074 Fab) and the unliganded SF12 Fab crystal structure are Protein Data Bank (PDB): PDB 6OKP and PDB 6OKQ, respectively.

## Supplemental Information

Document S1. Figures S1–S5 and Tables S1–S7

Document S2. Article plus Supplemental Information
