# Supplementary material for: Broad and Potent Neutralizing Antibodies Recognize the Silent Face of the HIV Envelope
Source: Immunity. 2019 Jun 18;50(6):1513–1529.e9. doi: 10.1016/j.immuni.2019.04.014 (PMC6591006; doi:10.1016/j.immuni.2019.04.014)
Supplement: Document S1. Figures S1–S5 and Tables S1–S7 [file mmc1.pdf]

Immunity, Volume *50*

Supplemental Information

Broad and Potent Neutralizing Antibodies

Recognize the Silent Face of the HIV Envelope

Till Schoofs, Christopher O. Barnes, Nina Suh-Toma, Jovana Golijanin, Philipp Schommers, Henning Gruell, Anthony P. West Jr., Franziska Bach, Yu Erica Lee, Lilian Nogueira, Ivelin S. Georgiev, Robert T. Bailer, Julie Czartoski, John R. Mascola, Michael S. Seaman, M. Juliana McElrath, Nicole A. Doria-Rose, Florian Klein, Michel C. Nussenzweig, and Pamela J. Bjorkman

Supplementary Figures

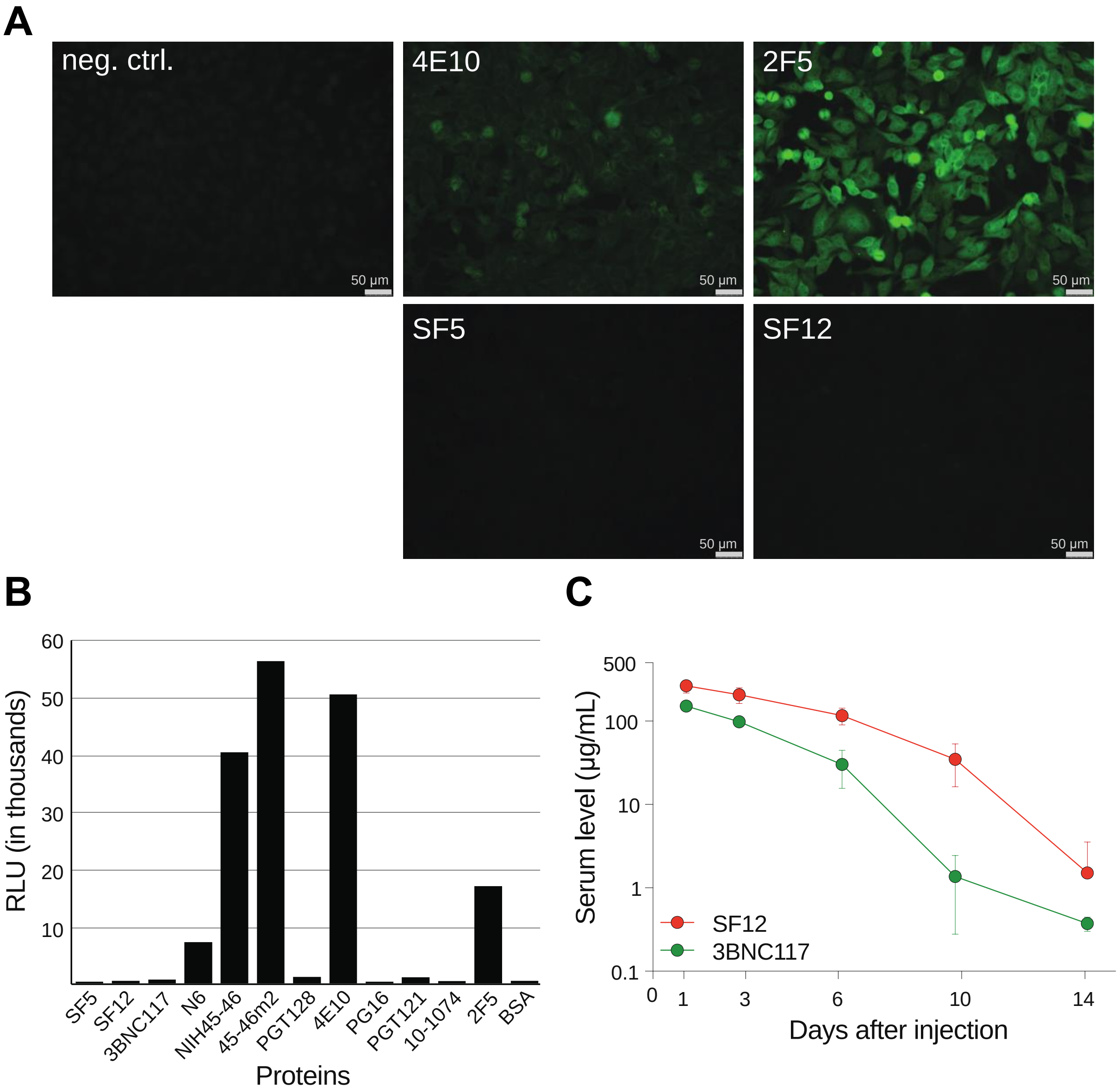

Figure S1. Polyreactivity and pharmacokinetics assays of SF12 and SF5 antibodies. Related to Figure 1. (A) HEp-2 assay for potential autoreactivity of indicated bNAbs. Data representative of 2 repeat assays. (B) Polyreactivity ELISA-based assay detecting non-specific binding of a panel of bNAbs and a control protein (BSA) to a baculovirus extract. (C) *In vivo* pharmacokinetics of SF12 IgG compared with 3BNC117 IgG measured in 6-week old non-reconstituted NRG mice (3 mice each antibody). One independent experiment. Shown is Mean of all 3 mice ± SD.

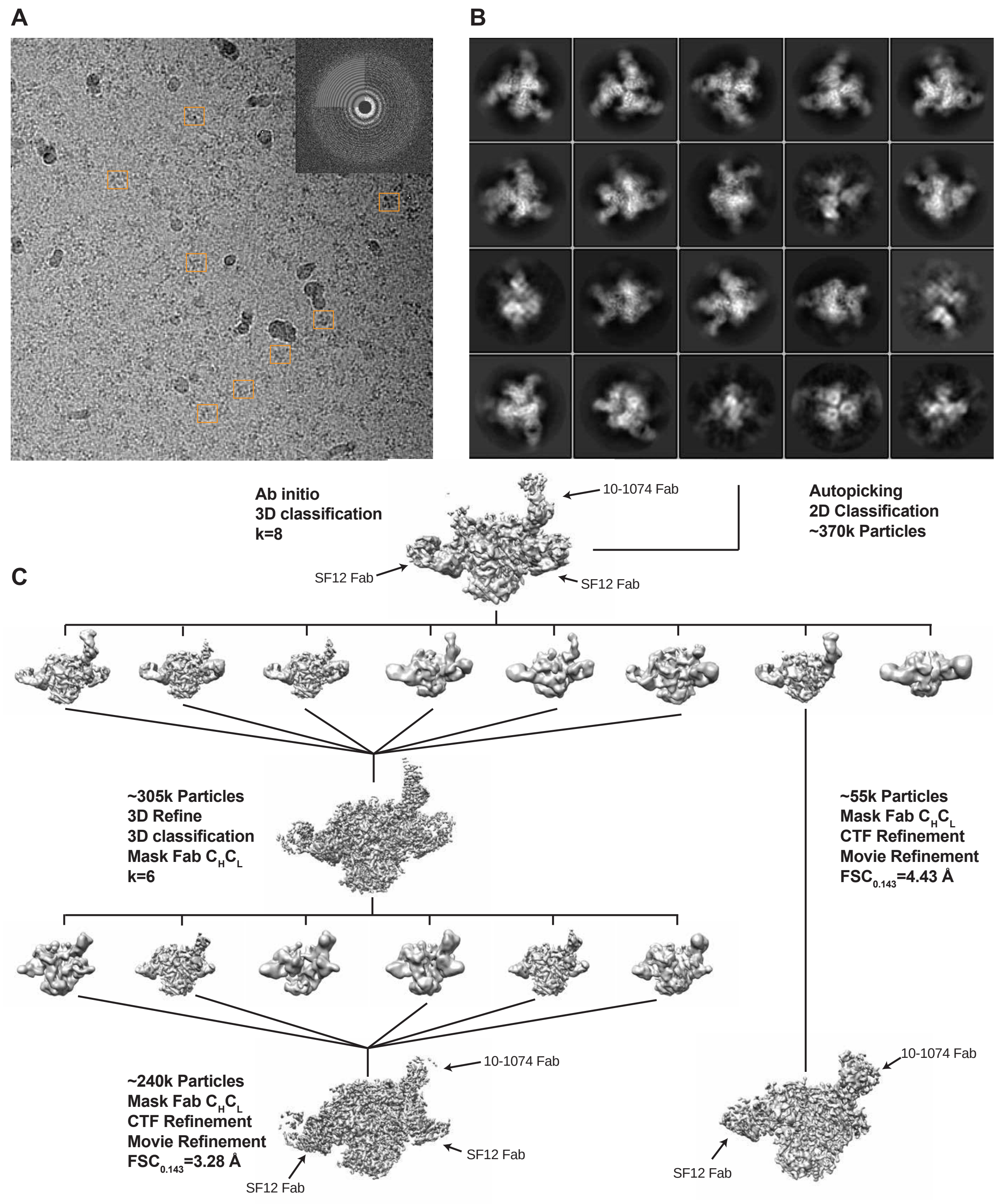

Figure S2. Data collection and processing for the SF12–B41–10-1074 complex. Related to Figure 3. (**A**) Representative micrograph of SF12-B41-10-1074 complex in vitreous ice with individual particles boxed (orange). Inset: power spectrum of micrograph determined during CTF estimation showing Thon rings to 3Å. (**B**) Reference-free 2D classification of 4x4 binned extracted particles. 2D class averages showing secondary structure were selected for further processing. (**C**) After 3D auto-refinement of ab initio model, good particles were subjected to rounds of 3D classification with C1 symmetry applied. 3D classes that showed similar features were pooled and 3D auto-refined to generate final reconstructions at 3.28Å and 4.36Å for class 1 and class 2, respectively.

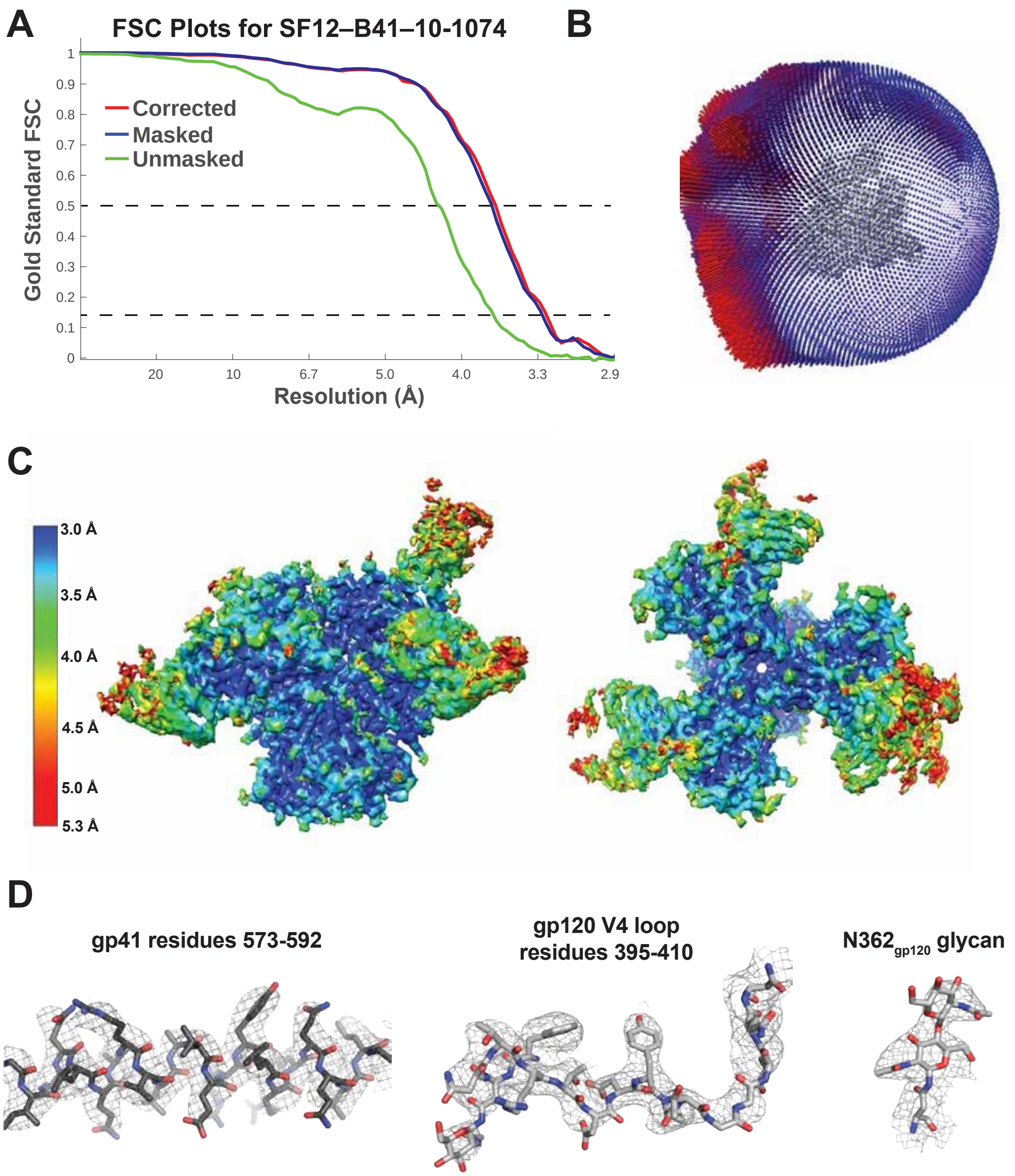

Figure S3. Validation and cryo-EM map quality. Related to Figure 3. (**A**) Fourier shell correlation (FSC) plots calculated from half-maps of masked (red), unmasked (blue), and corrected (black) data for Class 1. Dotted lines for FSC values of 0.5 and 0.143 are shown. (**B**) Angular distribution 3D histogram, (**C**) local resolution estimation (Resmap), and (**D**) representative density from Fab and Env regions of the Class 1 map.

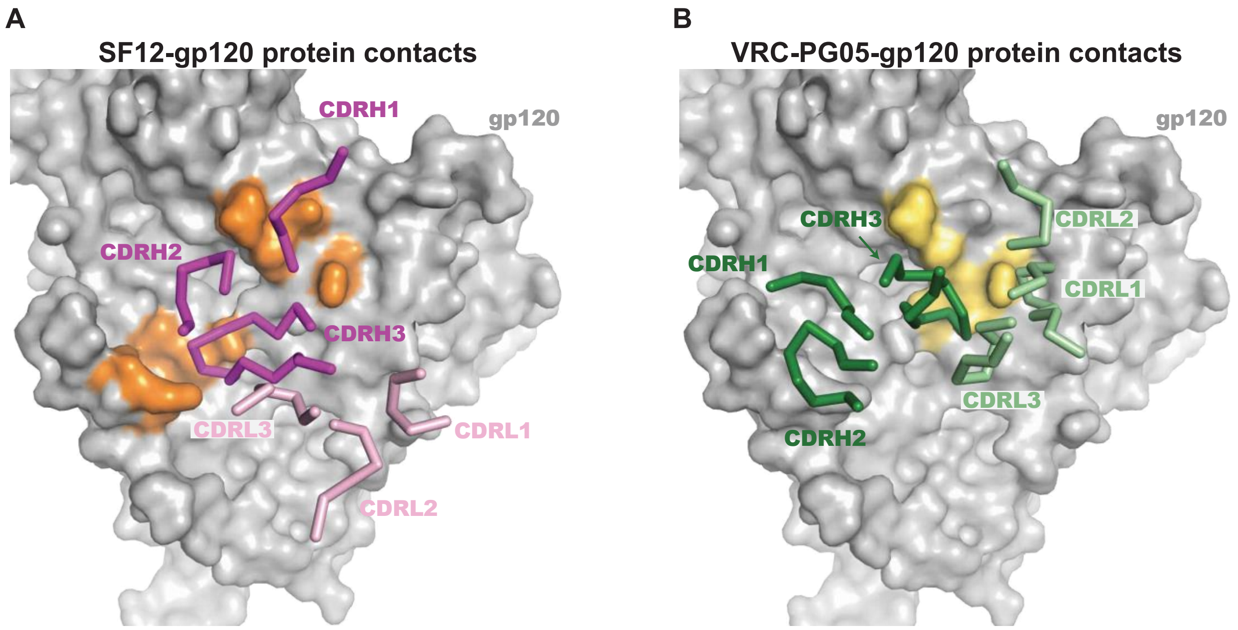

Figure S4. Comparison of SF12 and VRC-PG05 footprints on gp120. Related to Figure 3 and Figure 4. Differences in CDR loop orientations (ribbon) on gp120 (gray surface) by (**A**) SF12 (magenta, CDRH1-3; light pink, CDRL1-3) and (**B**) VRC-PG05 (forest green, CDRH1-3; light green, CDRL1-3). The protein epitope on the gp120 surface is highlighted in orange (SF12) or yellow (VRC-PG05). The glycan portion of the epitope was removed for clarity.

A

```
                                 -CDR1-                     --CDR2-                                      -----------N1-------------   -D3-3 -N2- -J5-2-
                           *    ^ **                     ^**^^** ^            ^*  ^                                #  #  #  #  # *  ^     #
4-59 iGL QVQLQESGPGLVKPSETLSLTCTVSGGSVSSYYWSWIRQPPGKGLEWIGYIYYSGS-TNYNPSLKSRVTISVDTSKNQFSLKLSSVTAADTAVYYCAR(L)(G)(P)(G)(G)(I)(F)(D)(R)(W)SGYY(G)(D)NWFDPW
SF12 mat ..................V..R.....LDL..............Q...FV.FD..YGD.D...R......A.M....I..R.K...P.......... .  .  .  .  .  .  .  .  .  . T.H. .  . K.L...
SF5  mat ..................V..R.....LDR...........E..Q...HV.FD..YGD.D...R.....TA.M..Y.II.R.R...P.......... .  .  .  .  .  .  .  .  .  . T.H. .  . K.L...

                                     ^                                                           *
3-20 iGL EIVLTQSPGTLSLSPGERATLSCRASQSVSSSYLAWYQQKPGQAPRLLIYGASSRATGIPDRFSGSGSGTDFTLTISRLEPEDFAVYYCQQYG(R)(R)(L)        *Contact mediated by SHM
SF12 mat Q.D.....R.....A......L........NVA.....H.........LHE..T.........I.....R......TS............LS.  .  .  .        ^Contact mediated by germline AA
SF5  mat Q.......R.....A......L........NVA.....H.........LHE..I..A......V.....R......TS.H..........LS.  .  .  .        #Contact mediated by N-region
```

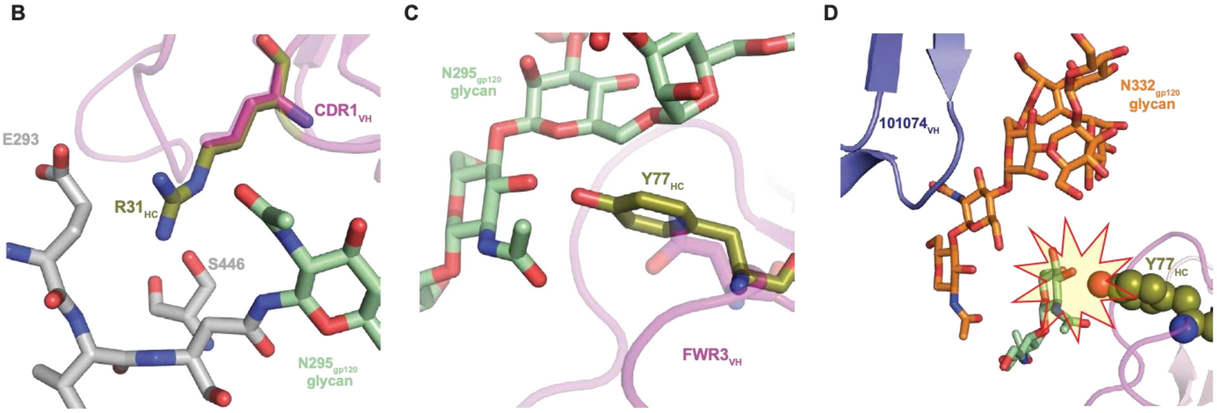

Figure S5. Comparison of SF12 and SF5 clonal variants. Related to Figure 2, Figure 5, and Figure 6. (**A**) Sequence alignment of SF12 and SF5 with deduced germline sequences. SHMs are shown for each antibody with paratope residues denoted. (**B, C**) Modeling of SF5 residues (olive) onto the SF12-Env structure. In each case, SHMs in SF5 potentially enhance recognition of the Env epitope through hydrogen bonding. (**D**) Modeling of SF5 $Y77_{HC}$ onto the 10-1074 bound protomer shows clashes between this residue and the slightly shifted $N295_{gp120}$ glycan (pale green).

Supplementary Tables

Table S1. Sequence analysis of silent face antibodies from donor 27845. Related to Figure 1.

	**Antibodies cloned from microculture double hits**									
	Heavy chain					Light chain				
MC Well	V-gene	% nt mutation	% AA mutation	CDRH3 (AA)	CDRH3 (seq)	V-gene	% nt mutation	% AA mutation	CDR3 (AA)	CDRL3 (seq)
SF3	IGVH4-59*01	25.3%	39.2%	23	GRVGPGGLFDRWRGYHGHKWVDA	IGKV3-20*01	21.4%	29.0%	6	QQYGRT
**SF5**	**IGVH4-59*01**	**19.0%**	**25.5%**	**23**	**ARLGPGGIFDRWTGHYGDKWLDP**	**IGKV3-20*01**	**16.0%**	**21.5%**	**6**	**QLSGRR**
SF7	IGVH4-59*01	25.3%	39.2%	23	GRVGPGGLFDRWRGYHGHKWVDA	IGKV3-20*01	21.4%	29.0%	6	QQYGRT
SF2	IGVH4-59*01	24.4%	32.0%	23	ARLGPGGLFDRYTGYHGRKWLDA	IGKV3-20*01	18.0%	23.9%	6	QQYGRT
SF8	IGVH4-59*01	24.4%	32.0%	23	ARLGPGGLFDRYTGYHGRKWLDA	IGKV3-20*01	16.5%	21.7%	6	QQYGRT
SF10	IGVH4-59*01	25.3%	37.1%	23	GRVGPGGLFDRWTGYHGHKWVDA	IGKV3-20*01	19.9%	29.0%	6	QQYGRT

	**Clone members isolated by BG505-sorting upon microculture identification of clone**									
	Heavy chain					Light chain				
Sort Well	V-gene	% nt mutation	% AA mutation	CDRH3 (AA)	CDRH3 (seq)	V-gene	% nt mutation	% AA mutation	CDR3 (AA)	CDRL3 (seq)
**SF12**	**IGVH4-59*01**	**17.0%**	**21.4%**	**23**	**ARLGPGGIFDRWTGHYGDKWLDP**	**IGKV3-20*01**	**14.6%**	**20.4%**	**6**	**QLSGRR**
SF10	IGVH4-59*01	25.3%	37.1%	23	GRVGPGGLFDRWTGYHGHKWVDA	IGKV3-20*01	19.9%	29.0%	6	QQYGRT

Table S2. 119 virus cross-clade panel neutralization data. Related to Figure 1.

Virus ID	Clade*	SF5 IC_{50}	SF5 IC_{80}	SF5 MPI	SF12 IC_{50}	SF12 IC_{80}	SF12 MPI
6535.3	B	0.13	0.48	100	0.11	0.58	100
QH0692.42	B	0.55	1.45	100	0.49	1.32	100
SC422661.8	B	0.19	0.64	100	0.20	0.69	100
PVO.4	B	0.08	0.31	100	0.07	0.35	100
TRO.11	B	0.44	2.32	99	0.35	1.40	100
AC10.0.29	B	0.03	0.08	100	0.01	0.06	100
RHPA4259.7	B	0.73	5.23	92	0.14	0.50	100
THRO4156.18	B	0.17	0.49	99	0.14	0.55	99
REJO4541.67	B	0.03	0.10	100	0.02	0.08	100
TRJO4551.58	B	0.63	2.18	99	0.33	0.92	100
WITO4160.33	B	>50	>50	14	0.75	15.37	88
CAAN5342.A2	B	0.04	0.11	100	0.12	0.25	100
WEAU_d15_410_787	B (T/F)	0.33	1.10	100	0.43	1.41	100
1006_11_C3_1601	B (T/F)	>50	>50	37	0.44	2.33	98
1054_07_TC4_1499	B (T/F)	0.12	0.82	100	0.21	1.58	100
1056_10_TA11_1826	B (T/F)	0.20	0.72	100	0.12	0.56	100
1012_11_TC21_3257	B (T/F)	>50	>50	17	0.23	1.11	100
6240_08_TA5_4622	B (T/F)	4.58	29.42	88	0.62	1.97	100
6244_13_B5_4576	B (T/F)	0.21	0.57	100	0.95	6.94	87
62357_14_D3_4589	B (T/F)	0.07	0.22	100	0.10	0.35	100
SC05_8C11_2344	B (T/F)	0.34	0.93	100	0.26	0.94	100
Du156.12	C	>50	>50	18	>25	>25	37
Du172.17	C	>50	>50	25	>25	>25	29
Du422.1	C	>50	>50	36	9.07	>25	73
ZM197M.PB7	C	0.19	0.67	99	10.48	>25	68
ZM214M.PL15	C	>50	>50	29	>25	>25	22
ZM233M.PB6	C	>50	>50	23	>25	>25	35
ZM249M.PL1	C	>50	>50	21	>25	>25	22
ZM53M.PB12	C	0.29	1.10	97	4.48	>25	75
ZM109F.PB4	C	0.10	0.22	100	0.24	0.88	99
ZM135M.PL10a	C	>50	>50	23	>25	>25	37
CAP45.2.00.G3	C	>50	>50	8	>25	>25	27
CAP210.2.00.E8	C	>50	>50	28	>25	>25	32
HIV-001428-2.42	C	0.04	0.11	100	0.05	0.20	100
HIV-0013095-2.11	C	>50	>50	13	>25	>25	21
HIV-16055-2.3	C	>50	>50	36	>25	>25	33
HIV-16845-2.22	C	>50	>50	35	4.10	>25	74
Ce1086_B2	C (T/F)	0.05	0.19	97	0.02	0.08	100
Ce0393_C3	C (T/F)	>50	>50	22	>25	>25	23
Ce1176_A3	C (T/F)	>50	>50	22	>25	>25	20
Ce2010_F5	C (T/F)	0.07	0.18	100	0.12	0.33	100
Ce0682_E4	C (T/F)	>50	>50	11	>25	>25	15
Ce1172_H1	C (T/F)	>50	>50	30	7.09	>25	76
Ce2060_G9	C (T/F)	26.93	>50	66	>25	>25	34
Ce703010054_2A2	C (T/F)	0.05	0.14	100	0.06	0.16	100
BF1266.431a	C (T/F)	7.51	>50	78	>25	>25	30
246F C1G	C (T/F)	>50	>50	14	>25	>25	14
249M B10	C (T/F)	>50	>50	1	>25	>25	6
ZM247v1(Rev-)	C (T/F)	>50	>50	39	>25	>25	25
7030102001E5(Rev-)	C (T/F)	>50	>50	23	>25	>25	21
1394C9G1(Rev-)	C (T/F)	2.25	18.61	87	2.03	23.15	81
Ce704809221_1B3	C (T/F)	34.15	>50	57	>25	>25	34
MS208.A1	A	>50	>50	22	>25	>25	23
Q23.17	A	0.27	7.04	90	0.11	0.68	95
Q461.e2	A	>50	>50	17	>25	>25	19
Q769.d22	A	0.27	>50	74	0.21	2.06	90
Q259.d2.17	A	1.18	>50	79	0.56	13.04	83
Q842.d12	A	0.07	0.26	100	0.07	0.25	100
0260.v5.c36	A	>50	>50	17	>25	>25	20
3415.v1.c1	A	>50	>50	10	>25	>25	14
3365.v2.c20	A	0.08	0.41	95	0.10	0.45	100
191955_A11	A (T/F)	0.58	3.01	95	0.16	0.52	100
191084 B7-19	A (T/F)	0.11	0.31	100	0.07	0.21	100
9004SS_A3_4	A (T/F)	6.51	>50	69	>25	>25	46

Virus ID	Clade*	SF5 IC_{50}	SF5 IC_{80}	SF5 MPI	SF12 IC_{50}	SF12 IC_{80}	SF12 MPI
CNE19	BC	>50	>50	31	>25	>25	38
CNE20	BC	>50	>50	25	>25	>25	39
CNE21	BC	>50	>50	25	>25	>25	28
CNE17	BC	0.09	0.31	100	0.11	0.38	100
CNE30	BC	>50	>50	13	>25	>25	12
CNE52	BC	>50	>50	8	>25	>25	8
CNE53	BC	46.80	>50	55	>25	>25	36
CNE58	BC	0.02	0.06	100	0.02	0.07	100
T257-31	CRF02_AG	>50	>50	11	>25	>25	4
928-28	CRF02_AG	>50	>50	15	>25	>25	10
263-8	CRF02_AG	3.00	17.90	89	4.19	24.51	80
T250-4	CRF02_AG	>50	>50	17	>25	>25	15
T251-18	CRF02_AG	>50	>50	49	12.13	>25	65
T278-50	CRF02_AG	>50	>50	29	>25	>25	50
T255-34	CRF02_AG	11.93	>50	62	>25	>25	34
211-9	CRF02_AG	16.28	>50	67	0.10	0.28	100
235-47	CRF02_AG	>50	>50	43	>25	>25	37
620345.c01	CRF01_AE	0.02	0.06	100	0.02	0.08	100
CNE8	CRF01_AE	0.05	0.18	99	0.12	0.59	97
C1080.c03	CRF01_AE	0.03	0.09	100	0.05	0.18	100
R2184.c04	CRF01_AE	0.05	0.14	100	7.43	>25	66
R1166.c01	CRF01_AE	0.22	0.59	97	0.61	2.59	93
R3265.c06	CRF01_AE	0.01	0.05	100	0.01	0.04	100
C2101.c01	CRF01_AE	0.03	0.09	100	0.04	0.19	100
C3347.c11	CRF01_AE	0.03	0.07	100	0.04	0.12	100
C4118.c09	CRF01_AE	0.06	0.20	98	0.10	0.45	98
CNE5	CRF01_AE	0.04	0.11	100	0.26	1.03	98
BJOX009000.02.4	CRF01_AE	0.02	0.06	100	0.02	0.07	100
BJOX015000.11.5	CRF01_AE (T/F)	0.02	0.10	100	0.03	0.15	100
BJOX010000.06.2	CRF01_AE (T/F)	>50	>50	18	11.20	>25	63
BJOX025000.01.1	CRF01_AE (T/F)	0.01	0.02	100	0.01	0.03	100
BJOX028000.10.3	CRF01_AE (T/F)	30.33	>50	57	0.04	0.18	98
X1193_c1	G	>50	>50	10	>25	>25	6
P0402_c2_11	G	0.03	0.08	100	0.03	0.07	100
X1254_c3	G	0.17	1.11	100	0.07	0.24	100
X2088_c9	G	>50	>50	12	>25	>25	9
X2131_C1_B5	G	>50	>50	21	>25	>25	25
P1981_C5_3	G	0.13	0.55	100	0.06	0.14	100
X1632_S2_B10	G	0.06	0.17	100	0.04	0.14	100
3016.v5.c45	D	0.04	0.12	100	0.10	0.33	100
A07412M1.vrc12	D	0.07	0.19	100	0.10	0.28	100
231965.c01	D	7.40	>50	70	0.26	0.57	100
231966.c02	D	0.17	0.59	99	0.12	0.42	100
6405.v4.c34	D	2.00	29.07	82	0.52	1.75	100
3817.v2.c59	CD	>50	>50	33	0.73	2.44	100
6480.v4.c25	CD	>50	>50	16	>25	>25	35
6952.v1.c20	CD	>50	>50	36	0.33	1.30	98
6811.v7.c18	CD	>50	>50	44	1.59	>25	76
89-F1_2_25	CD	>50	>50	15	>25	>25	19
3301.v1.c24	AC	>50	>50	21	>25	>25	22
6041.v3.c23	AC	0.20	0.76	96	0.43	1.31	97
6540.v4.c1	AC	7.07	>50	61	0.33	>25	70
6545.v4.c1	AC	5.04	>50	70	0.18	0.87	96
0815.v3.c3	ACD	>50	>50	21	>25	>25	45
3103.v3.c10	ACD	>50	>50	27	>25	>25	26
MuLV	Neg. Control	>50	>50	27	>25	>25	25

* (T/F): Transmitted / Founder Virus
MPI: Maximum Percent Inhibition

mAb titers (μg/ml)

- 0.001 - 0.01
- 0.01 - 0.1
- 0.1 - 1.0
- 1.0 - 20.0
- >20

Table S3. Cryo-EM data collection and refinement statistics. Related to Figure 3.

	SF12<br>B41 SOSIP.664 v4.2<br>10-1074
**PDB**	**6OKP**
**EMD**	**20100**
**Data collection and processing**	
Microscope	Titan Krios
Camera	Gatan K2 Summit
Magnification	130,000x
Voltage (kV)	300
Recording mode	counting
Dose rate (e^-/pixel/s)	4.8
Electron dose (e^-/Å^2)	40
Defocus range (µm)	1.2 - 3.0
Pixel size (Å)	1.09
Micrographs collected	2,732
Micrographs used	2,209
Total extracted particles	676,161
Refined particles	371,289
**Reconstruction**	
Final particles	301,920
Symmetry imposed	C1
Nominal Resolution (Å)	
FSC 0.5 (unmasked/masked)	4.15/3.67
FSC 0.143 (unmasked.masked)	3.71/3.28
Map sharpening *B* -factor	-110
**Refinement and Validation**	
Number of atoms	
Protein	20,391
Ligand	2,271
MapCC (global/local)	0.794/0.766
R.m.s. deviations	
Bond lengths (Å)	0.01
Bond angles (˚)	1.27
MolProbity score	1.78
Clashscore (all atom)	4.11
Poor rotamers (%)	0.48
Ramachandran plot	
Favored (%)	88.8
Allowed (%)	10.9
Disallowed (%)	0.31

Table S4. Crystallographic data collection and refinement statistics. Related to Figure 3.

PDB ID	SF12 Fab *(12-2, SSRL)* 6OKQ
**Data collection**[a]	
Space group	$P6_222$
Unit cell (Å)	223, 223, 288
α, β, γ (°)	90, 90, 120
Wavelength (Å)	1.0
Resolution (Å)	39.28-3.2 (3.26-3.19)
Unique Reflections	70,865 (4,473)
Completeness (%)	99.6 (98.7)
Redundancy	60.4 (56.8)
$CC_{1/2}$ (%)	90.3 (86.4)
<I/σI>	22.8 (1.4)
Mosaicity (˚)	0.07
R_{merge} (%)	21.6 (255)
R_{pim} (%)	4.0 (57.8)
Wilson *B*-factor	81.6
**Refinement and Validation**	
Resolution (Å)	39.2-3.2
Number of atoms	
Protein	9,980
Ligand	0
R_{work}/R_{free} (%)	27.3/29.9
R.m.s. deviations	
Bond lengths (Å)	0.01
Bond angles (˚)	1.4
MolProbity score	2.86
Clashscore (all atom)	16.4
Poor rotamers (%)	6.6
Ramachandran plot	
Favored (%)	92
Allowed (%)	6.4
Disallowed (%)	0.54
Average *B*-factor (Å)	158.3

[a]Numbers in parentheses correspond to the highest resolution shell

Table S5. Buried surface area calculations at the SF12-Env interface. Related to Figure 4.

Components	Total Area (Å^2)	Heavy Chain	Light Chain	Percentage (%)
N262-glycan	542	542	0	27.2
N295-glycan	470	470	0	23.6
N448-glycan	545	392	153	27.3
Peptide	436	436	0	21.9
**Total**	**1993**			100.0

Table S6. Effects of site-specific mutations in HIV-1 gp120 on SF12 and SF5 neutralization. Related to Figure 5 and 6.

	Titer in TZM.bl cells (ug/ml)									
	SF12		SF5		3BNC117		10-1074		PGDM1400	
Virus ID	IC_{50}	IC_{80}	IC_{50}	IC_{80}	IC_{50}	IC_{80}	IC_{50}	IC_{80}	IC_{50}	IC_{80}
BG505 WT	0.11	0.30	0.14	0.62	0.04	0.13	0.07	0.22	<0.01	0.03
BG505 WT	0.13	0.40	0.17	0.76	0.05	0.15	0.07	0.22	<0.01	0.03
BG505 P214Q	0.24	0.83	>25	>25	0.06	0.20	0.08	0.26	<0.01	0.03
BG505 P214I	0.27	0.87	>25	>25	0.05	0.19	0.07	0.24	<0.01	0.03
BG505 N262S	>25	>25	>25	>25	0.02	0.09	0.05	0.15	<0.01	0.02
BG505 N262W	>25	>25	>25	>25	0.03	0.27	0.10	0.28	<0.01	0.08
BG505 Q293E	0.25	0.86	0.17	0.57	0.03	0.14	0.09	0.29	<0.01	0.03
BG505 Q293K	0.06	0.21	2.23	>25	0.04	0.15	0.08	0.25	<0.01	0.03
BG505 Q293R	0.06	0.18	0.30	6.24	0.05	0.16	0.06	0.23	<0.01	0.03
BG505 N295T	0.03	0.10	0.04	0.18	0.04	0.13	0.03	0.11	<0.01	0.03
BG505 N295V	0.04	0.12	0.07	0.44	0.04	0.15	0.04	0.13	<0.01	0.03
BG505 R444T	0.27	0.90	>25	>25	0.04	0.13	0.08	0.25	<0.01	0.03
BG505 N448K	>25	>25	>25	>25	0.05	0.17	0.06	0.21	<0.01	0.03
BG505 N448S	>25	>25	>25	>25	0.04	0.12	0.06	0.19	<0.01	0.02

	Titer in TZM.bl cells (ug/ml)									
	SF12		SF5		3BNC117		10-1074		PGDM1400	
Virus ID	IC_{50}	IC_{80}	IC_{50}	IC_{80}	IC_{50}	IC_{80}	IC_{50}	IC_{80}	IC_{50}	IC_{80}
YU2 WT	0.52	1.64	0.70	2.29	<0.01	0.06	0.15	0.58	0.27	0.89
YU2 WT	0.61	1.81	0.89	2.67	0.01	0.09	0.17	0.59	0.23	0.86
YU2 P214Q	0.44	1.52	8.72	>25	<0.01	0.07	0.07	0.32	0.21	0.85
YU2 P214I	0.23	0.85	6.04	>25	<0.01	0.03	0.02	0.15	0.12	0.65
YU2 N262S	>25	>25	>25	>25	<0.01	0.02	<0.01	0.08	0.15	0.60
YU2 N262W	>25	>25	>25	>25	<0.01	<0.01	<0.01	0.09	0.19	1.47
YU2 S291P	0.58	1.72	0.71	2.15	0.02	0.07	0.14	0.50	0.31	1.13
YU2 S291T	0.54	1.65	0.86	2.30	0.02	0.08	0.18	0.60	0.30	0.98
YU2 V293E	0.86	2.46	0.62	2.02	0.01	0.06	0.11	0.42	0.28	1.09
YU2 V293K	0.32	0.90	4.73	>25	0.01	0.06	0.13	0.52	0.24	0.87
YU2 V293R	0.22	0.71	4.83	>25	<0.01	0.05	0.12	0.50	0.24	0.92
YU2 N295T	0.07	0.24	0.06	0.20	0.02	0.08	0.07	0.28	0.27	1.01
YU2 N295V	0.04	0.20	0.03	0.17	0.01	0.06	0.05	0.32	0.24	0.79
YU2 R444T	1.06	3.36	3.13	16.23	0.01	0.06	0.11	0.51	0.22	0.91
YU2 N448K	>25	>25	>25	>25	0.01	0.08	0.16	0.56	0.32	1.15
YU2 N448S	>25	>25	>25	>25	<0.01	0.07	0.15	0.55	0.26	1.14

Table S7. Computational analysis of neutralization data for silent face antibodies. Related to Figure 5 and 6.

	SF12		SF5		VRC-PG05	
	IC_{50} (μg/ml)[a,b]	Coverage (%)	IC_{50} (μg/ml)	Coverage (%)	IC_{50} (μg/ml)	Coverage (%)
**Viruses**						
Cross-clade panel	0.20 (n=119)	62.2%	0.25 (n=119)	58.0%	0.84 (n=220)	27.3%
Clade AE	0.09 (n=15)	100.0%	0.05 (n=15)	93.3%	1.56 (n=35)	57.1%
Clade B	0.19 (n=21)	100.0%	0.2 (n=21)	85.7%	0.79 (n=41)	31.7%
Clade C	0.69 (n=31)	35.5%	0.49 (n=31)	33.3%	0.55 (n=71)	22.5%
**Glycans**						
N448+	0.21 (n=98)	75.5%	0.23 (n=98)	69.4%	0.81 (n=168)	32.1%
N448-	>50 (21)	0.0%	>50 (21)	0.0%	>50 (24)	0.0%
N295+	0.19 (n=56)	73.2%	0.29 (n=56)	64.3%	2.20 (n=94)	26.6%
N295-	0.25 (n=63)	51.6%	0.21 (n=63)	51.6%	0.34 (n=98)	29.6%
N442+	0.29 (n=41)	41.5%	0.3 (n=41)	39.0%	0.84 (n=60)	21.7%
N442-	0.19 (n=78)	72.4%	0.24 (n=78)	67.5%	0.8 (n=132)	31.1%
**Peptide**						
E293+	0.2 (n=54)	64.8%	0.16 (n=54)	63.3%	0.82 (n=95)	51.6%
E293-	0.22 (n=65)	59.4%	0.38 (n=65)	53.8%	0.66 (n=97)	5.2%
S291+	0.2 (n=74)	67.6%	0.2 (n=74)	64.9%	0.7 (n=118)	39.0%
S291-	0.25 (n=45)	52.3%	0.43 (n=45)	45.5%	1.9 (n=74)	10.8%
T444+	0.38 (n=46)	37.0%	0.29 (n=46)	34.8%	0.35 (n=69)	21.7%
T444-	0.18 (n=73)	77.8%	0.24 (n=73)	72.2%	1.11 (n=123)	31.7%

[a]Mean IC_{50} of neutralized strains

[b]Number in paranthesis represents number of strains that fit the filtered criteria
