# Supplementary material for: Broad and Potent Neutralizing Antibodies Recognize the Silent Face of the HIV Envelope
Source: Immunity. 2019 Jun 18;50(6):1513–1529.e9. doi: 10.1016/j.immuni.2019.04.014 (PMC6591006; doi:10.1016/j.immuni.2019.04.014)
Supplement: Document S2. Article plus Supplemental Information [file mmc2.pdf]

Immunity

Article

Broad and Potent Neutralizing Antibodies Recognize the Silent Face of the HIV Envelope

Graphical Abstract

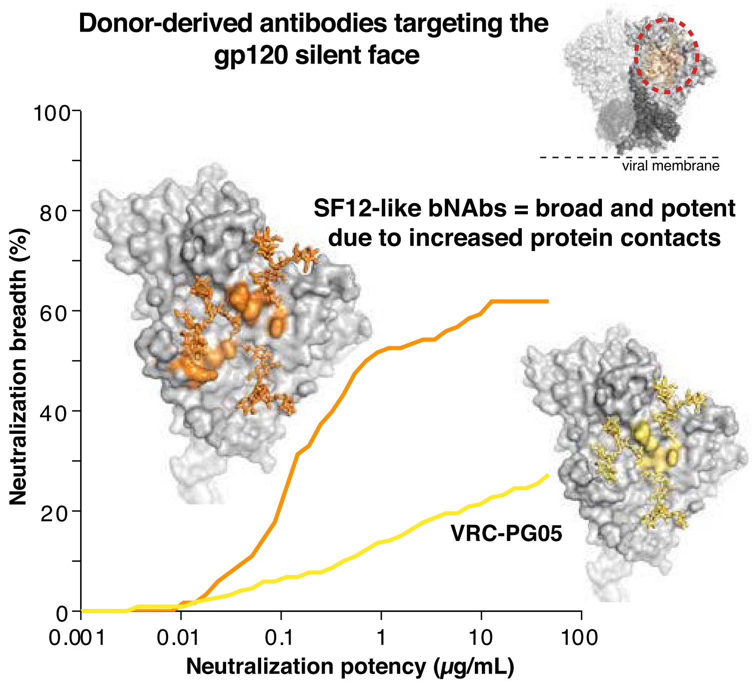

Authors

Till Schoofs, Christopher O. Barnes, Nina Suh-Toma, ..., Florian Klein, Michel C. Nussenzweig, Pamela J. Bjorkman

Correspondence

nussen@rockefeller.edu (M.C.N.), bjorkman@caltech.edu (P.J.B.)

In Brief

VRC-PG05 was the only donor-derived antibody against the silentface (SF) of HIV-1 envelope described to date. Schoofs et al. identify the antibody SF12 and its relatives, which recognize the center of the SF with a different angle and more extensive protein recognition than VRC-PG05, thereby achieving substantial neutralizing ability and potential for clinical use.

Highlights

- Silentface (SF) bNAbs can reach substantial neutralization breadth and potency
- Defined structure of a SF bNAb bound to an Env trimer
- Binding of SF bNAb resulted in trimer asymmetry in the V3 region
- SF antibodies have *in vivo* activity and potential for clinical use

Schoofs et al., 2019, Immunity *50*, 1513–1529
June 18, 2019 © 2019 The Authors. Published by Elsevier Inc.
https://doi.org/10.1016/j.immuni.2019.04.014

Immunity
Article

Broad and Potent Neutralizing Antibodies Recognize the Silent Face of the HIV Envelope

Till Schoofs,[1,2,3,13,14] Christopher O. Barnes,[4,13] Nina Suh-Toma,[4,5] Jovana Golijanin,[1] Philipp Schommers,[2,3] Henning Gruell,[2,3] Anthony P. West, Jr.,[4] Franziska Bach,[2] Yu Erica Lee,[4] Lilian Nogueira,[1] Ivelin S. Georgiev,[6,7] Robert T. Bailer,[8] Julie Czartoski,[9] John R. Mascola,[8] Michael S. Seaman,[10] M. Juliana McElrath,[9] Nicole A. Doria-Rose,[8] Florian Klein,[2,3,11] Michel C. Nussenzweig,[1,12,*] and Pamela J. Bjorkman[4,15,*]

[1]Laboratory of Molecular Immunology, The Rockefeller University, New York, NY 10065, USA
[2]Laboratory of Experimental Immunology, Institute of Virology, Faculty of Medicine and University Hospital of Cologne, University of Cologne, 50931 Cologne, Germany
[3]German Center for Infection Research, partner site Bonn-Cologne, 50931 Cologne, Germany
[4]Division of Biology and Biological Engineering, California Institute of Technology, Pasadena, CA 91125, USA
[5]Westridge High School, 324 Madeline Drive, Pasadena, CA 91105, USA
[6]Vanderbilt Vaccine Center, Department of Pathology, Microbiology and Immunology, Vanderbilt University Medical Center, Nashville, TN 37232, USA
[7]Department of Electrical Engineering and Computer Science, Vanderbilt University, Nashville, TN 37232, USA
[8]Vaccine Research Center, National Institute of Allergy and Infectious Diseases, NIH, Bethesda, MD 20892, USA
[9]Vaccine and Infectious Disease Division, Fred Hutchinson Cancer Research Center, Seattle, WA 98109, USA
[10]Center for Virology and Vaccine Research, Beth Israel Deaconess Medical Center, Harvard Medical School, Boston, MA 02215, USA
[11]Center for Molecular Medicine Cologne (CMMC), University of Cologne, 50931 Cologne, Germany
[12]Howard Hughes Medical Institute, The Rockefeller University, New York, NY 10065, USA
[13]These authors contributed equally
[14]Present address: GSK Vaccines, 1300 Wavre, Belgium
[15]Lead Contact
*Correspondence: nussen@rockefeller.edu (M.C.N.), bjorkman@caltech.edu (P.J.B.)
https://doi.org/10.1016/j.immuni.2019.04.014

SUMMARY

Broadly neutralizing antibodies (bNAbs) against HIV-1 envelope (Env) inform vaccine design and are potential therapeutic agents. We identified SF12 and related bNAbs with up to 62% neutralization breadth from an HIV-infected donor. SF12 recognized a glycan-dominated epitope on Env's silent face and was potent against clade AE viruses, which are poorly covered by V3-glycan bNAbs. A 3.3Å cryo-EM structure of a SF12-Env trimer complex showed additional contacts to Env protein residues by SF12 compared with VRC-PG05, the only other known donor-derived silent-face antibody, explaining SF12's increased neutralization breadth, potency, and resistance to Env mutation routes. Asymmetric binding of SF12 was associated with distinct N-glycan conformations across Env protomers, demonstrating intra-Env glycan heterogeneity. Administrating SF12 to HIV-1-infected humanized mice suppressed viremia and selected for viruses lacking the $N448_{gp120}$ glycan. Effective bNAbs can therefore be raised against HIV-1 Env's silent face, suggesting their potential for HIV-1 prevention, therapy, and vaccine development.

INTRODUCTION

Neutralizing antibodies (NAbs) play a key role in antiviral immunity and are the correlate of protection of most available vaccines (Burton, 2002; Plotkin, 2010). The HIV-1 envelope glycoprotein (Env) is the only potential target for NAbs on the surface of the virus (Burton and Hangartner, 2016; Kwong and Mascola, 2018; Wibmer et al., 2015). Env is a trimeric spike composed of gp120/gp41 heterodimers that has evolved a plethora of immune escape mechanisms to evade antibody recognition. These include instability of the trimer, sparsity of spikes on the virion surface, high sequence divergence across strains, and epitope masking through its extensive glycan shield (Burton and Hangartner, 2016; Haynes, 2015; Klein and Bjorkman, 2010; Kwong and Mascola, 2018).

Consequently, effective humoral responses to HIV-1 typically only emerge several years after infection and only in a subset of HIV-1-infected individuals (Gray et al., 2011; Landais et al., 2016; Mikell et al., 2011; Rusert et al., 2016; Tomaras et al., 2011). Although ~50% of chronically HIV-1-infected individuals develop some degree of cross-clade serum neutralization, only a small fraction of individuals mounts outstandingly broad and potent antibody responses against the virus (Doria-Rose et al., 2010; Hraber et al., 2014; Landais et al., 2016; Rusert et al., 2016; Sather et al., 2009; Simek et al., 2009). The development and use of single B cell antibody cloning revealed that this activity can usually be attributed to one or a combination of broadly neutralizing antibodies (bNAbs) that target HIV-1 Env (Scheid

This is an open access article under the CC BY license (http://creativecommons.org/licenses/by/4.0/).

et al., 2009a, 2009b, 2011; Walker et al., 2009, 2010; Wu et al., 2010).

NAbs are proposed to interfere with viral entry in a variety of ways, including blocking receptor engagement, preventing membrane fusion, and enhancing decay of Env spikes (Burton and Hangartner, 2016). Pre-clinical and recent human studies have highlighted the potential of bNAbs for HIV-1 therapy and prevention (Bar et al., 2016; Bar-On et al., 2018; Barouch et al., 2013; Caskey et al., 2015, 2017; Gautam et al., 2016; Julg et al., 2017; Lynch et al., 2015; Mendoza et al., 2018; Scheid et al., 2016; Shingai et al., 2013). Moreover, structural insights into mechanisms of bNAb binding have been key to designing novel immunogens and strategies for vaccination (Escolano et al., 2017; Jardine et al., 2013; Kwong and Mascola, 2018; McGuire et al., 2013; Sanders et al., 2013; Ward and Wilson, 2017). However, there remain a number of challenges to the elicitation and clinical use of bNAbs. For vaccine efforts, these include unusual structural features of bNAbs such as large insertions/deletions, and/or unusual complementarity determining region (CDR) lengths as well as extensive somatic hypermutation (SHM), all of which are rare features in the human repertoire (Burton and Hangartner, 2016; Haynes and Burton, 2017; Sok and Burton, 2018). For clinical use of bNAbs, viral coverage gaps, manufacturability, and pre-existing bNAb resistance represent potential problems (Escolano et al., 2017; Gruell and Klein, 2018; Sok and Burton, 2018). Thus, there is a continuing need to identify bNAbs that may be more readily elicited by vaccination and that are suitable for clinical use.

Although many bNAbs have been characterized, their targets, or "sites of vulnerability", on the HIV-1 Env spike appear to be limited (Burton and Hangartner, 2016; Kwong and Mascola, 2018; Sok and Burton, 2018; Ward and Wilson, 2017; West et al., 2014; Wibmer et al., 2015). Numerous monoclonal antibodies recognize the CD4-binding site, the V3-glycan patch, the V2-apex, the membrane proximal external region (MPER), and several epitopes encompassing the gp120-gp41 interface (Burton and Hangartner, 2016; Kwong and Mascola, 2018; Sok and Burton, 2018; Ward and Wilson, 2017; Wibmer et al., 2015). In contrast, VRC-PG05 is the only donor-derived antibody isolated to date that binds to the highly glycosylated "silent face" of gp120 (Zhou et al., 2018). However, VRC-PG05 neutralized only 27% of tested HIV-1 strains and had a relatively high mean IC_{50} of 0.8 μg/mL, leaving uncertain the potential usefulness of this epitope for vaccine design, therapy, or prevention.

Here, we describe silent face (SF) bNAbs targeting a VRC-PG05-related epitope that cover up to 62% of evaluated strains with a mean IC_{50} of 0.20 μg/mL. To characterize the binding mechanism of the new antibodies, we determined the 3.1 Å crystal of the unbound SF12 Fab and a 3.3 Å cryo-EM structure SF12 Fab bound to the clade B B41 Env trimer. We found that SF12 binds the center of the Env silent face with a different orientation and set of contacts than VRC-PG05. The overall breadth and potency achieved by SF12 suggests that the silent face is an additional target for vaccine design and that antibodies to this site may be clinically useful as a complement to other available bNAbs.

RESULTS

Isolation of an Antibody Family from Donor 27845 by B Cell Culture and BG505 Sorting

Donor 27845 was diagnosed with HIV-1 in 1985 and followed in a cohort of long-term non-progressors at the Fred Hutchinson Cancer Research Center from 1998–2006. Apart from an interventional study during which the subject started and stopped anti-retroviral therapy (ART) at set intervals from 1998–2001, the subject has been off ART (Figure 1A). The individual's purified immunoglobulin G (IgG) isolated from a 2005 time point was evaluated for neutralization against a 12-virus panel representative of the global epidemic (deCamp et al., 2014) (Figure 1B) and found to be both broad and potent with a coverage of 92% and an average median inhibitory concentration (IC_{50}) of 92.3 μg/mL (Figure 1B). To inform potential antibody isolation strategies, neutralization fingerprinting of the subject's IgG was performed, but the results were inconclusive due to borderline prediction confidence scores (Doria-Rose et al., 2017) (Figure 1B).

Based on the fingerprinting results, we employed an unbiased B cell microculture approach for antibody cloning (Doria-Rose et al., 2015; Huang et al., 2013). From a starting number of 4.4×10^4 memory B cells, we identified seven B cells, six of which were members of a single clone, that showed potent anti-HIV-1 neutralizing activity against two indicator strains. Subsequent single B cell sorting using fluorescently labeled BG505.SOSIP.664 native-like Env trimers (Sanders et al., 2013) yielded two additional members of this antibody family, one of which was identical to an antibody obtained in the B cell culture. The members of the clone utilized V_H4-59*01 and V_K3-20*01 heavy and light chain variable gene segments and included CDRH3s and CDRL3s of 23 and 6 amino acids, respectively (Table S1). V_H gene segment mutation frequencies ranged from 17%–25% of nucleotides (21%–39% amino acids), and V_K gene segment mutation frequencies ranged from 15%–21% of nucleotides (20%–29% amino acids), intermediate rates of SHM for HIV-1 bNAbs (Table S1). Based on heavy chain sequences, the family segregated into three phylogenetic branches (Figure 1C), with the SF5/SF12 branch showing a three-nucleotide CDRH2 insertion.

When tested on two representative panels of 20 (f61 panel) and 12 (global panel) viruses (deCamp et al., 2014; Doria-Rose et al., 2017), members of the V_H4-59 clone showed diverse levels of activity and breadth (Figure 1D). Two closely related members of the V_H4-59 clone that were the most active, SF5 and SF12, were then evaluated against a 119-virus panel representative of all major circulating HIV-1 clades (Figure 1E; Table S2) (Freund et al., 2017; Mouquet et al., 2012). SF5 and SF12 neutralized 58% and 62% of viruses in this larger panel, with geometric mean IC_{50s} of 0.25 and 0.20 μg/mL, respectively. Notably, SF12 neutralized all of 18 tested clade AE viruses across the three panels and showed a pattern of neutralizing activity that differed from previously described bNAbs (Figure 1F). Overall, the antibody clone recapitulated the majority of the polyclonal IgG neutralization activity, with the potency correlation between isolated monoclonal antibodies (mAbs) and donor 27845's IgG resembling those of other elite neutralizers from whom we previously isolated bNAbs (Freund et al., 2017; Scheid et al., 2011).

A

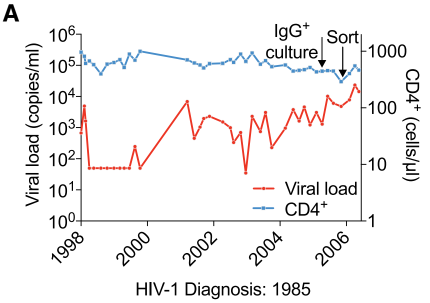

B

Global panel		
Virus ID	Clade	IgG
398-F1_F6_20	A	49.2
CNE8	AE	72.7
CNE55	AE	54.3
246-F3_C10_2	AC	104.3
X2278_C2_B6	B	66.7
TRO.11	B	83.9
CH119.10	BC	186.4
BJOX002000.03.2	BC	>500
25710-2.43	C	95.0
Ce1176_A3	C	88.4
Ce703010217_B6	C	304.5
X1632_S2_B10	G	77.1

IgG (μg/ml)	mAb titers (μg/ml)
0 - 25	0.001 - 0.01
25 - 100	0.01 - 0.1
100 - 400	0.1 - 1.0
400 - 500	1.0 - 20.0
> 500	>20

f61 panel		
Virus ID	Clade	IgG
KER2008.12	A	39.5
Q259.d2.17	A	279.6
Q461.e2	AD	>500
620345.c1	AE	61.2
CNE5	AE	92.0
CNE55	AE	53.6
M02138	AE	80.3
TH976.17	AE	165.5
242-14	AG	231.0
7165.18	B	88.9
YU2.DG	B	205.5
CH038.12	BC	175.4
CH070.1	BC	>500
0013095-2.11	C	>500
001428-2.42	C	61.6
26191-2.48	C	>500
3168.v4.c10	C	169.5
ZM135M.PL10a	C	391.4
231965.c1	D	113.6
247-23	D	67.2

Neutralization fingerprinting	
Specificity	27845
VRC01-like	0.11
b12-like	
HJ16-like	
8ANC195-like	
PG9-like	0.15
PGT128-like	0.10
2F5-like	0.61
10E8-like	
35O22-like	
PGT151-like	0.03
Residual Score	-0.20
Median of scores	0.02

C

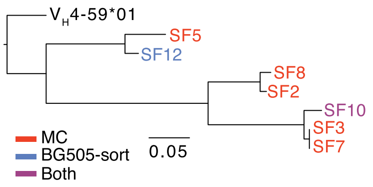

D

f61 fingerprinting panel								
Virus ID	Clade	SF5	SF12	SF2	SF8	SF3	SF7	SF10
KER2008.12	A	0.11	0.10	>20	>20	>20	>20	18.15
Q259.d2.17	A	1.49	0.24	>20	>20	>20	>20	0.27
Q461.e2	AD	>20	>20	>20	>20	>20	>20	19.88
620345.c1	AE	0.02	0.03	>20	>20	0.58	0.34	0.01
CNE5	AE	0.03	0.32	>20	>20	>20	>20	0.12
CNE55	AE	0.04	2.77	>20	>20	>20	>20	>20
M02138	AE	0.06	0.10	>20	>20	3.84	5.20	0.01
TH976.17	AE	0.17	0.08	>20	>20	>20	>20	>20
242-14	AG	>20	>20	>20	>20	>20	>20	>20
7165.18	B	0.14	0.10	0.08	0.07	0.05	0.04	0.04
YU2.DG	B	0.51	0.28	0.12	0.10	0.14	0.11	0.10
CH038.12	BC	>20	0.32	0.29	0.46	>20	>20	>20
CH070.1	BC	>20	>20	>20	>20	>20	>20	>20
0013095-2.11	C	>20	>20	>20	>20	>20	>20	>20
001428-2.42	C	0.06	0.10	>20	>20	>20	>20	6.98
26191-2.48	C	>20	>20	>20	>20	>20	>20	>20
3168.v4.c10	C	>20	>20	>20	>20	>20	>20	>20
ZM135M.PL10a	C	>20	>20	>20	>20	>20	>20	>20
231965.c1	D	4.54	0.18	0.10	0.08	0.04	0.04	0.04
247-23	D	0.04	0.04	19.22	>20	>20	>20	0.02

Global panel								
Virus ID	Clade	SF5	SF12	SF2	SF8	SF3	SF7	SF10
398-F1_F6_20	A	>20	2.13	0.04	0.04	<0.01	<0.01	0.01
TRO.11	B	0.46	0.31	0.10	0.07	0.03	0.02	0.06
X2278_C2_B6	B	0.09	0.08	0.62	0.22	3.62	2.02	0.02
25710-2.43	AC	>20	5.07	>20	>20	>20	>20	>20
Ce1176_A3	C	>20	>50	>20	>20	>20	>20	>20
Ce703010217_B6	C	>20	>50	>20	>20	>20	>20	>20
246-F3_C10_2	AC	0.12	0.09	>20	>20	>20	>20	1.26
CNE8	AE	0.12	0.04	>20	>20	1.41	2.26	0.04
CNE55	AE	0.04	4.92	>20	>20	>20	>20	>20
BJOX002000.03.2	BC	>20	>50	>20	>20	>20	>20	>20
CH119.10	BC	>20	37.21	>20	>20	>20	>20	>20
X1632_S2_B10	G	0.05	0.03	>20	>20	>20	>20	>20

E

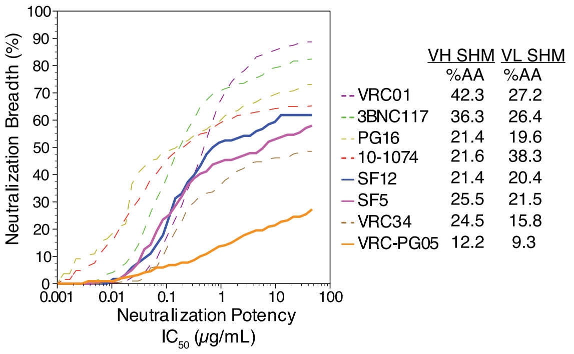

F

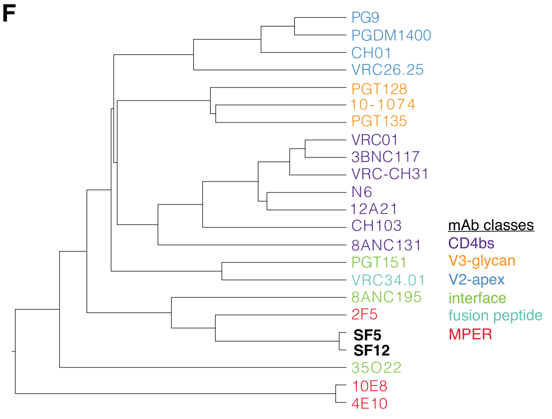

Figure 1. Isolation of Antibody Family from Donor 27845 by B Cell Culture and BG505 Sorting

(A) Viral load and $CD4^+$ T cell counts of HIV-1-infected subject 27845 over time. Arrows indicate time points of B cell microculture and BG505.SOSIP.664 bait-sorting.

(B) Neutralization data of donor 27845's serum IgG in 2005 against a 12-virus cross-clade panel (global) and a 20-virus fingerprinting panel (f61). Shown are median inhibitory concentrations (IC_{50}) in μg/mL. On the right, fingerprinting analysis of f61 serum neutralization. Neutralization testing performed in duplicates, average shown.

(C) Maximum-likelihood phylogenetic tree of heavy chain sequences of newly isolated antibody family. MC = Antibodies isolated by B cell microculture, BG505-sort = antibodies isolated by bait-sorting, Both = antibody found both by microculture and bait-sorting.

(D) Neutralization of isolated antibody family members (IC_{50}) against global and f61 virus panels. Legend as in (B). Neutralization testing performed in duplicates, average shown.

(E) Neutralization coverage and potency of SF5 and SF12 on a 119-virus cross clade panel. Neutralization testing performed in duplicates, average shown.

(F) Neutralization fingerprinting of SF5 and SF12 in comparison to other known anti-HIV-1 bNAbs.

See also Figure S1 and Tables S1 and S2.

We next evaluated potential autoreactivity and polyreactivity of SF12 and SF5 using HEp-2 staining (Haynes et al., 2005) and a baculovirus-based polyreactivity assay (Hötzel et al., 2012), respectively (Figure S1). In contrast to bNAbs with known autoreactive and polyreactive properties such as 2F5 and 4E10 (Haynes et al., 2005), we found minimal to no autoreactivity or

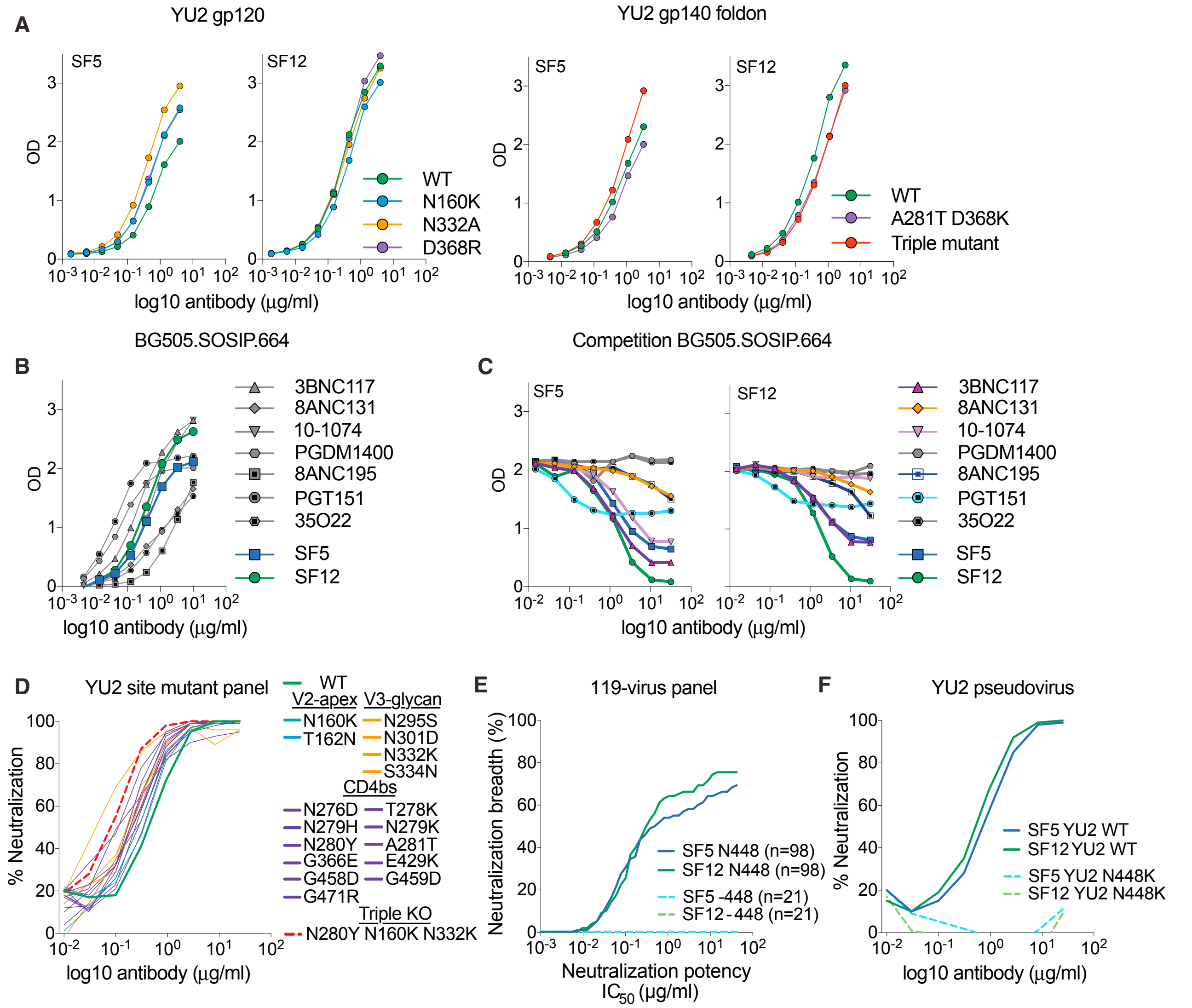

Figure 2. Antibodies SF5 and SF12 Bind a Distinct Epitope on the gp120 Portion of Env

(A) ELISA of SF5 and SF12 against a gp120 monomer and a gp140 foldon trimer derived from HIV-1 strain YU2. Wild-type proteins and various site mutants of the proteins in common bNAb epitopes (CD4-binding site, V3-glycan, Apex) were tested. Triple mutant = N160K, A281T + D368K, N332K. Data representative of 3 repeat assays.

(B) ELISA of SF5 and SF12 as well as reference bNAbs targeting 6 known epitopes against the BG505.SOSIP.664 trimer. Data representative of 3 repeat assays.

(C) Competition ELISA with reference bNAbs targeting 6 known epitopes to evaluate interference with SF5 and SF12 binding to the BG505.SOSIP.664 trimer. Competing antibodies were added in a dilution series starting at 32 μg/mL. SF5 and SF12 were added at a constant concentration of 0.5 μg/mL. Data representative of 3 repeat assays.

(D) Neutralization testing of SF12 against a panel of YU2 site mutants covering major epitopes on the HIV-1 spike. Neutralization testing performed in duplicates, average curves shown.

(E) Computational analysis of 119-virus cross clade panel neutralization.

(F) Neutralization testing of SF5 and SF12 against an HIV-1 pseudovirus based on strain YU2 carrying a mutation at the PNGS $N448_{gp120}$. Testing done in duplicates, average shown.

polyreactivity for SF5 and SF12 (Figures S1A and S1B). In addition, the pharmacokinetics of SF12 in mice were similar to those of 3BNC117, a bNAb that exhibits a typical IgG1 half-life in macaques (Gautam et al., 2016) and humans (Caskey et al., 2015) (Figure S1C). We conclude that the SF antibody family achieves substantial anti-HIV-1 neutralization with an intermediate degree of somatic hypermutation and no evidence for autoreactivity.

Antibodies SF5 and SF12 Bind a Distinct Epitope on the gp120 Portion of Env

To map the epitope recognized by SF5 and SF12, we performed ELISAs using HIV-1 Env proteins. Both antibodies bound to monomeric YU2 gp120, indicating that a portion of the epitope is contained within the gp120 subunit of Env (Figure 2A). We subsequently evaluated ELISA binding to site-directed mutants in

monomeric YU2 gp120 and an uncleaved YU2 gp140 foldon trimer (Yang et al., 2000) that define common epitopes. Mutation of the CD4-binding site (D368R/D368K$_{gp120}$, A281T$_{gp120}$) (Dosenovic et al., 2015; Horwitz et al., 2013; Olshevsky et al., 1990), the V3 glycan patch (N332A/N332K$_{gp120}$) (Horwitz et al., 2013; Mouquet et al., 2012), and the V2 apex epitope (N160K$_{gp120}$) (Walker et al., 2009), alone or in combination, did not abrogate SF5 or SF12 binding (Figure 2A). SF5 and SF12 also bound to a cleaved soluble native-like BG505.SOSIP.664 trimer (Sanders et al., 2013) (Figure 2B). Taken together, these results suggested that SF5 and SF12 bind an epitope that is present on gp120 monomers and both cleaved and uncleaved Env trimers.

We performed competition ELISAs to assess binding to the BG505.SOSIP.664 trimer using antibodies targeting the CD4-binding site (3BNC117, 8ANC131), the V3 glycan patch (10-1074), the V2-apex (PGDM1400) and the gp120-gp41 interface (8ANC195, PGT151 and 35O22). Both SF5 and SF12 competed strongly with themselves and each other (Figure 2C). 3BNC117, a CD4-binding site antibody that bridges adjacent protomers within a trimer and has a broad contact surface with gp120 (Lee et al., 2017), showed competition with both SF5 and SF12. Incomplete competition was also observed for the CD4-binding site antibody 8ANC131 and for the gp120-gp41 interface antibodies 8ANC195 and PGT151. Moreover, the V3-glycan targeting antibody 10-1074 competed strongly with SF5 but not with SF12 (Figure 2C). We also assessed the neutralizing activity of SF12 on a YU2 pseudovirus mutant panel comprising a number of mutations that impair the activity of CD4-binding site, V3-glycan and V2-apex antibodies using a TZM.bl-based *in vitro* neutralization assay. SF12 neutralizing activity was insensitive to the mutations, including a triple mutant carrying mutations in all three epitopes (N280Y$_{gp120}$, N160K$_{gp120}$, N332K$_{gp120}$) (Figure 2D). These data indicate that SF5 and SF12 bind a distinct epitope near the epitopes for CD4-binding site bNAbs and gp120-gp41 interface bNAbs 8ANC195 and PGT151.

Computational analysis (West et al., 2013) of available neutralization data suggested that SF5/SF12 depend on the presence of a glycan at N448$_{gp120}$ (Figure 2E). To verify that the neutralizing activity of SF5 and SF12 depended on this potential N-linked glycosylation site (PNGS), we showed that these antibodies failed to neutralize a mutant HIV$_{YU2}$ pseudovirus lacking the N448$_{gp120}$ glycan (Figure 2F). The PNGS at position 448$_{gp120}$ is at the center of one of the most highly glycosylated parts of the HIV-1 trimer, also known as the silent face (Wyatt et al., 1998). Although comparisons of synonymous versus non-synonymous mutations suggested that the silent face is under immunologic pressure (Stewart et al., 2001), antibodies that bind to the center of this region have been difficult to isolate. Indeed, VRC-PG05 represented an, until now, unique example of a host-derived bNAb that specifically targets the center of the silent face with a focus on the glycan site at N448$_{gp120}$ (Zhou et al., 2018). The discovery and characterization of SF12 and related silent face bNAbs shows that this epitope can be targeted by antibodies with greater breadth and potency than VRC-PG05.

Structure of the Natively Glycosylated SF12-Env Complex

We determined a 3.1 Å crystal structure of the SF12 Fab and a 3.3 Å cryo-EM structure of a natively glycosylated clade B B41 SOSIP.664 trimer in complex with the SF12 Fab and a Fab from the V3/glycan patch bNAb 10-1074 (Figures 3A and 3B). Although 10-1074 Fab normally binds with a 3:1 Fab:Env trimer stoichiometry (Gristick et al., 2016), EM class averages showed either three or two SF12 Fabs bound to the Env trimer and only one 10-1074 Fab (Figures S2 and S3). Like VRC-PG05, for which a crystal structure was solved in complex with a monomeric gp120 core (Zhou et al., 2018), the SF12-trimer complex reveals recognition of an epitope focused on the N262$_{gp120}$, N295$_{gp120}$, and N448$_{gp120}$ glycans on the silent face of Env, rationalizing our binding and *in vitro* neutralization results (Figures 2A–2F). Superimposition of the free and Env-bound SF12 Fab structures showed only minor conformational changes resulting from Env glycan interactions with the SF12 Fab in the Env-bound structure, as evidenced by the 1.1 Å root-mean-square deviation (RMSD) relating 245 Cα atoms in the V_H and V_L domains of the free and bound Fabs (Figure 3C).

We found three distinct differences between the structures of a VRC-PG05 Fab-monomeric CNE55 gp120 core and the SF12-Env trimer complexes (Zhou et al., 2018). First, the longer CDRH3 of SF12 was extended in a different conformation from that of the shorter CDRH3 in VRC-PG05, resulting in a RMSD of 3.1 Å across 130 Cα atoms when superposing the V_H domains from the Fab-bound structures (Figure 3C). Second, the SF12 CDRL1 and CDRL3 loops adopted conformations different from those of their VRC-PG05 counterparts (Figure S4). In the SF12 Fab, the CDRH3 and CDRL loops form a groove at the Fab-antigen interface that accommodates the N448$_{gp120}$ glycan, which contrasts with the wedge between the VRC-PG05 CDRH3 and CDRL1 loops that penetrates through Env glycans (Figures 3D and 3E). Third, the orientation of the SF12 Fab differed from that of VRC-PG05 Fab, with the SF12 Fab exhibiting an almost perpendicular binding angle to the silent face epitope compared with the VRC-PG05 orientation (Figures 3F and S4). To evaluate this difference, we calculated the rotation and translation of the V_H-V_L domains of the Fab portions of the SF12-Env trimer and VRC-PG05-gp120 complex structures, finding that the orientations of the SF12 and VRC-PG05 V_H-V_L domains differed, with the axis of the SF12 Fab at a steeper angle (by $\sim$71°) to the silent face epitope than the axis of VRC-PG05. Despite differences in approach angles to the silent face epitope, SF12 and VRC-PG05 shared a common mode of interaction with the N448$_{gp120}$ glycan, mediated in each case by their CDRH3 loops (Figure 3G). We conclude that SF12 binds a VRC-PG05-related epitope with a different angle of approach and an altered mode of recognition from VRC-PG05.

SF12 Recognizes a Mostly Glycan-Focused Epitope on HIV-1 Env

In contrast to the non-natively glycosylated monomeric CNE55 gp120 core that was complexed with VRC-PG05 Fab (Zhou et al., 2018), the relatively high resolution cryo-EM structure of the natively glycosylated SF12-Env trimer complex allowed modeling of N-linked glycans in the B41 Env trimer (Figure 3). Given the asymmetric Fab binding in our complex, we characterized the SF12 epitope and paratope using a gp140 protomer in which the SF12, but not the 10-1074, Fab was bound (Figures 4A–4C). Consistent with differing binding angles and CDR loop conformations, SF12's footprint on Env differed from that of

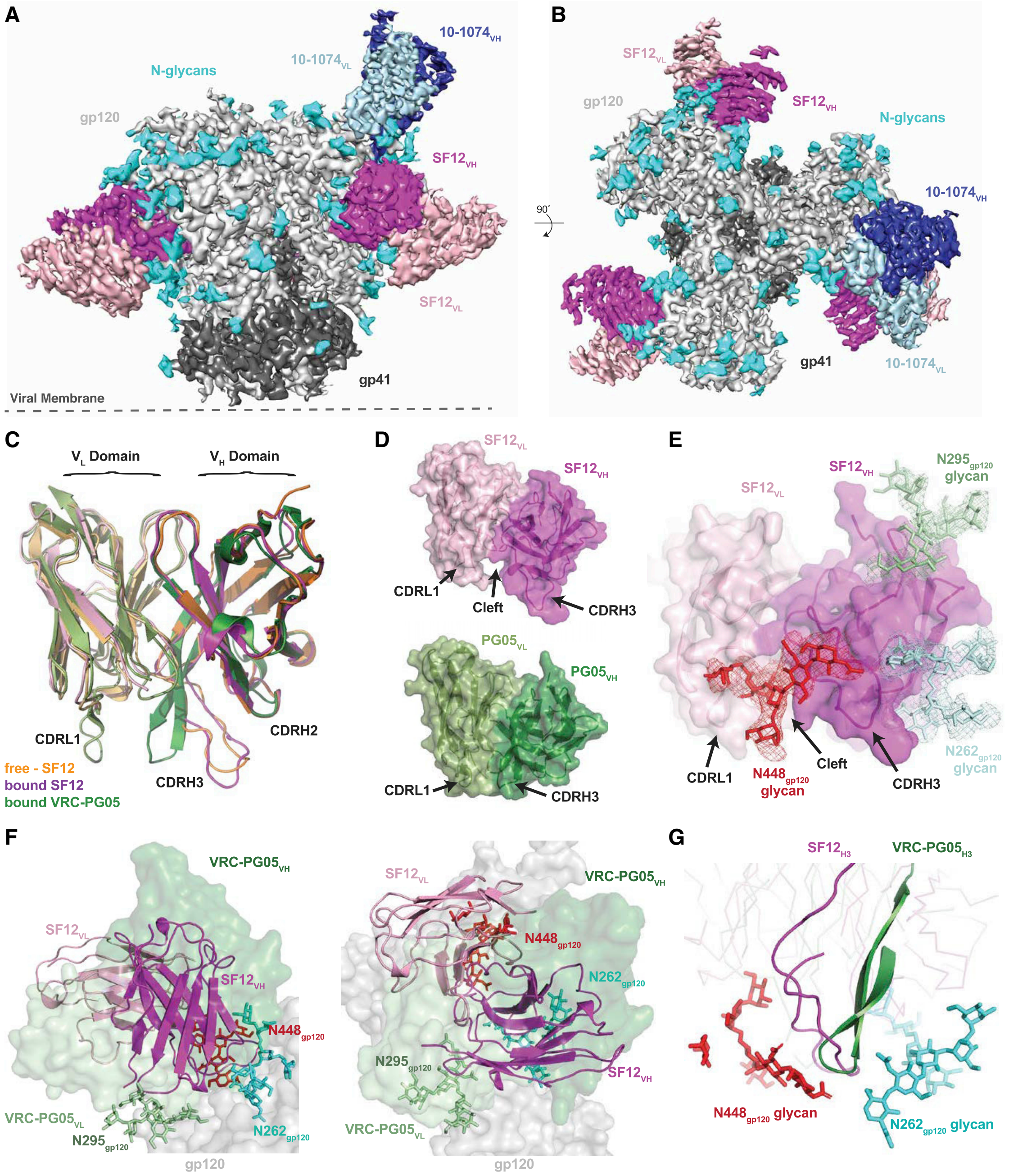

Figure 3. Structural Overview of the SF12-B41-10-1074 complex

(A and B) Side-view (A) and top-view (B) of the final 3.3 Å single-particle cryo-EM reconstruction of the SF12-B41-10-1074 complex colored by components (dark gray, gp41; light gray, gp120; magenta, SF12 V_H; pink, SF12 V_L; blue, 10-1074 V_H; light blue, 10-1074 V_L; cyan, N-glycans).

(C) Superposition of V_H-V_L domains (235 Cα atoms) of unliganded SF12 (orange), Env-bound SF12 (magenta), and core gp120-bound VRC-PG05 (green) Fabs, showing differences in CDR conformations between SF12 and VRC-PG05.

(D) Surface representation of SF12 (magenta/pink) and VRC-PG05 (green/pale green) Fabs illustrating differences in CDRL1 and CDRH3 loop conformations.

(E) Surface representation of Env-bound SF12 Fab showing interactions with the $N262_{gp120}$ (pale blue), $N295_{gp120}$ (pale green) and $N448_{gp120}$ (red) glycans at the SF12-Env interface. Cryo-EM density for individual glycans is shown contoured at 6σ.

(F) Comparison of V_H-V_L domain orientations of SF12 (magenta/pink; cartoon) and VRC-PG05 (green/pale green; surface). The V_H-V_L domain orientation of SF12 on Env trimer is related by a 71° rotation and 0.5 Å translation to the VRC-PG05 variable domains after alignment against gp120 (gray; surface).

(G) Overlay of CDRH3 loops of SF12 (magenta) and VRC-PG05 (green) after alignment of bound gp120s illustrates CDRH3-mediated recognition of the $N448_{gp120}$ glycan (red; sticks) by both antibodies.

See also Figures S2, S3, and S4 and Tables S3 and S4.

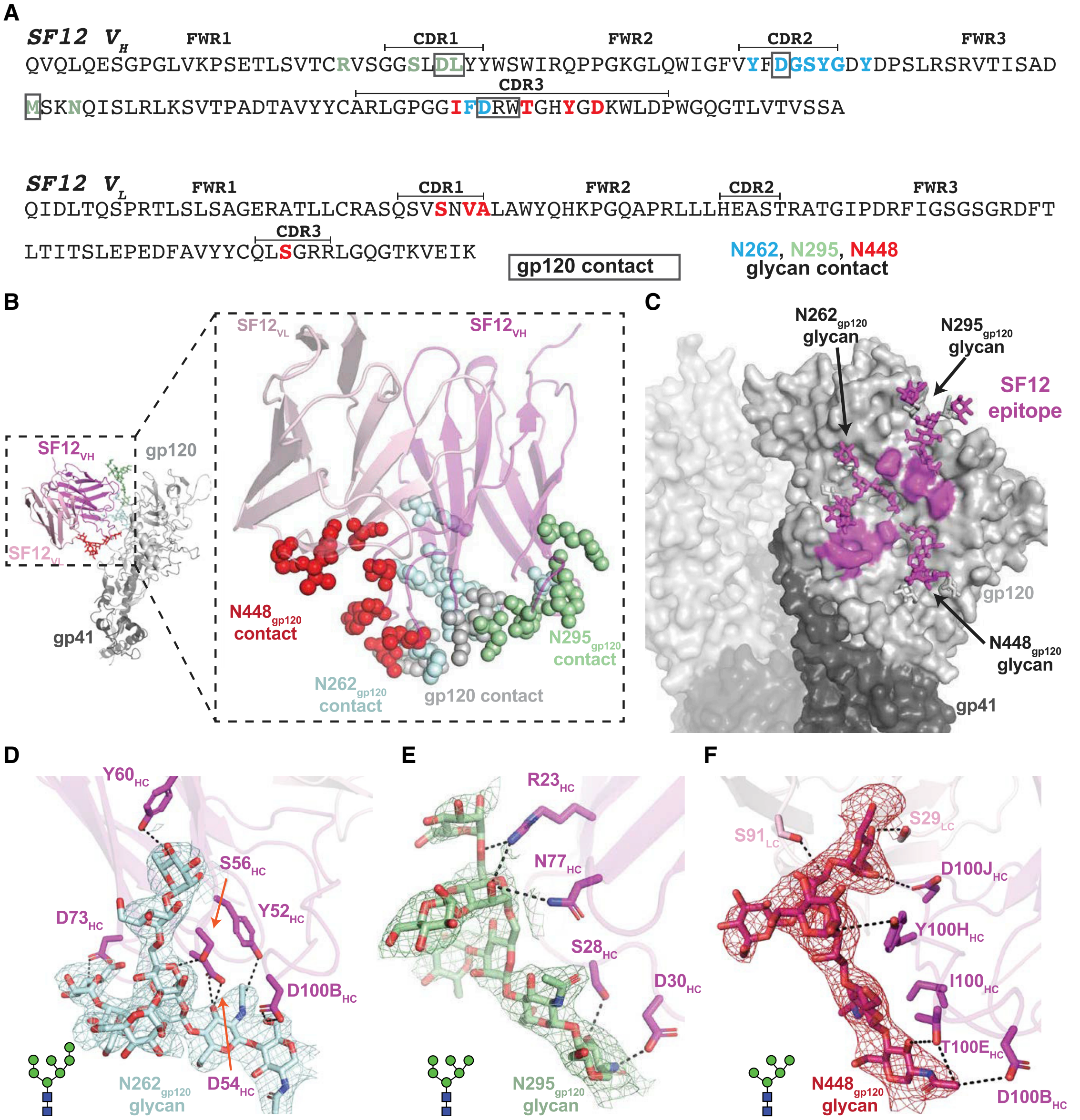

Figure 4. Details of SF12 Epitope and Glycan Recognition

(A) Sequence of SF12 variable domains with antibody regions annotated using IMGT sequence analysis (CDR loops are bracketed). SF12 residues that contact N-linked glycans are in blue ($N262_{gp120}$), green ($N295_{gp120}$), and red ($N448_{gp120}$), while gp120-contacting residues are boxed. Contacting residues in the SF12 paratope and epitope were defined as two residues containing any atom within 4 Å of each other.

(B) Structure of a SF12-B41 gp120 protomer from the trimer complex, showing paratope residues as spheres (inset). Color scheme is the same as in (A).

(C) Surface representation of B41 trimer, with SF12 epitope highlighted in magenta.

(D–F) Stick representation of residue level contacts for $N262_{gp120}$ (D), $N295_{gp120}$ (E), and $N448_{gp120}$ (F) glycans. Potential hydrogen bonds are shown as black dashes. Cryo-EM density maps contoured at 6σ are shown for individual glycans.

See also Figure S4 and Table S5.

VRC-PG05, such that interactions with both N-linked glycan and peptide components mapped almost exclusively to the SF12 heavy chain (Figures 4A–4C). For example, ~7% of the buried epitope surface resulted from interactions with SF12's light chain (~1,843 $Å^2$ buried surface area (BSA) of epitope against SF12 heavy chain versus ~135 $Å^2$ against the SF12 light chain; Table S5), compared with ~36% of buried epitope surface at the VRC-PG05 light chain interface. This difference is likely due to the longer CDRL1 and L3 loops on VRC-PG05, which penetrate the glycan-rich epitope (Figures 3C and 3D).

In the Env protomer used for epitope analysis, we interpreted densities for an ordered $GlcNAc_2Man_7$ at N262 gp_{120}, a $GlcNAc_2 Man_6$ at $N295_{gp120}$, and a $GlcNAc_2Man_5$ at $N448_{gp120}$ (Figures 4D–4F). Similar to VRC-PG05, the $N262_{gp120}$, $N295_{gp120}$, and $N448_{gp120}$ glycans constituted ~75% of the epitope surface (Table S5), although comparisons must be interpreted cautiously

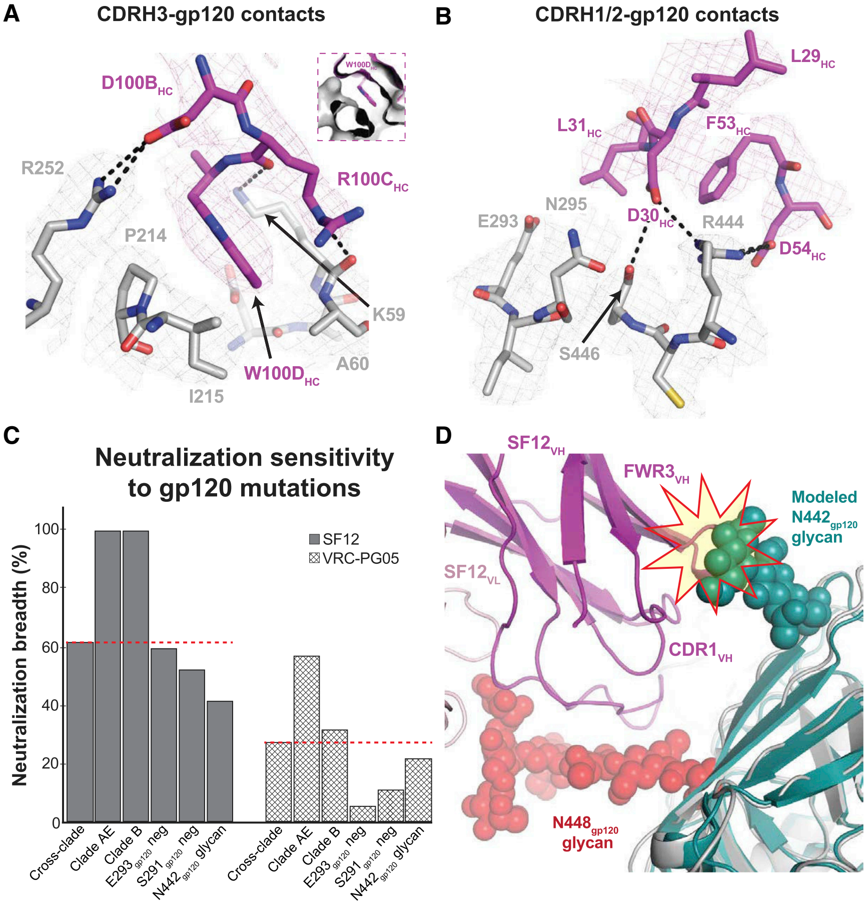

Figure 5. SF12 Engages Two Distinct Regions of gp120 Peptide Epitope

(A) Stick representation of SF12 CDRH3 (magenta) and gp120 (gray) contacts at the SF12-Env interface. Trp100D$_{HC}$ inserts into a hydrophobic pocket (inset) stabilized by potential hydrogen bond interactions (black dashes) with neighboring residues.

(B) Stick representation of SF12 CDRH1 and H2 residues (magenta) contacting gp120 residues (gray). Potential hydrogen bonds are shown as black dashes. Density maps for SF12 and gp120 residues are shown as magenta and gray meshes, respectively, contoured at 8σ.

(C) Comparison of SF12 and VRC-PG05 neutralization breadth for different viral characteristics. The red dashed line indicates neutralization breadth for SF12 (62%) and VRC-PG05 (27%) against a cross-clade panel.

(D) Modeling of the N442$_{gp120}$ glycan from clade C 426c SOSIP trimer (teal; PDB: 6MYY) was achieved by aligning gp120 coordinates from the two structures. Potential clashes with SF12 heavy chain (magenta) regions are highlighted.

See also Figure S5.

due to (1) the use of a high mannose-only monomeric gp120 core for the VRC-PG05 complex structure (Zhou et al., 2018) versus a natively glycosylated native-like Env trimer for the SF12 complex structure, and (2) the higher resolution (2.4 Å) of the VRC-PG05-gp120 crystal structure than that of the SF12-Env trimer cryo-EM structure (3.3 Å). Mapping key residues involved in SF12-Env interactions identified determinants of glycan recognition mediated by specific regions in the SF12 paratope. For example, SF12's CDRH1 and framework region 3 (FWR3) interacted exclusively with the N295$_{gp120}$ glycan, while CDRH2 solely engaged the N262$_{gp120}$ glycan (Figures 4D and 4E). SF12 interactions with the N448$_{gp120}$ glycan were mediated mainly by CDRH3, with additional contacts observed with the light chain CDRL1 and CDRL3 loops (Figure 4F).

Because the frequency of SHM in SF12 is lower than typical for many HIV-1 bNAbs (Figure 1E; Table S1), we analyzed the contributions of mutated amino acid residues in the SF12 paratope to epitope recognition. Of the 17 V gene segment-encoded residues that contact the epitope, 9 arose through SHM, including an insertion in CDRH2 (Figure S5A). Consistent with glycans comprising most of the SF12 epitope, SHMs were mostly observed for residues at the antibody-glycan interface. However, unlike VRC-PG05, where SHMs mainly resulted in the removal of bulky tyrosine residues to accommodate glycans (Zhou et al., 2018), SF12 utilized tyrosines, as well as bulky hydrophilic residues, to facilitate interactions with the glycopeptide epitope (Figure S5). This demonstrates that SHMs adding bulky residues to the paratopes of antibodies against the glycan-rich silent face of HIV-1 Env are not necessarily an impediment to broad and potent neutralization by these antibodies (Figures 4D–4F and S5A).

The protein component of the SF12 epitope (~25% of the epitope surface) mapped to two regions of gp120 (Figures 5 and S4). The first region involved residues from the gp120 β4 and β7 strands and the N terminus of gp120 that were engaged by regions of CDRH3 that penetrated the glycan shield (Figure 5A). In this interaction, SF12 CDRH3 residues R100C$_{HC}$ and D100B$_{HC}$ formed potential hydrogen bonds with Env residues K59$_{gp120}$ and R252$_{gp120}$, respectively (Figure 5A). These interactions contributed to the formation of a hydrophobic pocket on gp120 into which SF12 residue Trp100D$_{HC}$ inserted, shielding this exposed hydrophobic residue at the tip of the CDRH3 loop (Figure 5A, inset). The second protein component of the SF12 epitope resembled part of the VRC-PG05 gp120 protein epitope, involving residues from gp120 β12 and β22 that interacted with the SF12 CDRH1 and H2 loops (Figure 5B). In this region, SF12 utilized aspartates at positions 30$_{HC}$ and 54$_{HC}$ to mediate contacts with gp120 residues N295$_{gp120}$, R444$_{gp120}$, and S446$_{gp120}$ (Figure 5B). Unlike VRC-PG05, SF12 directly engaged the protein component of the epitope, forming extensive hydrogen bonds with surrounding residues. The increased epitope surface area contributed by gp120 peptide components (~25% for SF12 versus ~12% for VRC-PG05) likely contributes to the observed differences in neutralization potency and breadth for the two antibodies (Figure 1E). Overall, our structure of SF12 bound to a natively glycosylated Env trimer allows detailed insights into SF12 Env-glycan interactions

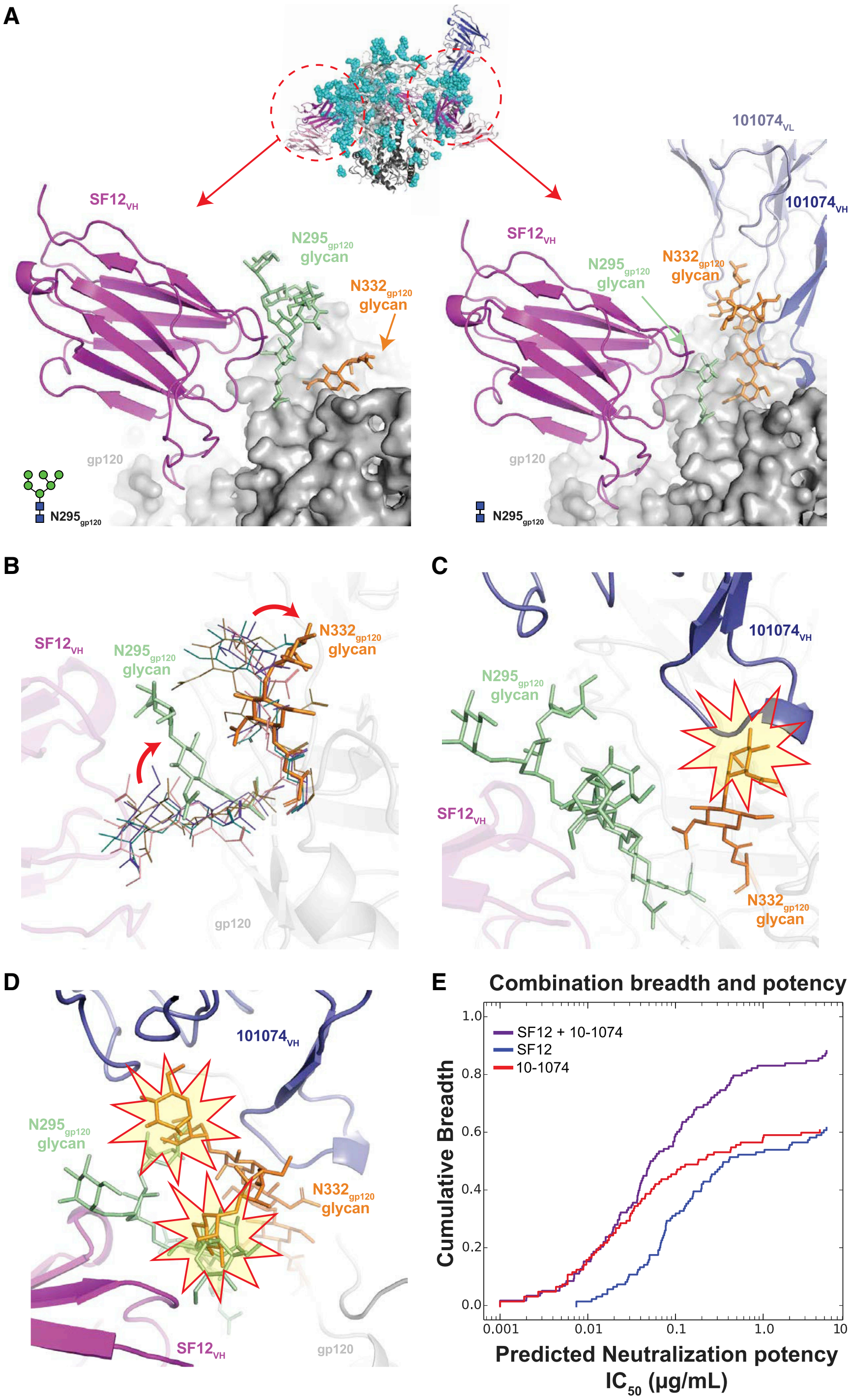

Figure 6. SF12-B41-10-1074 Structural Asymmetry Is Explained by N295$_{gp120}$ Glycan Heterogeneity

(A) Comparison of cryo-EM density for N295$_{gp120}$ (green) and N332$_{gp120}$ (orange) glycans across protomers within the SF12-B41-10-1074 trimer complex. In each protomer, SF12 (magenta) was bound, but 10-1074 (blue) binding was only observed when the N295$_{gp120}$ glycan was modeled as GlcNAc$_2$ (right panel).

(B) Overlay of N295$_{gp120}$ and N332$_{gp120}$ glycans after aligning gp120 protomers from cryo-EM structures of SF12-B41-10-1974, PDB: 6CUE, PDB: 6DCQ, PDB: 5V8M, and PDB: 6CRQ. Positions for the N295$_{gp120}$ and N332$_{gp120}$ glycans in the SF12-bound Env (stick representation) and all other models (line representation) are shown. SF12-induced conformational changes are indicated by the red arrow.

(C) Modeling of the 10-1074 Fab (blue cartoon) onto the SF12-gp120 protomer (A: left panel). Potential clashes between 10-1074 CDRH3 and the N332$_{gp120}$ glycan are highlighted.

(D) Alignment of gp120 portions of the SF12-bound (A: left panel) and SF12 plus 10-1074-bound (A: right panel) protomers. Potential clashes involving the N295$_{gp120}$ and N332$_{gp120}$ glycans (highlighted stars) when both glycans are processed beyond a core pentasaccharide are shown.

(E) Predictive neutralization profiles for combination therapy with SF12 and 10-1074 bNAbs at a 10 μg/mL concentration.

See also Figure S5 and Tables S6 and S7.

and demonstrates that SF12 forms more extensive protein contacts with Env than VRC-PG05.

Resistance to SF12 Is Driven by Glycan Contacts

To confirm our structural findings and assess possible mechanisms of resistance to SF12 and SF5 neutralization, we created a series of mutant BG505 and YU2 pseudoviruses and evaluated their sensitivity to neutralization in TZM.bl assays. Disruption of the PNGS at N448$_{gp120}$ (N448K, N448S) or N262$_{gp120}$ (N262S, N262W) abrogated SF5 and SF12 neutralization (Table S6). In contrast, removal of the glycan at 295$_{gp120}$ slightly improved neutralization by both SF5 and SF12 for the BG505 and YU2 strains, indicating that these antibodies accommodate, rather than make favorable contacts with, the glycan at N295$_{gp120}$. This result was consistent with neutralization data against strains lacking the N295$_{gp120}$ glycan (Table S7) and the SF12-B41 complex structure, as one gp140 protomer in the trimer showed density for GlcNA$_2$ at the N295$_{gp120}$ glycan despite the presence of bound SF12 Fab (Figure 6A).

To explore protein-protein interactions at the SF12-Env interface, we examined mutations in positions 214$_{gp120}$ (P214I, P214Q), 291$_{gp120}$ (S291P, S291T), 293$_{gp120}$ (Q/V293E, Q/V293K, Q/V293R), and 444$_{gp120}$ (R444T) in the Envs of the BG505 and YU2 pseudoviruses. Unlike VRC-PG05, where a dominant means to escape antibody neutralization was achieved by mutating E293$_{gp120}$ to disrupt a critical contact with VRC-PG05's CDRL1 and CDRH3 loops (Zhou et al., 2018), analysis of SF12 neutralization potency against the mutant pseudoviruses showed that SF12 remained potent against pseudoviruses with E293$_{gp120}$ substitutions (Figure 5C;

Tables S6 and S7). A 2-fold decrease in sensitivity to SF12 was observed for the R444T mutation, likely due to disruption of a hydrogen-bonding network between Env residue $R444_{gp120}$ and SF12 residues $D30_{HC}$ and $D54_{HC}$. Env mutations at positions 214_{gp120} and 293_{gp120} preferentially affected the SF5 clonal variant, which is less potent than SF12. Effects due to mutations at the 293_{gp120} residue in both BG505 and YU2 backbones are likely explained by the presence of $R31_{HC}$ in the SF5 CDRH1, which would directly engage with residue $E293_{gp120}$ (Figure S5B).

Interestingly, a threonine at position 444_{gp120} is a strong predictor of resistance to SF12/SF5 neutralization, consistent with increased activity against clade AE and B viruses, which show a <1% frequency for a threonine at this position (Figure 5C; Table S7). Computational analysis of viral strains containing residue $T444_{gp120}$ (37% of 3260 Env sequences in Antibody Database) (West et al., 2013) showed that 78% of the $T444_{gp120}$-containing sequences included an asparagine at position 442_{gp120} to create a 442_{gp120} PNGS. To determine whether a glycan at position $N442_{gp120}$ would disrupt SF12 binding, we modeled coordinates for the $N442_{gp120}$ glycan from the clade C 426c DS-SOSIP structure (Borst et al., 2018) after superposing the PDB 6MYY gp120 onto the gp120 of our Env trimer structure and adding in the $N442_{gp120}$ glycan. In the conformation observed on the 426c Env structure, the $N442_{gp120}$ glycan would clash with SF12 heavy chain CDRH1 and FWR3 components, sterically hindering access to its epitope (Figure 5D). This likely explains SF12/SF5's decreased breadth and potency against clade C viruses, 85% of which encode $T444_{gp120}$ (Table S7). However, binding of SF12 could shift the position of the glycan, as seen for the $N295_{gp120}$ glycan (Figures 6A and 6B), since SF12 shows neutralizing activity against some $N442_{gp120}$ glycan-containing viruses (Table S7). Our results indicate that resistance to SF12/SF5 is mediated mainly through mutation of N-glycan sites, rather than Env protein residues, as seen for VRC-PG05.

Heterogeneity of Glycan N295 Explains SF12-Env Complex Asymmetry

Given that previous V3-targeting bNAb structures showed symmetric binding of three V3-glycan patch Fabs per trimer (Ward and Wilson, 2017), including a recent cryo-EM structure of a BG505 DS-SOSIP bound by three PGT122 Fabs (Dingens et al., 2018), we sought to understand the asymmetric binding of the V3-directed 10-1074 Fab in the SF12-B41-10-074 structure (Figures 3A and 3B), noting that potential Env trimer asymmetry could not have been detected in the VRC-PG05 Fab-CNE55 complex structure, which was solved using a monomeric gp120 core (Zhou et al., 2018). Because asymmetric binding of SF12 and 10-1074 Fabs to the B41 Env trimer prevented the use of symmetry restraints during reconstruction and model building, density for each glycan at a PNGS was interpreted independently across gp140 protomers. Interestingly, glycan heterogeneity was observed at the $N295_{gp120}$ glycan, which correlated with the presence or absence of the 10-1074 Fab (Figure 6A).

Alignment of SF12-bound gp120 protomers in the absence and presence of 10-1074 Fab (Figure 6A, left and right panels, respectively), revealed a low (<0.2 Å) RMSD for 452 gp120 $C\alpha$ residues, suggesting that the lack of 10-1074 binding was not due to alteration in the protein portion of the V3 epitope. However, the $N332_{gp120}$ glycan exhibited an altered conformation in the SF12-gp140 protomers without a bound 10-1074 Fab. Indeed, superposition of gp120 coordinates from cryo-EM structures that included coordinates for the $N295_{gp120}$ and $N332_{gp120}$ glycans showed that SF12 binding induced conformational changes that resulted in the $N332_{gp12i0}$ glycan occluding the underlying protein portion of the V3-glycan patch epitope (Figure 6B). This conformation for the $N332_{gp120}$ glycan would prevent 10-1074 binding due to steric clashes (Figure 6C). Moreover, the $N295_{gp120}$ glycan conformation in the SF12-bound Env protomer and the $N332_{gp120}$ glycan conformation in the 10-1074-bound Env protomer would be incompatible if both glycans were present in fully processed forms (Figure 6D). Taken together, this explains why a 10-1074 Fab could be accommodated on one protomer in our trimer complex, because the $N295_{gp120}$ glycan appeared to be under-processed in the 10-1074-bound protomer ($GlcNAc_2$ versus $GlcNAc_2Man_6$ observed in the other protomers).

Moreover, Fab interactions with the $N295_{gp120}$ glycan likely explains observed differences in SF12 and SF5 binding properties, as SF5, but not SF12, was strongly competed by 10-1074 by ELISA (Figure 2B). SF5 includes a tyrosine in heavy chain FWR3 ($Y77_{HC}$), which arose through SHM to engage the core $GlcNAc_2$ of the $N295_{gp120}$ glycan (Figure S5C). It is possible that during accommodation of 10-1074 binding, movement of the $N295_{gp120}$ glycan toward SF5 Fab would clash with $Y77_{HC}$, compared to the less bulky $N77_{HC}$ in SF12 (Figure S5D). Despite the possibility for incompatible modes of binding to the same Env protomer with fully processed N-linked glycans at 295 and 332, SF12 and 10-1074 delivered as combination therapy could potentially achieve 90% breadth, as their neutralization patterns complement each another (Figure 6D). In sum, our SF12-Env trimer structure revealed N-glycan heterogeneity across different protomers of the same trimer, which was associated with differential binding to bNAbs.

Evaluation of SF12 in HIV-1-Infected Humanized Mice

We assessed the *in vivo* anti-HIV-1 activity of SF12 in humanized mice. Results from studies in mice are relevant to humans because clinical trials of HIV-1 bNAbs administered to infected patients showed that viral escape mutations are similar, if not identical, to those found in HIV-1-infected humanized mice (Bar-On et al., 2018; Caskey et al., 2015, 2017; Horwitz et al., 2013; Klein et al., 2012; Mendoza et al., 2018; Scheid et al., 2016). Mice were infected with HIV-1_{YU2} (Zhang et al., 2002) (n = 7) and subcutaneously administered 1 mg of SF12 IgG followed by 0.5 mg of SF12 every 3 days for 3 weeks. Untreated HIV-1_{YU2}-infected mice (n = 7) with comparable viral loads and matched stem cell donors served as controls. Mice treated with SF12 showed an average drop in viremia of 0.68 $\log_{10}$ copies/mL (range: 0.13–1.87 $\log_{10}$ copies/mL) with viral rebound occurring within 3 weeks after initiation of therapy (Figure 7A). To document escape mutations, single genome sequencing of gp160 was performed on mouse plasma samples 4 weeks after initiation of SF12 therapy. Of 29 independent isolates from 4 SF12-treated mice, we found mutations that abrogated the PNGS at position $N448_{gp120}$ in 28 sequences (97%). These mutations exclusively affected position 448_{gp120} and mutated the N at position 448_{gp120} to K, S, D, or I through single nucleotide

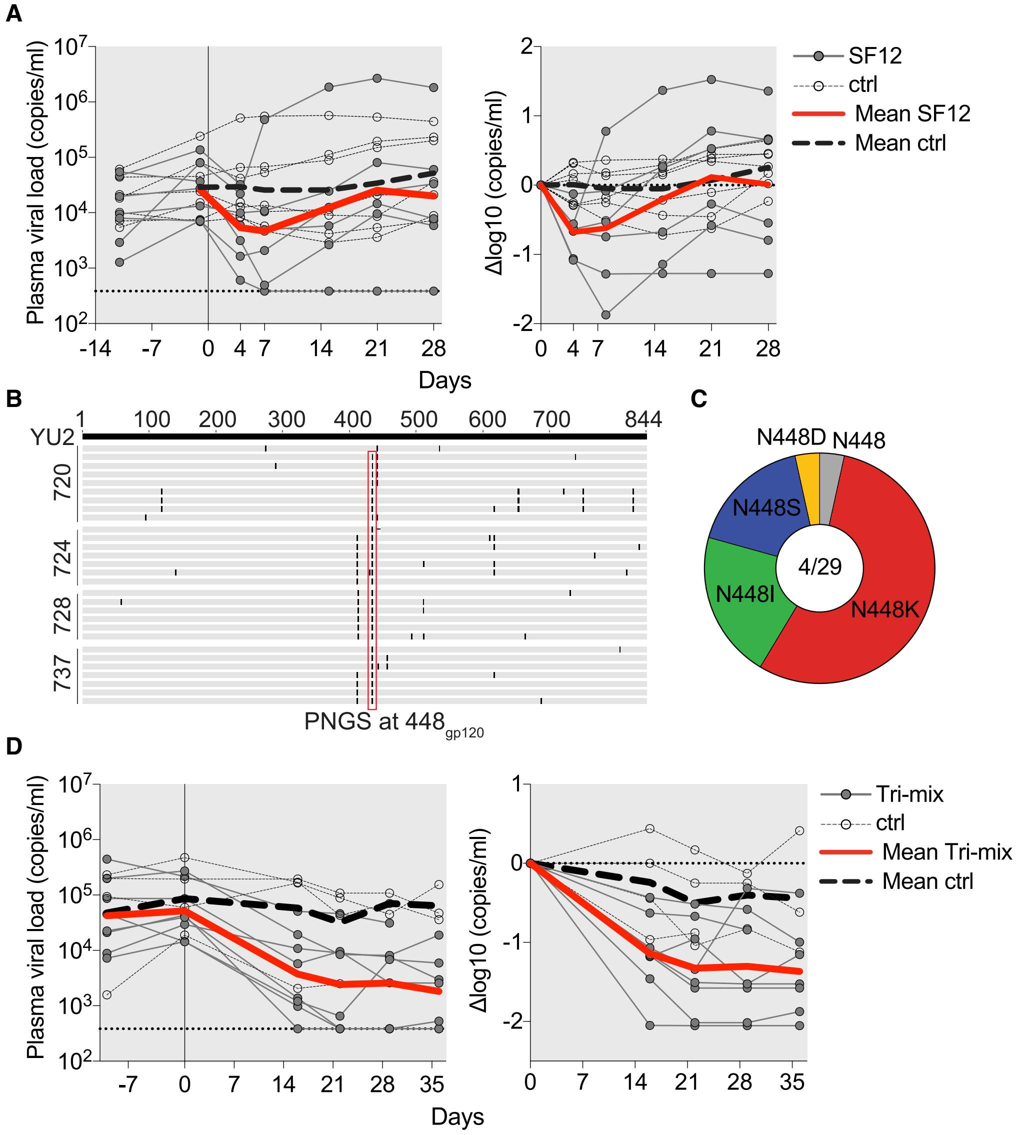

Figure 7. *In Vivo* Evaluation of SF12 IgG in HIV$_{YU2}$-Infected Humanized Mice

(A) SF12 monotherapy of humanized mice infected with HIV$_{YU2}$. The left graph shows absolute viremia (y axis) in mice treated with SF12 (n = 7, dark gray, full circles) or untreated control mice (n = 7, empty circles) over the course of the experiment (x axis, days). Mice were infected 3 weeks prior to therapy initiation and received 1 mg of IgG as a loading dose followed by twice-weekly administration of 0.5 mg for 3 weeks. The dotted line at bottom indicates the limit of accuracy of the qPCR assay (384 copies/mL). The right graph shows relative log drop after initiation of SF12 therapy (Δlog10 copies/mL). Thick red lines and thick dashed gray lines indicate the mean viral load of treated and untreated mice, respectively. Data from one independent experiment.

(B) Amino acid alignment of gp160 of wild-type YU2$_{gp160}$ (top) with Env gp160 sequences obtained by single genome sequencing from plasma of SF12-treated mice 4 weeks posttherapy initiation. Each line represents one sequence; mouse identification numbers indicated on left.

(C) Pie chart showing the amino acid distribution at position N448$_{gp120}$ in mice that received SF12 at 4 weeks posttherapy initiation. Numbers inside pie chart correspond to number of mice sequenced/number of sequences obtained.

(D) Antibody tri-mix (SF12, 10-1074, 3BNC117) therapy of HIV-1$_{YU2}$-infected mice (n = 8). Mice (n = 4) with comparable viral loads and matched stem cell donors served as controls. Data from one independent experiment. Graphs as in (A).

changes, with the mutation to K being most frequent (Figures 7B and 7C). We conclude that antibody SF12 can reduce viremia and exerts strong selective pressure on HIV-1 *in vivo*.

While antibody therapy with two effective bNAbs (PG16, NIH45-46^{G54W}) failed to mediate long-term viral suppression (Klein et al., 2012), a triple antibody regimen comprising 3BNC117, 10-1074, and PG16 suppressed viremia in infected humanized mice for several weeks (Horwitz et al., 2013). To determine whether SF12 can contribute to control of viremia in humanized mice as part of a triple combination regimen, we treated HIV-1$_{YU2}$-infected mice (n = 8) with a tri-mix of bNAbs SF12, 10-1074, and 3BNC117. Mice (n = 4) with comparable viral loads and matched stem cell donors served as controls. Mice receiving tri-mix therapy dropped 1.36 $\log_{10}$ copies/mL (range: 0.38–2.05

$\log_{10}$ copies/mL) during the 5 weeks of treatment. During the observation period, viremia was suppressed in all but one of the treated mice (Figure 7D). Thus, a triple antibody combination regimen that includes SF12 to restrict overall viral escape can mediate long-term control of HIV-1_{YU2} viremia *in vivo*.

DISCUSSION

Anti-HIV-1 bNAbs can control and prevent HIV-1 infection in humanized mice and macaques (Baba et al., 2000; Barouch et al., 2013; Hessell et al., 2009a, 2009b; Klein et al., 2012, 2014; Mascola et al., 2000; Shingai et al., 2013, 2014). In addition, bNAbs against two epitopes, the CD4-binding site and the V3-glycan patch, have been evaluated in humans: the antibodies were well-tolerated, exhibited typical IgG half-lives, and were effective in lowering viremia and preventing viral rebound in subjects undergoing analytical treatment interruption (Caskey et al., 2015, 2017; Lu et al., 2016; Lynch et al., 2015; Mendoza et al., 2018; Scheid et al., 2016; Schoofs et al., 2016). Anti-viral effects of bNAbs in pre-clinical models and humans are mediated by a combination of virus neutralization and Fcγ receptor-mediated elimination of infected cells (Halper-Stromberg et al., 2014; Hessell et al., 2007; Lu et al., 2016), highlighting the potential of Fc effector functions as modulators of bNAb efficacy. Fc-modified bNAb variants with increased affinity for the neonatal Fc receptor showed half-lives of more than 70 days in humans (Gaudinski et al., 2018), suggesting that passive administration of anti-HIV-1 bNAbs may be a practical therapeutic strategy.

However, administration of individual bNAbs, like monotherapy with anti-retroviral drugs, leads to emergence of resistant viral variants. In contrast, combinations of bNAbs can control infection for prolonged periods of time in mice, macaques and humans (Bar-On et al., 2018; Barouch et al., 2013; Horwitz et al., 2013; Klein et al., 2012; Mendoza et al., 2018; Shingai et al., 2013). Similarly, a fully protective vaccine will likely require elicitation of bNAbs targeting multiple HIV-1 Env epitopes. This highlights a need to identify and clinically develop antibodies to new epitopes on the Env spike. Here, we studied the antibody response of a long-term non-progressor, identifying bNAb SF12 as part of a family of antibodies that target a glycan-focused epitope on the silent face of gp120. While the silent face of Env had not been considered a priority target for bNAbs or for vaccine development, the discovery of potent and broad antibodies such as SF12 suggests that targeting this site could contribute to therapies and vaccines.

The cryo-EM structure of SF12 bound to B41 Env trimer demonstrated that SF12 makes extensive contacts with N-glycans at positions 262_{gp120}, 295_{gp120} and 448_{gp120}. While our neutralization and structural data indicated that the PNGS at 295_{gp120} is dispensable for SF12 neutralization, we found the highly conserved glycans at $N262_{gp120}$ and $N448_{gp120}$ to be obligate contacts. Although SF12's epitope is heavily glycan-focused, the antibody also contacts the protein backbone of Env, utilizing CDRHs 1 and 2 to mediate interactions at positions 293_{gp120}, 444_{gp120} and 446_{gp120}. In addition, SF12's longer CDRH3, as well as its different angle of Env approach relative to VRC-PG05, allow SF12 to form additional protein contacts at positions 59_{gp120}, 214_{gp120}, and 252_{gp120}, which likely explain the enhanced breadth and potency of SF12 compared to VRC-PG05, the only previously characterized antibody against the Env silent face (Zhou et al., 2018).

Consistent with previous observations suggesting that the $N262_{gp120}$ glycan plays a crucial role in CD4-mediated viral entry (Moore et al., 1994) and that the $N448_{gp120}$ glycan (>85% conserved) is less critical for viral infectivity (Behrens et al., 2016; Falkowska et al., 2014), our *in vivo* studies showed loss of $N448_{gp120}$ as the major escape pathway upon SF12 treatment. Interestingly, the protein-protein contacts made by SF12 did not contribute to routes of viral escape, which contrasts with HIV-1's dual mechanism of escape from VRC-PG05 by altering either the $N448_{gp120}$ glycan or the glycan-proximal residue $E293_{gp120}$. Indeed, viral evasion through alteration of the glycan shield or sequence diversity dominates most bNAb epitopes (Falkowska et al., 2014; Mouquet et al., 2012; Wagh et al., 2016; Walker et al., 2009). Thus, SF12 may represent one of the few glycopeptide recognizing bNAbs where sequence variability at the protein-protein interface does not induce viral resistance to SF12 neutralization.

Despite the highly conserved $N262_{gp120}$ and $N448_{gp120}$ glycans comprising its epitope, SF12 showed a gap in coverage that could not be explained by the removal of the $N448_{gp120}$ glycan alone. Computational analysis of data from a 119-isolate viral panel suggested that glycosylation at position $N442_{gp120}$ contributes to decreased SF12 breadth, likely through shielding of the $N262_{gp120}$ and $N448_{gp120}$ glycans, making it more difficult for SF12 to bind. SF12's distinct epitope makes it potentially useful for therapy or prevention as part of a combination of bNAbs, particular in regions of the world with a high prevalence of AE or B HIV-1 clades (both clades lack the $N442_{gp120}$ glycan). Indeed, the SF12 class of bNAbs is particularly potent and broad against clade AE viruses, which represent a coverage gap of the V3-glycan bNAbs, achieving 100% breadth and 10-fold increased potencies compared with VRC01. Furthermore, given its glycan-focused epitope, it may be possible to further improve SF12/SF5 potency, breadth, and resistance to escape using structure-based rational design as has been demonstrated for other anti-HIV-1 bNAbs (Diskin et al., 2011, 2013; Rudicell et al., 2014; Xu et al., 2017).

Our data support the hypothesis that evolutionary pathways to silent face recognition are diverse. In addition to being isolated from two different donors, SF12 and VRC-PG05 arose from highly divergent V_H (V_H4-59*01 versus V_H3-7*01) and V_K genes (V_K3-20*01 versus V_K4-1*01) (Lefranc et al., 2009). Moreover, SF12 uses a long CDRH3 of 23 residues and a relatively short CDRL3 of 6 residues, while VRC-PG05 uses a moderately long 17-residue CDRH3 and an average 8-residue CDRL3. Sequence conservation between the CDR3s of the two antibodies is low with no discernible motifs in common, which contrasts other anti-HIV-1 bNAb families that show strong CDRH3 or CDRL3 length restrictions and/or recurring sequence motifs (McCoy and Burton, 2017). In addition, SF12 shows an intermediate rate of SHM compared with other HIV-1 bNAbs, with 21 and 19 amino acid substitutions in the heavy and light chains, respectively. This rate of mutation is comparable to or lower than rates found in V3-glycan patch antibodies, which are a heavily pursued vaccination target with a similar breadth of coverage (Escolano et al., 2017; McCoy and Burton, 2017). Taken together, these differences illustrate the divergence of

solutions evolved by the SF12/SF5 and VRC-PG05 bNAbs to target the silent face epitope, whereby SF12's maturation generated an antibody capable of accommodating Env sequence diversity at the protein surface.

In the context of eliciting silent face bNAbs, SF12 represents a promising path forward based on its breadth, potency, and relatively few SHMs. However, eliciting SF12-like bNAbs may face some of the same hurdles as other potential Env target sites, given its long CDRH3 and an insertion in CDRH2. In addition, the SF12-Env structure revealed that, despite targeting non-overlapping epitopes, SF12 binding induced a conformational change in the $N295_{gp120}$ glycan that affected binding of the V3-glycan bNAb 10-1074. Thus, trimer-based immunogen design strategies, such as those employed to elicit V3-glycan targeting bNAbs (Escolano et al., 2017), should consider how alterations in the glycan shield around the silent face glycan patch may prevent maturation of SF12-like bNAbs.

Zhou et al. (2018) estimated VRC-PG05-like bNAbs are present in 10% of individuals in a 38-donor cohort. It is possible that SF antibodies were missed in previous studies, since the focus was on isolating antibodies to well-characterized epitopes. In particular, protein-based sorting strategies might not have allowed identification of these antibodies as also seen in this study, in which only two members of the clone were isolated by sorting using BG505 Env trimer as bait.

In summary, our results show that the glycan-rich silent face of HIV-1 Env can be targeted by bNAbs with potencies and breadths approaching those of antibodies against some of the more well-characterized epitopes. Overall, our findings extend current understanding of the recognition of glycan-focused epitopes on HIV-1 Env and expand the armamentarium of bNAbs available for HIV-1 therapy, prevention, and immunogen design.

STAR★METHODS

Detailed methods are provided in the online version of this paper and include the following:

- KEY RESOURCES TABLE
- CONTACT FOR REAGENT AND RESOURCE SHARING
- EXPERIMENTAL MODEL AND SUBJECT DETAILS
  - Human subjects
  - Humanized mice
  - Cell lines
- METHODS DETAILS
  - IgG isolation for polyclonal IgG neutralization testing
  - Neutralization fingerprinting analysis
  - B cell microculture
  - Single B cell bait-sorting
  - Phylogenetic analysis of SF family heavy chain sequences
  - Antibody production for ELISA, neutralization assays and *in vivo* experiments
  - Enzyme-linked immunosorbent assay (ELISA) of (mutant) YU2 gp120/gp140 proteins
  - BG505 SOSIP.664-His ELISAs
  - Competition ELISAs
  - Generation of mutant HIV_{YU2} and HIV_{BG505} pseudoviruses
  - *In vitro* neutralization assays
  - Autoreactivity and polyreactivity assays
  - Single genome sequencing of plasma HIV-1 Env genes
  - Protein expression and purification for structural studies
  - Crystal structure of SF12 Fab
  - Cryo-EM sample preparation
  - Cryo-EM data collection and processing
  - Modeling and refinement of cryo-EM structures
  - Structural and bioinformatic analyses
- DATA AND SOFTWARE AVAILABILITY

SUPPLEMENTAL INFORMATION

Supplemental Information can be found online at https://doi.org/10.1016/j.immuni.2019.04.014.

ACKNOWLEDGMENTS

We thank members of the Bjorkman, Nussenzweig, and Klein labs for helpful discussions. We also thank Gaëlle Breton and Kristie Gordon for help with flow cytometry sorting, and Anna Gazumyan, Hanna Janicki, Carola Ruping, Kanika Vanshylla, and Daniela Weiland for technical assistance. BG505 SOSIP.664-Avi, BG505 SOSIP.664-His, the CHO cell line expressing the B41 SOSIP Trimer, and the BG505.T332N gp160 expression plasmid were kind gifts of Albert Cupo, John P. Moore, and Rogier W. Sanders. The two protocols that subject 27845 was enrolled in were funded by the NIH (UM1 AI068618 and P01 AI057005) and the Bill and Melinda Gates Foundation. Structural studies were assisted by the Caltech Molecular Observatory (Dr. Jens Kaiser, director) and the Biological and Cryogenic Transmission Electron Microscopy Center at Caltech (Drs. Andrey Malyutin and Songye Chen, directors). Part of the cryo-EM work was performed at the Cold Spring Harbor Laboratories Course of Cryo-EM Methods with help from Drs. Gabriel Lander, Melanie Ohi, Matthjin Vos, David Veesler, and Justin Kollman. We thank the Gordon and Betty Moore and Beckman Foundations for gifts to Caltech to support the Molecular Observatory and electron microscopy. This work was supported by the National Institute of Allergy and Infectious Diseases of the NIH (HIVRAD P01 AI100148 to P.J.B. and M.C.N. and R01 AI131722 to I.S.G.); the NIH (P50 GM082545-06 to P.J.B.); the Bill and Melinda Gates Foundation Collaboration for AIDS Vaccine Discovery (OPP1124068 to M.C.N. and P.J.B. and 1146996 to M.S.S.); the NIH Center for HIV/AIDS Vaccine Immunology and Immunogen Discovery (CHAVI-ID) (1UM1 AI100663-01 to M.C.N.); the European Research Council (ERC-StG639961 to F.K.); the German Center for Infection Research (DZIF) (to F.K.); and in part by the intramural research program of the Vaccine Research Center, NIAID/NIH (to J.R.M.). T.S. was supported by the Ernst Jung Career Advancement Award for Medical Research, the National Center for Advancing Translational Sciences (NCATS, NIH Clinical and Translational Science Award [CTSA] program, UL1 TR001866), and the DZIF (TI 07.002). C.O.B was supported by the Hanna Gray Fellowship Program from the Howard Hughes Medical Institute and the Postdoctoral Enrichment Program from the Burroughs Wellcome Fund. P.S. and H.G. were supported by the DZIF. M.C.N. is a HHMI investigator.

AUTHOR CONTRIBUTIONS

T.S., C.O.B., N.A.D.-R., J.R.M., F.K., M.C.N., and P.J.B. conceived the study and analyzed data. J.C. and M.J.M. supervised clinical protocols and provided samples from donor 27845. L.N. isolated immunoglobulin for analysis. I.S.G. carried out neutralization fingerprinting. N.A.D.-R. and T.S. performed B cell microcultures. R.T.B. carried out microculture neutralization analysis. J.G. and T.S. performed single B cell bait-sorting and B cell cloning. M.S.S. performed large panel neutralization testing. A.P.W. computationally analyzed neutralization data. T.S. performed ELISAs and generated pseudoviruses for site mutant analysis. P.S. performed neutralization testing on site mutants, and some global panel testing of family members. N.S.-T. and C.O.B. generated the Fab structure. C.O.B. generated and analyzed the cryo-EM structure

of the SF12-B41 complex. Y.E.L. and T.S. performed polyreactivity and autoreactivity assays. T.S. and H.G. performed mouse experiments. F.B. humanized mice and screened them for humanization. F.K. designed and supervised mouse experiments. T.S., C.O.B., M.C.N., and P.J.B. wrote the manuscript with contributions from other authors.

DECLARATION OF INTERESTS

There are patents on 3BNC117 and 10-1074 on which M.C.N. and P.J.B. are inventors.

Received: January 16, 2019
Revised: March 20, 2019
Accepted: April 26, 2019
Published: May 21, 2019

REFERENCES

Adams, P.D., Afonine, P.V., Bunkóczi, G., Chen, V.B., Davis, I.W., Echols, N., Headd, J.J., Hung, L.W., Kapral, G.J., Grosse-Kunstleve, R.W., et al. (2010). PHENIX: a comprehensive Python-based system for macromolecular structure solution. Acta Crystallogr. D Biol. Crystallogr. *66*, 213–221.

Agirre, J., Iglesias-Fernández, J., Rovira, C., Davies, G.J., Wilson, K.S., and Cowtan, K.D. (2015). Privateer: software for the conformational validation of carbohydrate structures. Nat. Struct. Mol. Biol. *22*, 833–834.

Baba, T.W., Liska, V., Hofmann-Lehmann, R., Vlasak, J., Xu, W., Ayehunie, S., Cavacini, L.A., Posner, M.R., Katinger, H., Stiegler, G., et al. (2000). Human neutralizing monoclonal antibodies of the IgG1 subtype protect against mucosal simian-human immunodeficiency virus infection. Nat. Med. *6*, 200–206.

Bar, K.J., Sneller, M.C., Harrison, L.J., Justement, J.S., Overton, E.T., Petrone, M.E., Salantes, D.B., Seamon, C.A., Scheinfeld, B., Kwan, R.W., et al. (2016). Effect of HIV Antibody VRC01 on Viral Rebound after Treatment Interruption. N. Engl. J. Med. *375*, 2037–2050.

Bar-On, Y., Gruell, H., Schoofs, T., Pai, J.A., Nogueira, L., Butler, A.L., Millard, K., Lehmann, C., Suárez, I., Oliveira, T.Y., et al. (2018). Safety and antiviral activity of combination HIV-1 broadly neutralizing antibodies in viremic individuals. Nat. Med. *24*, 1701–1707.

Barouch, D.H., Whitney, J.B., Moldt, B., Klein, F., Oliveira, T.Y., Liu, J., Stephenson, K.E., Chang, H.W., Shekhar, K., Gupta, S., et al. (2013). Therapeutic efficacy of potent neutralizing HIV-1-specific monoclonal antibodies in SHIV-infected rhesus monkeys. Nature *503*, 224–228.

Behrens, A.J., Vasiljevic, S., Pritchard, L.K., Harvey, D.J., Andev, R.S., Krumm, S.A., Struwe, W.B., Cupo, A., Kumar, A., Zitzmann, N., et al. (2016). Composition and Antigenic Effects of Individual Glycan Sites of a Trimeric HIV-1 Envelope Glycoprotein. Cell Rep. *14*, 2695–2706.

Bell, J.M., Chen, M., Baldwin, P.R., and Ludtke, S.J. (2016). High resolution single particle refinement in EMAN2.1. Methods *100*, 25–34.

Borst, A.J., Weidle, C.E., Gray, M.D., Frenz, B., Snijder, J., Joyce, M.G., Georgiev, I.S., Stewart-Jones, G.B., Kwong, P.D., McGuire, A.T., et al. (2018). Germline VRC01 antibody recognition of a modified clade C HIV-1 envelope trimer and a glycosylated HIV-1 gp120 core. eLife *7*, e37688.

Brunger, A.T. (2007). Version 1.2 of the Crystallography and NMR system. Nat. Protoc. *2*, 2728–2733.

Burton, D.R. (2002). Antibodies, viruses and vaccines. Nat. Rev. Immunol. *2*, 706–713.

Burton, D.R., and Hangartner, L. (2016). Broadly Neutralizing Antibodies to HIV and Their Role in Vaccine Design. Annu. Rev. Immunol. *34*, 635–659.

Caskey, M., Klein, F., Lorenzi, J.C., Seaman, M.S., West, A.P., Jr., Buckley, N., Kremer, G., Nogueira, L., Braunschweig, M., Scheid, J.F., et al. (2015). Viraemia suppressed in HIV-1-infected humans by broadly neutralizing antibody 3BNC117. Nature *522*, 487–491.

Caskey, M., Schoofs, T., Gruell, H., Settler, A., Karagounis, T., Kreider, E.F., Murrell, B., Pfeifer, N., Nogueira, L., Oliveira, T.Y., et al. (2017). Antibody 10-1074 suppresses viremia in HIV-1-infected individuals. Nat. Med. *23*, 185–191.

Chen, V.B., Arendall, W.B., 3rd, Headd, J.J., Keedy, D.A., Immormino, R.M., Kapral, G.J., Murray, L.W., Richardson, J.S., and Richardson, D.C. (2010). MolProbity: all-atom structure validation for macromolecular crystallography. Acta Crystallogr. D Biol. Crystallogr. *66*, 12–21.

Chung, N.P., Matthews, K., Kim, H.J., Ketas, T.J., Golabek, M., de Los Reyes, K., Korzun, J., Yasmeen, A., Sanders, R.W., Klasse, P.J., et al. (2014). Stable 293 T and CHO cell lines expressing cleaved, stable HIV-1 envelope glycoprotein trimers for structural and vaccine studies. Retrovirology *11*, 33.

de Taeye, S.W., Ozorowski, G., Torrents de la Peña, A., Guttman, M., Julien, J.P., van den Kerkhof, T.L., Burger, J.A., Pritchard, L.K., Pugach, P., Yasmeen, A., et al. (2015). Immunogenicity of Stabilized HIV-1 Envelope Trimers with Reduced Exposure of Non-neutralizing Epitopes. Cell *163*, 1702–1715.

deCamp, A., Hraber, P., Bailer, R.T., Seaman, M.S., Ochsenbauer, C., Kappes, J., Gottardo, R., Edlefsen, P., Self, S., Tang, H., et al. (2014). Global panel of HIV-1 Env reference strains for standardized assessments of vaccine-elicited neutralizing antibodies. J. Virol. *88*, 2489–2507.

Dingens, A.S., Acharya, P., Haddox, H.K., Rawi, R., Xu, K., Chuang, G.Y., Wei, H., Zhang, B., Mascola, J.R., Carragher, B., et al. (2018). Complete functional mapping of infection- and vaccine-elicited antibodies against the fusion peptide of HIV. PLoS Pathog. *14*, e1007159.

Diskin, R., Klein, F., Horwitz, J.A., Halper-Stromberg, A., Sather, D.N., Marcovecchio, P.M., Lee, T., West, A.P., Jr., Gao, H., Seaman, M.S., et al. (2013). Restricting HIV-1 pathways for escape using rationally designed anti-HIV-1 antibodies. J. Exp. Med. *210*, 1235–1249.

Diskin, R., Scheid, J.F., Marcovecchio, P.M., West, A.P., Jr., Klein, F., Gao, H., Gnanapragasam, P.N., Abadir, A., Seaman, M.S., Nussenzweig, M.C., and Bjorkman, P.J. (2011). Increasing the potency and breadth of an HIV antibody by using structure-based rational design. Science *334*, 1289–1293.

Doria-Rose, N.A., Altae-Tran, H.R., Roark, R.S., Schmidt, S.D., Sutton, M.S., Louder, M.K., Chuang, G.Y., Bailer, R.T., Cortez, V., Kong, R., et al. (2017). Mapping Polyclonal HIV-1 Antibody Responses via Next-Generation Neutralization Fingerprinting. PLoS Pathog. *13*, e1006148.

Doria-Rose, N.A., Bhiman, J.N., Roark, R.S., Schramm, C.A., Gorman, J., Chuang, G.Y., Pancera, M., Cale, E.M., Ernandes, M.J., Louder, M.K., et al. (2015). A new member of the V1V2-directed CAP256-VRC26 lineage that shows increased breadth and exceptional potency. J. Virol. *90*, 76–91.

Doria-Rose, N.A., Klein, R.M., Daniels, M.G., O'Dell, S., Nason, M., Lapedes, A., Bhattacharya, T., Migueles, S.A., Wyatt, R.T., Korber, B.T., et al. (2010). Breadth of human immunodeficiency virus-specific neutralizing activity in sera: clustering analysis and association with clinical variables. J. Virol. *84*, 1631–1636.

Dosenovic, P., von Boehmer, L., Escolano, A., Jardine, J., Freund, N.T., Gitlin, A.D., McGuire, A.T., Kulp, D.W., Oliveira, T., Scharf, L., et al. (2015). Immunization for HIV-1 Broadly Neutralizing Antibodies in Human Ig Knockin Mice. Cell *161*, 1505–1515.

Emsley, P., and Cowtan, K. (2004). Coot: model-building tools for molecular graphics. Acta Crystallogr. D Biol. Crystallogr. *60*, 2126–2132.

Emsley, P., Lohkamp, B., Scott, W.G., and Cowtan, K. (2010). Features and development of Coot. Acta Crystallogr. D Biol. Crystallogr. *66*, 486–501.

Escolano, A., Dosenovic, P., and Nussenzweig, M.C. (2017). Progress toward active or passive HIV-1 vaccination. J. Exp. Med. *214*, 3–16.

Falkowska, E., Le, K.M., Ramos, A., Doores, K.J., Lee, J.H., Blattner, C., Ramirez, A., Derking, R., van Gils, M.J., Liang, C.H., et al. (2014). Broadly neutralizing HIV antibodies define a glycan-dependent epitope on the prefusion conformation of gp41 on cleaved envelope trimers. Immunity *40*, 657–668.

Freund, N.T., Wang, H., Scharf, L., Nogueira, L., Horwitz, J.A., Bar-On, Y., Golijanin, J., Sievers, S.A., Sok, D., Cai, H., et al. (2017). Coexistence of potent HIV-1 broadly neutralizing antibodies and antibody-sensitive viruses in a viremic controller. Sci. Transl. Med. *9*, eaal2144.

Gaudinski, M.R., Houser, K.V., Morabito, K.M., Hu, Z., Yamshchikov, G., Rothwell, R.S., Berkowitz, N., Mendoza, F., Saunders, J.G., Novik, L., et al.; VRC 319; VRC 320 study teams (2018). Safety, tolerability, and immunogenicity

of two Zika virus DNA vaccine candidates in healthy adults: randomised, open-label, phase 1 clinical trials. Lancet *391*, 552–562.

Gautam, R., Nishimura, Y., Pegu, A., Nason, M.C., Klein, F., Gazumyan, A., Golijanin, J., Buckler-White, A., Sadjadpour, R., Wang, K., et al. (2016). A single injection of anti-HIV-1 antibodies protects against repeated SHIV challenges. Nature *533*, 105–109.

Georgiev, I.S., Doria-Rose, N.A., Zhou, T., Kwon, Y.D., Staupe, R.P., Moquin, S., Chuang, G.Y., Louder, M.K., Schmidt, S.D., Altae-Tran, H.R., et al. (2013). Delineating antibody recognition in polyclonal sera from patterns of HIV-1 isolate neutralization. Science *340*, 751–756.

Goddard, T.D., Huang, C.C., and Ferrin, T.E. (2007). Visualizing density maps with UCSF Chimera. J. Struct. Biol. *157*, 281–287.

Gray, E.S., Madiga, M.C., Hermanus, T., Moore, P.L., Wibmer, C.K., Tumba, N.L., Werner, L., Mlisana, K., Sibeko, S., Williamson, C., et al. (2011). The Neutralization Breadth of HIV-1 Develops Incrementally over Four Years and Is Associated with CD4+ T Cell Decline and High Viral Load during Acute Infection. J. Virol. *85*, 4828–4840.

Gristick, H.B., von Boehmer, L., West, A.P., Jr., Schamber, M., Gazumyan, A., Golijanin, J., Seaman, M.S., Fätkenheuer, G., Klein, F., Nussenzweig, M.C., and Bjorkman, P.J. (2016). Natively glycosylated HIV-1 Env structure reveals new mode for antibody recognition of the CD4-binding site. Nat. Struct. Mol. Biol. *23*, 906–915.

Gruell, H., and Klein, F. (2018). Antibody-mediated prevention and treatment of HIV-1 infection. Retrovirology *15*, 73.

Halper-Stromberg, A., Lu, C.L., Klein, F., Horwitz, J.A., Bournazos, S., Nogueira, L., Eisenreich, T.R., Liu, C., Gazumyan, A., Schaefer, U., et al. (2014). Broadly neutralizing antibodies and viral inducers decrease rebound from HIV-1 latent reservoirs in humanized mice. Cell *158*, 989–999.

Haynes, B.F. (2015). New approaches to HIV vaccine development. Curr. Opin. Immunol. *35*, 39–47.

Haynes, B.F., and Burton, D.R. (2017). Developing an HIV vaccine. Science *355*, 1129–1130.

Haynes, B.F., Fleming, J., St Clair, E.W., Katinger, H., Stiegler, G., Kunert, R., Robinson, J., Scearce, R.M., Plonk, K., Staats, H.F., et al. (2005). Cardiolipin polyspecific autoreactivity in two broadly neutralizing HIV-1 antibodies. Science *308*, 1906–1908.

Hessell, A.J., Hangartner, L., Hunter, M., Havenith, C.E., Beurskens, F.J., Bakker, J.M., Lanigan, C.M., Landucci, G., Forthal, D.N., Parren, P.W., et al. (2007). Fc receptor but not complement binding is important in antibody protection against HIV. Nature *449*, 101–104.

Hessell, A.J., Poignard, P., Hunter, M., Hangartner, L., Tehrani, D.M., Bleeker, W.K., Parren, P.W., Marx, P.A., and Burton, D.R. (2009a). Effective, low-titer antibody protection against low-dose repeated mucosal SHIV challenge in macaques. Nat. Med. *15*, 951–954.

Hessell, A.J., Rakasz, E.G., Poignard, P., Hangartner, L., Landucci, G., Forthal, D.N., Koff, W.C., Watkins, D.I., and Burton, D.R. (2009b). Broadly neutralizing human anti-HIV antibody 2G12 is effective in protection against mucosal SHIV challenge even at low serum neutralizing titers. PLoS Pathog. *5*, e1000433.

Horwitz, J.A., Bar-On, Y., Lu, C.L., Fera, D., Lockhart, A.A.K., Lorenzi, J.C.C., Nogueira, L., Golijanin, J., Scheid, J.F., Seaman, M.S., et al. (2017). Non-neutralizing Antibodies Alter the Course of HIV-1 Infection In Vivo. Cell *170*, 637–648.

Horwitz, J.A., Halper-Stromberg, A., Mouquet, H., Gitlin, A.D., Tretiakova, A., Eisenreich, T.R., Malbec, M., Gravemann, S., Billerbeck, E., Dorner, M., et al. (2013). HIV-1 suppression and durable control by combining single broadly neutralizing antibodies and antiretroviral drugs in humanized mice. Proc. Natl. Acad. Sci. USA *110*, 16538–16543.

Hötzel, I., Theil, F.P., Bernstein, L.J., Prabhu, S., Deng, R., Quintana, L., Lutman, J., Sibia, R., Chan, P., Bumbaca, D., et al. (2012). A strategy for risk mitigation of antibodies with fast clearance. MAbs *4*, 753–760.

Hraber, P., Seaman, M.S., Bailer, R.T., Mascola, J.R., Montefiori, D.C., and Korber, B.T. (2014). Prevalence of broadly neutralizing antibody responses during chronic HIV-1 infection. AIDS *28*, 163–169.

Huang, J., Doria-Rose, N.A., Longo, N.S., Laub, L., Lin, C.L., Turk, E., Kang, B.H., Migueles, S.A., Bailer, R.T., Mascola, J.R., and Connors, M. (2013). Isolation of human monoclonal antibodies from peripheral blood B cells. Nat. Protoc. *8*, 1907–1915.

Jardine, J., Julien, J.P., Menis, S., Ota, T., Kalyuzhniy, O., McGuire, A., Sok, D., Huang, P.S., MacPherson, S., Jones, M., et al. (2013). Rational HIV immunogen design to target specific germline B cell receptors. Science *340*, 711–716.

Jeong, J.Y., Yim, H.S., Ryu, J.Y., Lee, H.S., Lee, J.H., Seen, D.S., and Kang, S.G. (2012). One-step sequence- and ligation-independent cloning as a rapid and versatile cloning method for functional genomics studies. Appl. Environ. Microbiol. *78*, 5440–5443.

Julg, B., Liu, P.T., Wagh, K., Fischer, W.M., Abbink, P., Mercado, N.B., Whitney, J.B., Nkolola, J.P., McMahan, K., Tartaglia, L.J., et al. (2017). Protection against a mixed SHIV challenge by a broadly neutralizing antibody cocktail. Sci. Transl. Med. *9*, eaao4235.

Kabsch, W. (2010). XDS. Acta Crystallogr. D Biol. Crystallogr. *66*, 125–132.

Keele, B.F., Giorgi, E.E., Salazar-Gonzalez, J.F., Decker, J.M., Pham, K.T., Salazar, M.G., Sun, C., Grayson, T., Wang, S., Li, H., et al. (2008). Identification and characterization of transmitted and early founder virus envelopes in primary HIV-1 infection. Proc. Natl. Acad. Sci. USA *105*, 7552–7557.

Klein, F., Halper-Stromberg, A., Horwitz, J.A., Gruell, H., Scheid, J.F., Bournazos, S., Mouquet, H., Spatz, L.A., Diskin, R., Abadir, A., et al. (2012). HIV therapy by a combination of broadly neutralizing antibodies in humanized mice. Nature *492*, 118–122.

Klein, F., Nogueira, L., Nishimura, Y., Phad, G., West, A.P., Jr., Halper-Stromberg, A., Horwitz, J.A., Gazumyan, A., Liu, C., Eisenreich, T.R., et al. (2014). Enhanced HIV-1 immunotherapy by commonly arising antibodies that target virus escape variants. J. Exp. Med. *211*, 2361–2372.

Klein, J.S., and Bjorkman, P.J. (2010). Few and far between: how HIV may be evading antibody avidity. PLoS Pathog. *6*, e1000908.

Krissinel, E., and Henrick, K. (2007). Inference of macromolecular assemblies from crystalline state. J. Mol. Biol. *372*, 774–797.

Kwong, P.D., and Mascola, J.R. (2018). HIV-1 Vaccines Based on Antibody Identification, B Cell Ontogeny, and Epitope Structure. Immunity *48*, 855–871.

Landais, E., Huang, X., Havenar-Daughton, C., Murrell, B., Price, M.A., Wickramasinghe, L., Ramos, A., Bian, C.B., Simek, M., Allen, S., et al. (2016). Broadly Neutralizing Antibody Responses in a Large Longitudinal Sub-Saharan HIV Primary Infection Cohort. PLoS Pathog. *12*, e1005369.

Lee, J.H., Andrabi, R., Su, C.Y., Yasmeen, A., Julien, J.P., Kong, L., Wu, N.C., McBride, R., Sok, D., Pauthner, M., et al. (2017). A Broadly Neutralizing Antibody Targets the Dynamic HIV Envelope Trimer Apex via a Long, Rigidified, and Anionic β-Hairpin Structure. Immunity *46*, 690–702.

Lefranc, M.P., Giudicelli, V., Ginestoux, C., Jabado-Michaloud, J., Folch, G., Bellahcene, F., Wu, Y., Gemrot, E., Brochet, X., Lane, J., et al. (2009). IMGT, the international ImMunoGeneTics information system. Nucleic Acids Res. *37*, D1006–D1012.

Li, M., Gao, F., Mascola, J.R., Stamatatos, L., Polonis, V.R., Koutsoukos, M., Voss, G., Goepfert, P., Gilbert, P., Greene, K.M., et al. (2005). Human immunodeficiency virus type 1 env clones from acute and early subtype B infections for standardized assessments of vaccine-elicited neutralizing antibodies. J. Virol. *79*, 10108–10125.

Lu, C.L., Murakowski, D.K., Bournazos, S., Schoofs, T., Sarkar, D., Halper-Stromberg, A., Horwitz, J.A., Nogueira, L., Golijanin, J., Gazumyan, A., et al. (2016). Enhanced clearance of HIV-1-infected cells by broadly neutralizing antibodies against HIV-1 in vivo. Science *352*, 1001–1004.

Lynch, R.M., Boritz, E., Coates, E.E., DeZure, A., Madden, P., Costner, P., Enama, M.E., Plummer, S., Holman, L., Hendel, C.S., et al.; VRC 601 Study Team (2015). Virologic effects of broadly neutralizing antibody VRC01 administration during chronic HIV-1 infection. Sci. Transl. Med. *7*, 319ra206.

Mascola, J.R., Stiegler, G., VanCott, T.C., Katinger, H., Carpenter, C.B., Hanson, C.E., Beary, H., Hayes, D., Frankel, S.S., Birx, D.L., and Lewis, M.G. (2000). Protection of macaques against vaginal transmission of a

pathogenic HIV-1/SIV chimeric virus by passive infusion of neutralizing antibodies. Nat. Med. *6*, 207–210.

McCoy, A.J., Grosse-Kunstleve, R.W., Adams, P.D., Winn, M.D., Storoni, L.C., and Read, R.J. (2007). Phaser crystallographic software. J. Appl. Cryst. *40*, 658–674.

McCoy, L.E., and Burton, D.R. (2017). Identification and specificity of broadly neutralizing antibodies against HIV. Immunol. Rev. *275*, 11–20.

McGuire, A.T., Hoot, S., Dreyer, A.M., Lippy, A., Stuart, A., Cohen, K.W., Jardine, J., Menis, S., Scheid, J.F., West, A.P., et al. (2013). Engineering HIV envelope protein to activate germline B cell receptors of broadly neutralizing anti-CD4 binding site antibodies. J. Exp. Med. *210*, 655–663.

Mendoza, P., Gruell, H., Nogueira, L., Pai, J.A., Butler, A.L., Millard, K., Lehmann, C., Suárez, I., Oliveira, T.Y., Lorenzi, J.C.C., et al. (2018). Combination therapy with anti-HIV-1 antibodies maintains viral suppression. Nature *561*, 479–484.

Mikell, I., Sather, D.N., Kalams, S.A., Altfeld, M., Alter, G., and Stamatatos, L. (2011). Characteristics of the earliest cross-neutralizing antibody response to HIV-1. PLoS Pathog. *7*, e1001251.

Moore, J.P., Willey, R.L., Lewis, G.K., Robinson, J., and Sodroski, J. (1994). Immunological evidence for interactions between the first, second, and fifth conserved domains of the gp120 surface glycoprotein of human immunodeficiency virus type 1. J. Virol. *68*, 6836–6847.

Mouquet, H., Scharf, L., Euler, Z., Liu, Y., Eden, C., Scheid, J.F., Halper-Stromberg, A., Gnanapragasam, P.N., Spencer, D.I., Seaman, M.S., et al. (2012). Complex-type N-glycan recognition by potent broadly neutralizing HIV antibodies. Proc. Natl. Acad. Sci. USA *109*, E3268–E3277.

Olshevsky, U., Helseth, E., Furman, C., Li, J., Haseltine, W., and Sodroski, J. (1990). Identification of individual human immunodeficiency virus type 1 gp120 amino acids important for CD4 receptor binding. J. Virol. *64*, 5701–5707.

Pettersen, E.F., Goddard, T.D., Huang, C.C., Couch, G.S., Greenblatt, D.M., Meng, E.C., and Ferrin, T.E. (2004). UCSF Chimera–a visualization system for exploratory research and analysis. J. Comput. Chem. *25*, 1605–1612.

Plotkin, S.A. (2010). Correlates of protection induced by vaccination. Clin. Vaccine Immunol. *17*, 1055–1065.

Pugach, P., Ozorowski, G., Cupo, A., Ringe, R., Yasmeen, A., de Val, N., Derking, R., Kim, H.J., Korzun, J., Golabek, M., et al. (2015). A native-like SOSIP.664 trimer based on an HIV-1 subtype B env gene. J. Virol. *89*, 3380–3395.

Punjani, A., Rubinstein, J.L., Fleet, D.J., and Brubaker, M.A. (2017). cryoSPARC: algorithms for rapid unsupervised cryo-EM structure determination. Nat. Methods *14*, 290–296.

Raju, N., Setliff, I., and Georgiev, I.S. (2019). NFPws: a web server for delineating broadly neutralizing antibody specificities from serum HIV-1 neutralization data. Bioinformatics. Published online February 14, 2019. https://doi.org/10.1093/bioinformatics/btz097.

Rudicell, R.S., Kwon, Y.D., Ko, S.Y., Pegu, A., Louder, M.K., Georgiev, I.S., Wu, X., Zhu, J., Boyington, J.C., Chen, X., et al.; NISC Comparative Sequencing Program (2014). Enhanced potency of a broadly neutralizing HIV-1 antibody in vitro improves protection against lentiviral infection in vivo. J. Virol. *88*, 12669–12682.

Rusert, P., Kouyos, R.D., Kadelka, C., Ebner, H., Schanz, M., Huber, M., Braun, D.L., Hozé, N., Scherrer, A., Magnus, C., et al.; Swiss HIV Cohort Study (2016). Determinants of HIV-1 broadly neutralizing antibody induction. Nat. Med. *22*, 1260–1267.

Salazar-Gonzalez, J.F., Bailes, E., Pham, K.T., Salazar, M.G., Guffey, M.B., Keele, B.F., Derdeyn, C.A., Farmer, P., Hunter, E., Allen, S., et al. (2008). Deciphering human immunodeficiency virus type 1 transmission and early envelope diversification by single-genome amplification and sequencing. J. Virol. *82*, 3952–3970.

Sanders, R.W., Derking, R., Cupo, A., Julien, J.P., Yasmeen, A., de Val, N., Kim, H.J., Blattner, C., de la Peña, A.T., Korzun, J., et al. (2013). A next-generation cleaved, soluble HIV-1 Env trimer, BG505 SOSIP.664 gp140, expresses multiple epitopes for broadly neutralizing but not non-neutralizing antibodies. PLoS Pathog. *9*, e1003618.

Sarzotti-Kelsoe, M., Bailer, R.T., Turk, E., Lin, C.L., Bilska, M., Greene, K.M., Gao, H., Todd, C.A., Ozaki, D.A., Seaman, M.S., et al. (2014). Optimization and validation of the TZM-bl assay for standardized assessments of neutralizing antibodies against HIV-1. J. Immunol. Methods *409*, 131–146.

Sather, D.N., Armann, J., Ching, L.K., Mavrantoni, A., Sellhorn, G., Caldwell, Z., Yu, X., Wood, B., Self, S., Kalams, S., and Stamatatos, L. (2009). Factors associated with the development of cross-reactive neutralizing antibodies during human immunodeficiency virus type 1 infection. J. Virol. *83*, 757–769.

Scharf, L., Wang, H., Gao, H., Chen, S., McDowall, A.W., and Bjorkman, P.J. (2015). Broadly Neutralizing Antibody 8ANC195 Recognizes Closed and Open States of HIV-1 Env. Cell *162*, 1379–1390.

Scheid, J.F., Horwitz, J.A., Bar-On, Y., Kreider, E.F., Lu, C.L., Lorenzi, J.C., Feldmann, A., Braunschweig, M., Nogueira, L., Oliveira, T., et al. (2016). HIV-1 antibody 3BNC117 suppresses viral rebound in humans during treatment interruption. Nature *535*, 556–560.

Scheid, J.F., Mouquet, H., Feldhahn, N., Seaman, M.S., Velinzon, K., Pietzsch, J., Ott, R.G., Anthony, R.M., Zebroski, H., Hurley, A., et al. (2009a). Broad diversity of neutralizing antibodies isolated from memory B cells in HIV-infected individuals. Nature *458*, 636–640.

Scheid, J.F., Mouquet, H., Feldhahn, N., Walker, B.D., Pereyra, F., Cutrell, E., Seaman, M.S., Mascola, J.R., Wyatt, R.T., Wardemann, H., and Nussenzweig, M.C. (2009b). A method for identification of HIV gp140 binding memory B cells in human blood. J. Immunol. Methods *343*, 65–67.

Scheid, J.F., Mouquet, H., Ueberheide, B., Diskin, R., Klein, F., Olivera, T.Y., Pietzsch, J., Fenyo, D., Abadir, A., Velinzon, K., et al. (2011). Sequence and Structural Convergence of Broad and Potent HIV Antibodies That Mimic CD4 Binding. Science *333*, 1633–1637.

Scheres, S.H. (2012). RELION: implementation of a Bayesian approach to cryo-EM structure determination. J. Struct. Biol. *180*, 519–530.

Schoofs, T., Klein, F., Braunschweig, M., Kreider, E.F., Feldmann, A., Nogueira, L., Oliveira, T., Lorenzi, J.C., Parrish, E.H., Learn, G.H., et al. (2016). HIV-1 therapy with monoclonal antibody 3BNC117 elicits host immune responses against HIV-1. Science *352*, 997–1001.

Schrodinger, L.L.C. (2015). The PyMOL Molecular Graphics System, Version 1.8.

Seaman, M.S., Janes, H., Hawkins, N., Grandpre, L.E., Devoy, C., Giri, A., Coffey, R.T., Harris, L., Wood, B., Daniels, M.G., et al. (2010). Tiered categorization of a diverse panel of HIV-1 Env pseudoviruses for assessment of neutralizing antibodies. J. Virol. *84*, 1439–1452.

Shingai, M., Donau, O.K., Plishka, R.J., Buckler-White, A., Mascola, J.R., Nabel, G.J., Nason, M.C., Montefiori, D., Moldt, B., Poignard, P., et al. (2014). Passive transfer of modest titers of potent and broadly neutralizing anti-HIV monoclonal antibodies block SHIV infection in macaques. J. Exp. Med. *211*, 2061–2074.

Shingai, M., Nishimura, Y., Klein, F., Mouquet, H., Donau, O.K., Plishka, R., Buckler-White, A., Seaman, M., Piatak, M., Jr., Lifson, J.D., et al. (2013). Antibody-mediated immunotherapy of macaques chronically infected with SHIV suppresses viraemia. Nature *503*, 277–280.

Siciliano, B., and Khatib, O. (2008). Springer Handbook of Robotics (Springer).

Simek, M.D., Rida, W., Priddy, F.H., Pung, P., Carrow, E., Laufer, D.S., Lehrman, J.K., Boaz, M., Tarragona-Fiol, T., Miiro, G., et al. (2009). Human immunodeficiency virus type 1 elite neutralizers: individuals with broad and potent neutralizing activity identified by using a high-throughput neutralization assay together with an analytical selection algorithm. J. Virol. *83*, 7337–7348.

Sok, D., and Burton, D.R. (2018). Recent progress in broadly neutralizing antibodies to HIV. Nat. Immunol. *19*, 1179–1188.

Sok, D., van Gils, M.J., Pauthner, M., Julien, J.P., Saye-Francisco, K.L., Hsueh, J., Briney, B., Lee, J.H., Le, K.M., Lee, P.S., et al. (2014). Recombinant HIV envelope trimer selects for quaternary-dependent antibodies targeting the trimer apex. Proc. Natl. Acad. Sci. USA *111*, 17624–17629.

Stewart, J.J., Watts, P., and Litwin, S. (2001). An algorithm for mapping positively selected members of quasispecies-type viruses. BMC Bioinformatics *2*, 1.

Tiller, T., Meffre, E., Yurasov, S., Tsuiji, M., Nussenzweig, M.C., and Wardemann, H. (2008). Efficient generation of monoclonal antibodies from single human B cells by single cell RT-PCR and expression vector cloning. J. Immunol. Methods *329*, 112–124.

Tomaras, G.D., Binley, J.M., Gray, E.S., Crooks, E.T., Osawa, K., Moore, P.L., Tumba, N., Tong, T., Shen, X., Yates, N.L., et al. (2011). Polyclonal B cell responses to conserved neutralization epitopes in a subset of HIV-1-infected individuals. J. Virol. *85*, 11502–11519.

Traggiai, E., Chicha, L., Mazzucchelli, L., Bronz, L., Piffaretti, J.C., Lanzavecchia, A., and Manz, M.G. (2004). Development of a human adaptive immune system in cord blood cell-transplanted mice. Science *304*, 104–107.

Unni, S., Huang, Y., Hanson, R.M., Tobias, M., Krishnan, S., Li, W.W., Nielsen, J.E., and Baker, N.A. (2011). Web servers and services for electrostatics calculations with APBS and PDB2PQR. J. Comput. Chem. *32*, 1488–1491.

von Boehmer, L., Liu, C., Ackerman, S., Gitlin, A.D., Wang, Q., Gazumyan, A., and Nussenzweig, M.C. (2016). Sequencing and cloning of antigen-specific antibodies from mouse memory B cells. Nat. Protoc. *11*, 1908–1923.

Wagh, K., Bhattacharya, T., Williamson, C., Robles, A., Bayne, M., Garrity, J., Rist, M., Rademeyer, C., Yoon, H., Lapedes, A., et al. (2016). Optimal Combinations of Broadly Neutralizing Antibodies for Prevention and Treatment of HIV-1 Clade C Infection. PLoS Pathog. *12*, e1005520.

Walker, L.M., Phogat, S.K., Chan-Hui, P.Y., Wagner, D., Phung, P., Goss, J.L., Wrin, T., Simek, M.D., Fling, S., Mitcham, J.L., et al.; Protocol G Principal Investigators (2009). Broad and potent neutralizing antibodies from an African donor reveal a new HIV-1 vaccine target. Science *326*, 285–289.

Walker, L.M., Simek, M.D., Priddy, F., Gach, J.S., Wagner, D., Zwick, M.B., Phogat, S.K., Poignard, P., and Burton, D.R. (2010). A limited number of antibody specificities mediate broad and potent serum neutralization in selected HIV-1 infected individuals. PLoS Pathog. *6*, e1001028.

Ward, A.B., and Wilson, I.A. (2017). The HIV-1 envelope glycoprotein structure: nailing down a moving target. Immunol. Rev. *275*, 21–32.

West, A.P., Jr., Scharf, L., Horwitz, J., Klein, F., Nussenzweig, M.C., and Bjorkman, P.J. (2013). Computational analysis of anti-HIV-1 antibody neutralization panel data to identify potential functional epitope residues. Proc. Natl. Acad. Sci. USA *110*, 10598–10603.

West, A.P., Jr., Scharf, L., Scheid, J.F., Klein, F., Bjorkman, P.J., and Nussenzweig, M.C. (2014). Structural insights on the role of antibodies in HIV-1 vaccine and therapy. Cell *156*, 633–648.

Wibmer, C.K., Moore, P.L., and Morris, L. (2015). HIV broadly neutralizing antibody targets. Curr. Opin. HIV AIDS *10*, 135–143.

Winn, M.D., Ballard, C.C., Cowtan, K.D., Dodson, E.J., Emsley, P., Evans, P.R., Keegan, R.M., Krissinel, E.B., Leslie, A.G., McCoy, A., et al. (2011). Overview of the CCP4 suite and current developments. Acta Crystallogr. D Biol. Crystallogr. *67*, 235–242.

Wu, X., Yang, Z.Y., Li, Y., Hogerkorp, C.M., Schief, W.R., Seaman, M.S., Zhou, T., Schmidt, S.D., Wu, L., Xu, L., et al. (2010). Rational design of envelope identifies broadly neutralizing human monoclonal antibodies to HIV-1. Science *329*, 856–861.

Wyatt, R., Kwong, P.D., Desjardins, E., Sweet, R.W., Robinson, J., Hendrickson, W.A., and Sodroski, J.G. (1998). The antigenic structure of the HIV gp120 envelope glycoprotein. Nature *393*, 705–711.

Xu, L., Pegu, A., Rao, E., Doria-Rose, N., Beninga, J., McKee, K., Lord, D.M., Wei, R.R., Deng, G., Louder, M., et al. (2017). Trispecific broadly neutralizing HIV antibodies mediate potent SHIV protection in macaques. Science *358*, 85–90.

Yang, X., Farzan, M., Wyatt, R., and Sodroski, J. (2000). Characterization of stable, soluble trimers containing complete ectodomains of human immunodeficiency virus type 1 envelope glycoproteins. J. Virol. *74*, 5716–5725.

Ye, J., Ma, N., Madden, T.L., and Ostell, J.M. (2013). IgBLAST: an immunoglobulin variable domain sequence analysis tool. Nucleic Acids Res. *41*, W34-40.

Zhang, K. (2016). Gctf: Real-time CTF determination and correction. J. Struct. Biol. *193*, 1–12.

Zhang, Y., and Skolnick, J. (2005). TM-align: a protein structure alignment algorithm based on the TM-score. Nucleic Acids Res. *33*, 2302–2309.

Zhang, Y.J., Hatziioannou, T., Zang, T., Braaten, D., Luban, J., Goff, S.P., and Bieniasz, P.D. (2002). Envelope-dependent, cyclophilin-independent effects of glycosaminoglycans on human immunodeficiency virus type 1 attachment and infection. J. Virol. *76*, 6332–6343.

Zhou, T., Zheng, A., Baxa, U., Chuang, G.Y., Georgiev, I.S., Kong, R., O'Dell, S., Shahzad-Ul-Hussan, S., Shen, C.H., Tsybovsky, Y., et al. (2018). A Neutralizing Antibody Recognizing Primarily N-Linked Glycan Targets the Silent Face of the HIV Envelope. Immunity *48*, 500–513.

Zivanov, J., Nakane, T., Forsberg, B.O., Kimanius, D., Hagen, W.J., Lindahl, E., and Scheres, S.H. (2018). New tools for automated high-resolution cryo-EM structure determination in RELION-3. eLife *7*, e42166.

STAR★METHODS

KEY RESOURCES TABLE

REAGENT or RESOURCE	SOURCE	IDENTIFIER
Antibodies		
Monoclonal anti-HIV-1 Env SF5	Michel C. Nussenzweig, The Rockefeller University (This Paper)	N/A
Monoclonal anti-HIV-1 Env SF12	Michel C. Nussenzweig, The Rockefeller University (This Paper)	N/A
Monoclonal anti-HIV-1 Env 3BNC117	NIH AIDS Reagent Program	Cat# 12474
Monoclonal anti-HIV-1 Env 10-1074	NIH AIDS Reagent Program	Cat# 12477
Monoclonal anti-HIV-1 Env PGDM1400	Dennis R. Burton, Scripps; Sok et al., 2014	N/A
Monoclonal anti-HIV-1 Env 8ANC131	Michel C. Nussenzweig, The Rockefeller University; Scheid et al., 2011	N/A
Monoclonal anti-HIV-1 Env 8ANC195	Michel C. Nussenzweig, The Rockefeller University; Scheid et al., 2011	N/A
Monoclonal anti-HIV-1 Env PGT151	Dennis R. Burton, Scripps; Falkowska et al., 2014	N/A
Monoclonal anti-HIV-1 Env 35O22	NIH AIDS Reagent Program	Cat# 12586
Monoclonal anti-human IgM-PE-Cy5, Clone G20-127	BD Biosciences	Cat# 551079; RRID: AB_394036
Monoclonal anti-human IgD-FITC, Clone IA6-2	BD Biosciences	Cat# 555778; RRID: AB_396113
Monoclonal anti-human CD3-APC-Cy7, Clone SK7	BD Biosciences	Cat# 557832; RRID: AB_396890
Monoclonal anti-human CD19-PE-Cy7, Clone HIB19	BD Biosciences	Cat# 560728; RRID: AB_1727438
Monoclonal anti-human CD16-PB, Clone N/A	Mario Roederer, NIH	N/A
Monoclonal anti-human CD19-BV421, Clone HIB19	Biolegend	Cat# 302233; RRID: AB_10897802
Monoclonal anti-human CD20-BV421, Clone 2H7	Biolegend	Cat# 302329; RRID: AB_10933088
Monoclonal anti-human CD3-PerCP-Cy5.5, Clone OKT3	Biolegend	Cat# 317336; RRID: AB_2561628
Monoclonal anti-human CD14-PerCP-Cy5.5, Clone HCD14	Biolegend	Cat# 325622; RRID: AB_893250
Monoclonal anti-human CD335-PerCP-Cy5.5, Clone 9E2	Biolegend	Cat# 331920; RRID: AB_2561665
Monoclonal anti-human CD66b-PerCP-Cy5.5, Clone G10F5	Biolegend	Cat# 305108; RRID: AB_2077855
Monoclonal anti-human anti-human-IgM-BV605, Clone MHM-88	Biolegend	Cat# 314523; RRID: AB_2562373
Monoclonal anti-human anti-human-IgG-APC, Clone G18-145	BD Biosciences	Cat# 550931; RRID: AB_398478
Anti-6X His tag antibody	Abcam	Cat# ab9108; RRID: AB_307016
Goat Anti-Human IgG Fc, Multi-Species SP ads-HRP	Southern Biotech	Cat# 2014-05; RRID: AB_2795580
Peroxidase AffiniPure Goat Anti-Human IgG, Fcγ fragment specific	Jackson ImmunoResearch	Cat# 109-035-098; RRID: AB_2337586
Bacterial and Virus Strains		
f61 Panel of 20 HIV-1 Env-pseudotyped viruses for neutralization fingerprinting	Nicole Doria-Rose, NIH; Doria-Rose et al., 2017	N/A
Global Panel of 12 HIV-1 Env-pseudotyped viruses	NIH AIDS Reagent Program; deCamp et al., 2014	Cat# 12670
119 HIV-1 Env-pseudotyped viruses cross-clade panel	Michael S. Seaman, BIDMC; Freund et al., 2017; Mouquet et al., 2012	N/A
YU2 HIV-1 Env-pseudotyped viruses carrying mutations in common anti-HIV-1-mAb binding sites	Florian Klein, University of Cologne	N/A
Replication-competent HIV_{YU2} (YU2-envelope in pNL/HXB) for mouse experiment	Paul D. Bieniasz, The Rockefeller University; Zhang et al., 2002	N/A

(Continued on next page)

Continued

REAGENT or RESOURCE	SOURCE	IDENTIFIER
Biological Samples		
PBMCs from Donor 27845	M. Juliana McElrath, Fred Hutchinson Cancer Research Center	N/A
Plasma from Donor 27845	M. Juliana McElrath, Fred Hutchinson Cancer Research Center	N/A
Human cord blood/placental tissue (for isolation of Human CD34+ cells)	Department of Gynecology and Obstetrics, University Hospital of Cologne	N/A
Chemicals, Peptides, and Recombinant Proteins		
Dulbecco's Modified Eagle Medium (DMEM)	GIBCO	Cat# 11960-044
Fetal bovine serum (FBS)	Sigma-Aldrich	Cat# F9665
Penicillin/Streptomycin	GIBCO	Cat# 15140-122
Sodium Pyruvate	GIBCO	Cat# 11360-070
L-Glutamine	Thermo Fisher Scientific	Cat# 25030024
Gentamicin	Sigma-Aldrich	Cat# G1397-10ML
HEPES	Biochrom	Cat# L1613
Freestyle 293 Expression Medium	Thermo Fisher Scientific	Cat# 12338001
Interleukin 2 (IL-2)	Roche	Cat# 11147528001
Interleukin 21 (IL-21)	Life Technologies	Cat# PHC0211
Streptavidin-PE	BioLegend	Cat# 405203
BG505-SOSIP.664.Avi	John P. Moore, Weill Cornell Medical College; Sok et al., 2014	N/A
Superscript III Reverse Transcriptase	Thermo Fisher Scientific	Cat# 18080044
RNasin Plus RNase inhibitor	Promega	Cat# N2615
Random primers	Invitrogen	Cat# 48190-011
HotStarTaq DNA Polymerase	QIAGEN	Cat# 203203
Polyethylenimine (PEI), branched 25 kDa	Sigma	Cat# 408727
Protein G Sepharose 4 Fast Flow	GE Healthcare	Cat# 17-0618-05
BG505-SOSIP.664.His	John P. Moore, Weill Cornell Medical College; de Taeye et al., 2015; Sanders et al., 2013	N/A
YU2 gp120 monomer	John R. Mascola, NIH	N/A
YU2 gp120 monomer mutants (N160K, N332A, D368R)	Michel C. Nussenzweig, The Rockefeller University	N/A
YU2 gp140 foldon trimer	Richard Wyatt, The Scripps Research Institute; Yang et al., 2000	N/A
YU2 gp140 foldon trimer mutants (A281T+368K, N160K+A281T+368K)	Michel C. Nussenzweig, The Rockefeller University	N/A
ABTS 1-Step Solution	Thermo Fisher Scientific	Cat# 002024
Peroxidase Streptavidin	Jackson Immuno	Cat# 016-030-084
T4 DNA Polymerase	New England Biolabs	Cat# M0203L
Platinum Taq Green Hot Start	Thermo Fisher	Cat# 11966034
Fugene 6 Transfection Reagent	Promega	Cat# E2691
B41 SOSIP.664 v4.2	Pugach et al., 2015	N/A
SF12 Fab	Michel C. Nussenzweig, The Rockefeller University (This Paper)	N/A
10-1074 Fab	Mouquet et al., 2012	N/A
Critical Commercial Assays		
LIVE/DEAD Fixable Aqua Dead Cell Stain Kit	Invitrogen	Cat# L34957
TOPO TA cloning Kit	Thermo Fisher Scientific	Cat# K457501
BirA-500: BirA biotin-protein ligase standard reaction kit	Avidity	Cat# BirA500

(*Continued on next page*)

Continued

REAGENT or RESOURCE	SOURCE	IDENTIFIER
FluoReporter Mini-Biotin-XX Protein Labeling Kit	Thermo Fisher Scientific	Cat# F6347
QuikChange II XL Site-Directed Mutagenesis Kit	Agilent	Cat# 200521
Q5-Site-Directed Mutagenesis Kit	New England BioLabs	Cat# E0554S
NOVA Lite® HEp-2 ANA IgG (H&L) Immunoglobulin (External Evan's Blue)	Inova Diagnostics	Cat# 704230
Deposited Data		
Silent face antibody family nucleotide sequences	GenBank	GenBank: MK722158–MK722171
SF12–B41 SOSIP.664–10-1074 coordinates	PDB	PDB: 6OKP
SF12 Fab coordinates	PDB	PDB: 6OKQ
SF12–B41 SOSIP.664–10-1074 complex cryoEM maps (class 1 and class 2)	EMDB	EMDB: 20100, 20101
Experimental Models: Cell Lines		
Mouse: 3T3-msCD40L Cells	NIH AIDS Reagent Program	Cat# 12535
Human: HEK293EBNA1-6E (293-6E)	National Research Council Canada	NRC File 11565
Human: HEK293T	ATCC	Cat# CRL-11268
Human: HeLa-derived TZM-bl	NIH AIDS Reagent Program	Cat# 8129
CHO Flp-In^{TM} cells	Invitrogen	Cat# R75807
Experimental Models: Organisms/Strains		
NOD-Rag1^{null} IL2rg^{null} (NRG) mice	The Jackson Laboratory	Stock No. 007799
Oligonucleotides		
Human immunoglobulin variable region amplification primers for VH-, Vκ–, and Vλ	Michel C. Nussenzweig, The Rockefeller University; Scheid et al., 2011; Doria-Rose et al., 2015	N/A
HIV-1 qPCR Primer and Probe Set	Michel C. Nussenzweig, The Rockefeller University; Horwitz et al., 2017	N/A
HIV-1 YU2 env single genome sequencing primers	Michel C. Nussenzweig, The Rockefeller University; Horwitz et al., 2017	N/A
Recombinant DNA		
Human Expression vectors Igγ1, Igκ, Igλ, Ig-Fab heavy chain	Michel C. Nussenzweig, The Rockefeller University; Tiller et al., 2008	N/A
HIV-1$_{BG505.T332N}$ gp160 env expression plasmid	Rogier W. Sanders, Academic Medical Center, Netherlands	N/A
HIV-1$_{BG505.T332N}$ gp160 env expression plasmids carrying silent face antibody binding mutations	This Paper	N/A
HIV-1$_{YU2}$ Env gp160 env expression plasmid	Joseph Sodroski, Dana-Farber Cancer Institute	N/A
HIV-1 Env YU2 expression plasmid carrying silent face antibody binding mutations	This Paper	N/A
HIV-1 SG3 ΔEnv Non-infectious Molecular Clone (pSG3ΔEnv)	NIH AIDS Reagent Program	Cat# 11051
Software and Algorithms		
IgBLAST	National Library of Medicine; Ye et al., 2013	https://www.ncbi.nlm.nih.gov/igblast/
IMGT	International ImMunoGeneTics Information System; Lefranc et al., 2009	http://www.imgt.org
Geneious v8.1.9	Biomatters Ltd.	N/A
Prism 7	GraphPad	N/A
Pymol	Schrodinger, LLC 2015	RRID: SCR_000305
UCSF Chimera	Pettersen et al., 2004	https://www.cgl.ucsf.edu/chimera/

(*Continued on next page*)

Continued		
REAGENT or RESOURCE	SOURCE	IDENTIFIER
Phenix	Adams et al., 2010	https://www.phenix-online.org
Coot	Emsley and Cowtan, 2004	http://www2.mrc-lmb.cam.ac.uk/personal/pemsley/coot/
Relion	Scheres, 2012	https://www2.mrc-lmb.cam.ac.uk/relion/index.php?title=Main_Page
CCP4 suite	Winn et al., 2011	http://www.ccp4.ac.uk/index.php
XDS	Kabsch, 2010	http://xds.mpimf-heidelberg.mpg.de/
Antibody Database v2.0	Pamela J. Bjorkman, California Institute of Technology; West et al., 2013	N/A
Other		
HiLoad 16/600 Superdex 200 pg column	GE Healthcare	Cat# 28989335
2G12 5 ml column made in-house using using NHS-activated HP resin and 2G12 IgG	GE Healthcare	Cat# 17071601
Protein A column	GE Healthcare	Cat# 17040301
300 Mesh Quantifoil R2/2 copper grids	EM Resolutions	QR22300Cu25

CONTACT FOR REAGENT AND RESOURCE SHARING

Further information and requests for resources and reagents should be directed to and will be fulfilled by the Lead Contact, Pamela J. Bjorkman (bjorkman@caltech.edu). The Bjorkman laboratory cannot lawfully distribute clones in the pTT5 vector. Those wishing to obtain these clones must first obtain a license from the National Research Council of Canada.

EXPERIMENTAL MODEL AND SUBJECT DETAILS

Human subjects

Donor 27845 is an adult male who was diagnosed with HIV-1 in 1985. He was a study participant in Seattle Vaccine Unit Observational Protocols "Immune Determinants Favoring Non-Progression in HIV-1 Infection" and "Evaluation of HIV-Specific Immunological and Virological Responses of HIV-1 Multiply-Exposed Seronegative Individuals" (P.I. MJ McElrath) at the Fred Hutchinson Cancer Research Center and was followed from 1998 - 2006. Apart from an NIH interventional study during which Donor 27845 started and stopped anti-retroviral therapy (ART) from 1998-2001, the subject has been off ART. Samples for this study were obtained from 2005 and 2006. During the time of follow-up, viral loads ranged from 35 – 23,300 copies/ml (median: 1,640 copies/ml) and $CD4^+$ T cell counts ranged from 291 to 1,000 cells/mm^3 (median: 590 cells/mm^3). Studies and procedures were approved by the Fred Hutchinson Cancer Research Center Internal Review Board (FWA00001920). Samples for analysis were obtained under protocol MNU-0628 approved by the Rockefeller University Institutional Review Board.

Humanized mice

NOD-$Rag1^{null}$ $IL2rg^{null}$ (NRG) mice were purchased from The Jackson Laboratory, and were subsequently bred and maintained in the Dezentrales Tierhaltungsnetzwerk Weyertal at University of Cologne. NRG mice were fed ssniff complete feed 1124 during breeding and ssniff complete feed 1534 during maintenance, and kept under a 12 hr light/dark cycle with specific-pathogen-free (SPF) conditions. To determine the pharmacokinetics of antibody SF12, 6-week old non-humanized NRG mice (n = 3 per antibody, male and female mice in both groups) were injected intravenously via the tail-vein with 250 μg of antibody (SF12 or 3BNC117). Mice were bled on days 1, 3, 6, 9 and 14 after injection from the facial vein into Z-Gel Serum tubes (Sarstedt). Serum levels were determined using a previously described total IgG ELISA (Klein et al., 2012). Humanized mice for treatment experiments were generated using a previously described protocol with slight modifications (Klein et al., 2012; Traggiai et al., 2004). In brief, 1-5 days old NRG mice were sublethally irradiated, and 3-6 hours later injected intrahepatically with $CD34^+$ hematopoietic stem cells. CD34+ cells were enriched by magnetic bead-based positive selection (Miltenyi) from PBMCs obtained from human cord blood and by placental perfusion under a protocol approved by the ethics committee of the Medical Faculty of the University of Cologne (protocol #16-110). All cord blood and tissue donors provided written informed consent. Humanization screening was performed at 12 weeks post injection by flow cytometry as previously described (Klein et al., 2012). For treatment experiments, humanized mice were infected with HIV-1_{YU2} (Zhang et al., 2002) (produced in 293T cells) intraperitoneally (Horwitz et al., 2013; Klein et al., 2012). To determine viral loads, plasma viral RNA was measured using a quantitative PCR (qPCR) assay (Horwitz et al., 2013, 2017) based on pol using primers HIV-1 Pol region F 5′-TAATGGCAGCAATTTCACCA-3′ and HIV-1 Pol region R 5′-GAATGCCAAATTCCTGCTTGA-3′, and probe 5′-/56-FAM/CCCACCAAC/ZEN/ARGCRGCCTTAACTG/3IABkFQ/-3′. The limit of accuracy of

the assay (based on the standard curve used) was 384 copies/ml. Plasma viral loads were determined twice before experiment start and only mice with viral loads over 4,000 copies/ml were included in experiments. Male and female mice (18-67 weeks old) were used for treatment experiments. Treatment groups were matched primarily based on viral load and stem cell donor (previously identified as key determinants of viral load kinetics), with equal or comparable distributions of age and sex across groups. For antibody treatments, 1 mg of each antibody was administered subcutaneously as a loading dose followed by twice-weekly injections of 0.5 mg of each antibody in PBS subcutaneously for a total of 3 weeks (monotherapy) or 5 weeks (tri-mix). All mouse experiments were authorized by the State Agency for Nature, Environment and Consumer Protection (LANUV) of North Rhine-Westphalia.

Cell lines

HEK293T cells were obtained from the American Type Culture Collection (ATCC) and maintained in Dulbecco's modified Eagle Medium (DMEM, GIBCO) with 10% fetal bovine serum (FBS, Sigma Aldrich), 1x Penicillin/Streptomycin (GIBCO), 1 mM Sodium Pyruvate (GIBCO), 2 mM L-Glutamine (Thermo Fisher Scientific) at 37°C/5% CO_2. The HeLa-derived TZM-bl reporter cell line was sourced from the NIH AIDS Reagent Program and maintained in DMEM containing 10% fetal bovine serum, 1 mM Sodium Pyruvate, 2 mM L-Glutamine (Thermo Fisher Scientific), 50 μg/ml Gentamicin (Sigma-Aldrich), and 25 mM HEPES (Biochrom) at 37°C/5% CO_2. HEK293EBNA1-6E (293-6E) cells were obtained from the National Research Council Canada (NRC) and maintained in Freestyle 293 Expression Medium (Thermo Fisher Scientific) containing 0.2% Penicillin/Streptomycin at 37°C /5% CO_2 with shaking at 90-120 rpm. The sex of these cell lines is unknown. CHO Flp-In^{TM} cells (Invitrogen) were a kind gift from the lab of John Moore (Cornell University) and maintained in Ham's F-12 Medium supplemented with 10% heat-inactivated FBS (Sigma-Aldrich), 200 U/ml penicillin/streptomycin, 2 mM L-glutamine, 20 mM HEPES, 0.1 mM non-essential amino acids, 1 mM sodium pyruvate (GIBCO), and further supplemented with 100 μg/ml Zeocin (Invitrogen).

METHODS DETAILS

IgG isolation for polyclonal IgG neutralization testing

IgG from subject 27845 was purified from heat-inactivated (1h 56°C) plasma (late 2005 time point) using Protein G Sepharose 4 Fast Flow (GE Healthcare), buffer exchanged into phosphate buffered saline (PBS) using an Amicon Ultra 30 kDa (Millipore), and sterile-filtered.

Neutralization fingerprinting analysis

Neutralization fingerprinting (Figure 1B) of the polyclonal antibody response of subject 27845 was done using a panel of 20 diverse HIV-1 strains (Doria-Rose et al., 2017). In brief, the neutralization fingerprint of a serum/polyclonal IgG (the potency-defined pattern of neutralization of a set of diverse viral strains) is represented as a combination of the neutralization fingerprints of a reference set of bNAbs, grouped in ten epitope-specific clusters. Using this method, the prevalence of each of the ten antibody groups can be estimated for the given serum/polyclonal IgG, with prevalence scores ranging between 0 (low) and 1 (high). Additionally, two measures (Residual score and Median of scores) are computed as a way to estimate prediction confidence for the prevalence scores (Georgiev et al., 2013; Raju et al., 2019). For neutralization fingerprinting of monoclonal antibodies (Figure 1F), a set of 80 viruses for which data was available for all antibodies was used. The tree was constructed with a distance metric based on the similarity of the neutralization patterns of the different antibodies. First, the correlations between the neutralization fingerprints (the antibody-specific pattern of neutralization of a set of diverse HIV-1 strains) were computed for each pair of antibodies. The antibody-antibody correlation matrix was then used as input to a hierarchical clustering algorithm to generate a neutralization fingerprinting-based antibody tree. Generally, antibodies that cluster closely together in the tree may indicate similar patterns of neutralization sensitivity/resistance for the given set of strains.

B cell microculture

Sorting and culturing of memory B cells was performed according to a previously published protocol (Doria-Rose et al., 2015; Huang et al., 2013). In brief, peripheral blood mononuclear cells (PBMCs) were stained with LIVE/DEAD Fixable Aqua (Invitrogen), CD19-PE-Cy7, CD16-Pacific Blue, CD3-APC-Cy7, IgM-PE-Cy5 and IgD-FITC. Gating was done on IgM$^-$ and IgD$^-$ negative B cells, and these were bulk sorted using a FACS Aria II cytometer. Bulk B cells were then diluted and plated at 2 B cells per well in 384-well plates. B cells were cultured for two weeks in the presence of IL-2 (Roche), IL-21 (Life Technologies) and CD40L-expressing NIH 3T3 cells as described (Doria-Rose et al., 2015; Huang et al., 2013). To assess culture success, a total IgG ELISA was performed on supernatants after two weeks of culture to determine the number of wells with positive IgG production. Cell supernatants were then screened in a microneutralization TZM.bl assay against viruses BaL.26 and BG505.T332N. Wells with neutralization > 50% against one or both strains were amplified using various sets of previously described primer sets for heavy chain and light chain (Doria-Rose et al., 2015; Scheid et al., 2011). Clone specific primers in the leader region were designed when necessary to obtain fully native sequences of the entire framework 1 region (FWR1). Positive bands were sanger sequenced using reverse amplification primers. In the case that multiple B cells were present (double peaks were obtained), bands were subcloned using the TOPO-TA kit (Invitrogen), colony PCR was performed, and bands were again sequenced by Sanger sequencing. Antibody sequences were analyzed using both IgBLAST and the international ImMunoGeneTics information system (IMGT) (Lefranc et al., 2009; Ye et al., 2013). Obtained heavy and light chain genes were cloned into human Igγ1-, Igκ or Igλ-expression vectors using sequence and

ligation independent cloning (SLIC) (Jeong et al., 2012; Tiller et al., 2008; von Boehmer et al., 2016). The correct heavy and light chain pairing of the SF5/SF12 antibody family was confirmed by single B cell BG505 bait sorting data.

Single B cell bait-sorting

BG505 SOSIP.664-Avi for B cell sorting was produced in CHO cells and purified using a PGT145 immunoaffinity column as described (Pugach et al., 2015; Sok et al., 2014). Biotinylation of BG505 was done using BirA-ligase (Avidity) according to the manufacturer's instructions. An aliquot of BG505 SOSIP.664-Avi-biotin was freshly coupled to Streptavidin-PE (Invitrogen) using 2.5 μg and 1 μl (0.2 mg/ml) of Streptavidin-PE in a total volume of 10 ul PBS. For the sort, 20 million PBMCs were freshly thawed and stained with the following fluorophore-coupled anti-human antibodies: IgG-APC, IgM-BV605, CD19-BV421, CD20-BV421, CD3-PerCP-Cy5.5, CD14 PerCP-Cy5.5, CD335 PerCP-Cy5.5, CD606 PerCP-Cy5.5 and 1:20 Streptavidin-PE coupled BG505.SOSIP.664 mix described above. Staining was performed for 30 mins at 4°C. Sorting was done on a FACS Aria II. The gating first included singlets, followed by exclusion of unwanted cells ($CD3^-$, $CD14^-$, $CD335^-$, $CD606^-$), selection for B cells ($CD19^+$, $CD20^+$) and finally sorting of single IgG^+ PE^+ cells into 96-well plates containing lysis buffer. B cell antibody genes were amplified and Sanger sequenced. Antibody sequences were analyzed using both IgBLAST and the international ImMunoGeneTics information system (IMGT) (Lefranc et al., 2009; Ye et al., 2013). Sequences of interest were cloned into human Igγ1-, Igκ or Igλ-expression vectors by SLIC as described above.

Phylogenetic analysis of SF family heavy chain sequences

The IgV_H4*59*01 *Homo sapiens* allele sequence was obtained from the international ImMunoGeneTics information system (IMGT) (Lefranc et al., 2009). Antibody heavy chain nucleotide sequences of the SF family were aligned with the IgV_H4*59*01 sequence in Geneious R8 (v8.1.9) using ClustalW. The maximum-likelihood tree was generated using the RAxML plugin (v 7.2.8) with a GTR Gamma model using the 'Rapid Bootstrapping and search for best-scoring ML tree' function with 100 bootstrap replicates. The best-scoring ML tree was then formatted using FigTree (v1.4.3).

Antibody production for ELISA, neutralization assays and *in vivo* experiments

293-6E cells were maintained in Freestyle 293 Expression Medium (Thermo Fisher Scientific) containing 0.2% Penicillin-Streptomycin (Thermo Fisher Scientific). Paired heavy and light chain expression constructs were transfected into 293-6E cells (NRC) using branched polyethylenimine (PEI) 25 kDA (Sigma). After 7 days of culture, cells were spun down at 4200 g for 40 mins at 4°C and supernatants were filtered through 0.22 μM aPES (Thermo Nalgene Rapid-Flow). Antibodies were then purified from filtered supernatants using Protein G Sepharose 4 Fast Flow (GE Healthcare) according to standard protocols. Antibodies were buffer exchanged and concentrated into PBS using Amicon Ultra centrifugal filter (Millipore) with either a 30 or 50 kDA molecular weight cutoff (MWCO).

Enzyme-linked immunosorbent assay (ELISA) of (mutant) YU2 gp120/gp140 proteins

Wild-type and mutant His-tagged YU2 gp120/gp140 proteins were expressed by transient transfection of 293-6E cells and purified using Ni-NTA according to manufacturer's instructions. Corning Costar 96-Well Assay high-binding plates were coated for 1h at 37°C with 2 μg/ml of the respective protein (YU2 gp120 WT, YU2 gp120 $D368R_{gp120}$, YU2 gp120 $N332A_{gp120}$, YU2 gp120 $N160K_{gp120}$ and YU2 gp140 WT, YU2 gp140 $A281T_{gp120}$/$D368K_{gp120}$, YU2 gp140 $N160K_{gp120}$ $A281T_{gp120}$/$D368K_{gp120}$ and $N332K_{gp120}$ (triple mutant)) using a volume of 50 μl/well. Plates were washed 6x using PBS-Tween20 (0.05%), and subsequently blocked using 3% BSA in PBS for 1h at 37°C (200 μl/well). After washing, serially-diluted antibodies were added (starting at 4 or 10 ug/ml, 1:3 dilution series) at 50 μl/well in 1% PBS/BSA and incubated for 1h at room temperature or 37°C. After another wash step, anti-human IgG (Southern Biotech or Jackson Immunoresearch) was added at 1:5000 (50 μl/well) in 1% PBS/BSA for 30 mins at 37°C. Development was done using 100 μl/well ABTS 1-Step Solution (Thermo Fisher Scientific), and absorbance was measured at 405 nm on a FluoStar Omega or 415 nm on a Tecan Sunrise.

BG505 SOSIP.664-His ELISAs

Corning Costar 96-Well Assay high-binding plates were coated overnight at room temperature or for 1h at 37°C with 2 μg/ml anti-His-tag antibody (Abcam) in PBS (50 μl/well). Plates were washed 6x using PBS-Tween20 (0.05%), and subsequently blocked using 3% BSA in PBS or 2% milk powder in PBS for 1h at 37°C (200 μl/well). After washing, purified BG505 SOSIP.664-His (de Taeye et al., 2015; Sanders et al., 2013) was added at 2 μg/ml in 1% BSA in PBS (50 μl/well), and incubated for 1h at 37°C, followed by another washing step. Next, serially-diluted antibodies were added (starting at 4 or 10 ug/ml, 1:3 dilution series) at 50 μl/well in 1% PBS/BSA, and incubated for 1h at room temperature or 37°C. After washing, anti-human IgG (Southern Biotech) was added at 1:5000 in 1% BSA in PBS (50 μl/well) for 30 mins at 37°C. Post washing, development was done using 100 μl/well ABTS 1-Step Solution (Thermo Fisher Scientific), and absorbance was measured at 405 nm on a FluoStar Omega or 415 nm on a Tecan Sunrise.

Competition ELISAs

Antibodies SF5 and SF12 were biotinylated using the FluoReporter Mini-Biotin-XX Protein Labeling Kit (Thermo Fisher Scientific). Corning Costar 96-Well Assay high-binding plates were coated overnight at room temperature or for 1h at 37°C with 2 μg/ml anti-His-tag antibody (Abcam) in PBS (50 μl/well). Plates were washed 6x using PBS-Tween20 (0.05%), and subsequently blocked using 3% BSA in PBS for 1h at 37°C (200 μl/well). After washing, BG505 SOSIP.664 was added at 2 μg/ml in 1% BSA in PBS (50 μl/well),

and incubated for 1h at 37°C, followed by another washing step. Next, serially-diluted competitor antibodies (starting at 32 μg/ml, 1:3 dilution series) were added at 50 μl/well in 1% PBS/BSA and incubated for 1h at room temperature. Plates were washed and biotinylated SF5 or SF12 were added at 0.5 μg/ml (50 μl/well in 1% PBS/BSA) and incubated for 1h room temperature. After another wash step, Streptavidin-HRP (1:1000) was added at 50 μl/well in 1% PBS/BSA for 30 mins at room temperature. Development was done using 100 μl/well ABTS 1-Step Solution (Thermo Fisher Scientific), and absorbance was measured at 405 nm on a FluoStar Omega or 415 nm on a Tecan Sunrise.

Generation of mutant HIV_{YU2} and HIV_{BG505} pseudoviruses

Point mutations were introduced into the HIV_{YU2} and $HIV_{BG505T332N}$ gp160 expression plasmids using the QuikChange site-directed mutagenesis kit (Agilent Technologies) or the Q5 Site-directed mutagenesis kit (NEB) according to manufacturer's specifications. Pseudoviruses were produced by co-transfection with pSG3ΔEnv into HEK293T according to an established protocol (Sarzotti-Kelsoe et al., 2014).

In vitro neutralization assays

Neutralization activities of polyclonal IgG and monoclonal antibodies were determined using a luciferase-based TZM.bl assay (Li et al., 2005; Sarzotti-Kelsoe et al., 2014; Seaman et al., 2010), which measures the reduction of Tat-induced luciferase expression in TZM-bl reporter cells during a single round of infection. Samples were assayed at least in in duplicate. Polyclonal IgG neutralization assays were done using a starting concentration of 500 μg/ml and monoclonal antibodies were assayed at starting concentrations of 10, 25 or 50 μg/ml. IC_{50}s and IC_{80}s were derived 5-parameter curve fitting. Neutralization was also assessed against murine leukemia virus (MuLV) to detect unspecific activity (Sarzotti-Kelsoe et al., 2014). Neutralization data for Figures 1E and 2E were analyzed and graphed using Antibody Database (v 2.0) (West et al., 2013).

Autoreactivity and polyreactivity assays

Autoreactivity of antibodies SF5 and SF12 and reference antibodies 4E10 and 2F5 was determined using the commercially-available HEp-2 based assay NOVA Lite kit (Inova Diagnostics) at an IgG concentration of 25 μg/ml. Slides were photographed on a Leica DMI 6000 B with an exposure of 800 ms, Gain of 10 and Intensity of 100%. Measurements were done in duplicate. Representative images are shown in Figure S1.

Polyreactivity assays were conducted using ELISA detection of non-specific binding to baculovirus extracts as described (Hötzel et al., 2012). Briefly, a solution of 1% baculovirus particles in 100mM sodium bicarbonate buffer pH 9.6 was absorbed onto the wells of a 384-well ELISA plate (Nunc Maxisorp) using a Tecan Freedom Evo liquid handling robot, and the plate was incubated overnight at 4°C. The plate was then blocked with 0.5% BSA in PBS for 1 hour at room temperature. Purified IgGs (diluted to 1 μg/mL in PBS, 0.5% BSA) were added to the blocked assay plate and incubated for 3 hours at room temperature. Bound IgG was detected as the luminescence signal at 425 nm using an HRP-conjugated anti-human IgG (H&L) secondary antibody (Genscript) and SuperSignal ELISA Femto Maximum Sensitivity Substrate (Thermo Fisher Scientific).

Single genome sequencing of plasma HIV-1 Env genes

Amplification of genes encoding HIV-1 Env gp160 from single viral genomes was carried out as described previously with slight modifications (Keele et al., 2008; Salazar-Gonzalez et al., 2008). In brief, complementary DNA (cDNA) was synthesized using primer YB383 5′-TTTTTTTTTTTTTTTTTTTTTTTTRAAGCAC-3′ and Superscript III (Invitrogen) according to manufacturer's instructions. cDNA was then serially diluted and HIV-1 Env was amplified in two rounds of nested PCR using Platinum Taq Green Hot Start (Thermo Fisher Scientific) and primers specifically adapted for $HIV_{YU2NL4-3}$ (1st round primers were YB383 and YB50 5′- GGCTTAGGCAT CTCCTATGGCAGGAAGAA-3′ ; 2nd round primers YB49 5′-TAGAAAGAGCAGAAGACAGTGGCAATGA-3′, YB52 5′-GGTGTG TAGTTCTGCCAATCAGGGAAGWAGCCTTGTG-3′). Positive Bands from amplifications with less than 30% efficiency were PCR-purified using the Nucleospin Gel and PCR-Clean Up kit (Macherey Nagel) and Sanger sequenced using a set of 8 primers. Env sequences were assembled using the Geneious 8.1.9 (Biomatters) de-novo assembly tool. Assembled sequences were cross-checked against sequencing traces again to validate assemblies, and sequences with full coverage of gp160 Env were used in downstream analyses.

Protein expression and purification for structural studies

Fabs from SF12, SF5 and 10-1074 IgGs were produced as described (Scharf et al., 2015). Briefly, Fabs were expressed by transiently transfecting HEK293-6E cells with vectors encoding the appropriate light chain and C-terminal 6x-His tagged heavy chain genes. Secreted Fabs were purified from cell supernatants using Ni^{2+}-NTA affinity chromatography (GE Healthcare), followed by size exclusion chromatography (SEC) with a Superdex200 16/60 column (GE Healthcare). Purified Fabs were concentrated and maintained at 4°C in storage buffer (20 mM Tris pH 8.0, 150 mM NaCl, 0.02% sodium azide).

A gene encoding soluble B41 SOSIP.664 gp140 trimer, including $SF501C_{gp120}$, $T605C_{gp41}$, and $I559P_{gp41}$ substitutions, an enhanced gp120–gp41 cleavage site (REKR to RRRRRR), and a stop codon after residue 664_{gp41} (Env numbering according to HXB2 nomenclature), was stably expressed in Chinese hamster ovary cells as described (Chung et al., 2014; Pugach et al., 2015). Secreted Env trimers expressed in the absence of glycosylation inhibitors were isolated from cell supernatants using 2G12 immunoaffinity chromatography by covalently coupling 2G12 IgG monomer to an activated-NHS Sepharaose column (GE Healthcare) as described (Scharf et al., 2015).

Trimers were eluted using 3M $MgCl_2$ and immediately dialyzed into storage buffer before SEC purification with a Superdex200 16/60 column (GE Healthcare) against the same buffer. Peak fractions pertaining to SOSIP trimers were pooled and repurified using the same column and buffer conditions. Individual fractions were stored separately at 4°C.

Crystal structure of SF12 Fab

Purified Fab was concentrated to 10-15 mg/mL by centrifugation with a 30-kDa concentrator (Amicon). Initial matrix crystallization trials were performed at room temperature using the sitting drop vapor diffusion method by mixing equal volumes of protein sample and reservoir using a TTP LabTech Mosquito robot and commercially-available screens (Hampton Research and QIAGEN). Initial hits were optimized and crystals were obtained in 0.1 M HEPES pH 7.0, 1.6 M Sodium Formate at 20°C. Crystals were cryo-protected stepwise to 3.0 M Sodium Formate before being cryopreserved in liquid nitrogen.

X-ray diffraction data were collected for SF12 Fab at the Stanford Synchroton Radiation Lightsource (SSRL) beamline 12-2 on a Pilatus 6M pixel detector (Dectris). Data from a single crystal were indexed and integrated in XDS (Kabsch, 2010) and merged with AIMLESS in the *CCP4* software suite (Winn et al., 2011). Structures were determined by molecular replacement in PHASER (McCoy et al., 2007) using a single search with coordinates of the F105 Fab (PDB 1U6A), which had ~85% sequence identity to SF12 after removal of CDR loops. Models were refined using B-factor refinement in CNS (Brunger, 2007) and Phenix (Adams et al., 2010), followed by several cycles of manual building with B factor sharpening in Coot (Emsley et al., 2010).

Cryo-EM sample preparation

Complexes of B41-SF12-101074 were assembled by incubating purified SF12 Fab with B41 SOSIP.664 trimer at a 1.2:1 Fab:gp120-protomer molar ratio. Following overnight incubation at RT, 10-1074 Fab was incubated with the complex at a 1.2:1 Fab:gp120-protomer molar ratio for 5 h. SF12–B41–10-1074 complexes were diluted to 0.5-0.8 mg/ml in TBS and 3μL was added to Quantifoil R2/2 300 mesh copper grids (Electron Microscopy Services) that had been freshly glow-discharged using a PELCO easiGlow (Ted Pella). Samples were immediately vitrified in 100% liquid ethane using a Mark IV Virtoblot (Thermo Fisher Scientific) by blotting for 2.5-4 s with Whatman No. 1 filter paper at 20°C and 100% relative humidity.

Cryo-EM data collection and processing

Single-particle cryo-EM data were collected on a Titan Krios transmission electron microscope (Thermo Fisher Scientific) operating at 300 kV, using the EPU automated image acquisition software (Thermo Fisher Scientific). Movies were collected on a Gatan K2 Summit direct electon detector (DED) operating in counting mode at a nominal magnification of 130,000x (1.09 Å/pixel) using a defocus range of −1.2 μm to −3.0 μm. Movies were collected over an 8 s exposure with an exposure rate of ~4.8 e^-/pixel/s, resulting in a total dose of ~40 $e^-/Å^2$.

Movies were motion corrected and doseweighted using the MotionCor2 frame alignment program in RELION-3 (Zivanov et al., 2018). Non-doseweighted summed images were used for CTF determination using Gctf (Zhang, 2016), and reference-free particle picking from 18 micrographs was achieved using Laplacian-of-Gaussian filtering in RELION-3 (Zivanov et al., 2018). An initial stack of 1,907 particles was 2D classified and the best classes representing top-down and side views of Env-trimers was used for subsequent automated template-based picking in RELION-3. 676,161 particles were extracted from 2,209 dose-weighted micrographs, binned 4x4 (4.36 Å/pixel), and subjected to reference-free 2D classification in RELION-3 and a 240 Å circular mask. A total of 371,665 particles corresponding to class averages that displayed secondary-structural elements and represented views different views of Fab bound Env-trimer were extracted and re-centered prior to heterogenous ab inito model generation using cryoSPARC v2.2 (Punjani et al., 2017).

The generated volume was low-passed filtered to 40 Å and used as an initial model for 3D auto-refinement in RELION-3. Due to the observed low occupancy of 10-1074 Fab, particles were re-extracted unbinned (1.09 Å/pixel) and 3D classified (C1 symmetry, k = 8) with a soft mask generated from the initial model (5-pixel extension, 10-pixel soft cosine edge). Classification resulted in two distinct classes comprising three or two SF12 Fabs bound per trimer and one 10-1074 Fab bound per trimer. Particles from each class were then separately refined, followed by 3D auto-refinement using a soft mask in which Fab constant domains were masked out. Class 1 (301,920 particles) refined to a final estimaled resolution of ~3.28 Å ($SF12_3$—B41–$10\text{-}1074_1$; C1 symmetry) and class 2 (55,136 particles) refined to a final estimated resolution of ~4.36 Å ($SF12_2$—B41–$10\text{-}1074_1$; C1 symmetry) according to gold-standard FSC (Bell et al., 2016).

Modeling and refinement of cryo-EM structures

For the final reconstruction of class 1 ($SF12_3$—B41–$10\text{-}1074_1$; C1 symmetry), initial coordinates were generated by docking reference models into the cryo-EM density using UCSF Chimera v1.13 (Goddard et al., 2007) (gp120-gp41, PDB 6CH9; 10-1074 Fab, PDB 4FQQ; SF12 Fab, this work). Initial models were then refined into the EM maps using one round of rigid body, morphing, and simulated annealing followed by subsequent rounds of B-factor refinement in Phenix (Adams et al., 2010). Models were manually built following iterative rounds of real-space and B-factor refinement in Coot (Emsley et al., 2010) and Phenix (Adams et al., 2010) with secondary structure restraints. Modeling of glycans was achieved by interpreting cryo-EM density at PNGS in Coot using a map with a −75 $Å^2$ B-factor sharpening value, contoured at 6σ due to the lower resolution of glycans at the periphery of the structure. Validation of model coordinates was performed using MolProbity (Chen et al., 2010) and Privateer (Agirre et al., 2015).

Structural and bioinformatic analyses

Superpositions and figures were rendered using PyMOL (Version 1.5.0.4 Schrodinger, LLC), and protein electrostatic calculations were done using APBS and PDB2PQR webservers (Unni et al., 2011). Buried surface areas (BSAs) were determined with PDBePISA using a 1.4Å probe (Krissinel and Henrick, 2007). Potential hydrogen bonds were assigned using a distance of < 3.6Å and an A-D-H angle of > 90°, while the maximum distance allowed for a van der Waals interaction was 4.0Å. Putative H-bonds, van der Waals assignments and total BSA should be considered tentative, owing to the relatively low structure resolutions. Computational analysis of neutralization panel data (Table S7) was done as previously described (West et al., 2013). For determining the difference in orientation of the antibody variable domains of the SF12-Env and VRC-PG05-Env complexes, those structures were aligned on gp120, and then the transformation relating the V_H-V_L domains was calculated by using TM-align (Zhang and Skolnick, 2005). The corresponding screw transformation was calculated as described (Siciliano and Khatib, 2008) and visualized using Antibody Database (West et al., 2013).

DATA AND SOFTWARE AVAILABILITY

The accession numbers for the nucleotide sequences of SF-family members are GenBank: MK722158–MK722171. The accession numbers for the cryo-EM reconstructions of the SF12–B41–10-1074 complexes comprising three or two SF12 Fabs are Electron Microscopy Data Bank (EMDB): EMD-20100 and EMD-20101, respectively. The accession numbers for coordinates for atomic models of the cryo-EM SF12–B41–10-1074 complex (class 1: three SF12 Fabs and one 10-1074 Fab) and the unliganded SF12 Fab crystal structure are Protein Data Bank (PDB): PDB 6OKP and PDB 6OKQ, respectively.

Immunity, Volume *50*

Supplemental Information

Broad and Potent Neutralizing Antibodies

Recognize the Silent Face of the HIV Envelope

Till Schoofs, Christopher O. Barnes, Nina Suh-Toma, Jovana Golijanin, Philipp Schommers, Henning Gruell, Anthony P. West Jr., Franziska Bach, Yu Erica Lee, Lilian Nogueira, Ivelin S. Georgiev, Robert T. Bailer, Julie Czartoski, John R. Mascola, Michael S. Seaman, M. Juliana McElrath, Nicole A. Doria-Rose, Florian Klein, Michel C. Nussenzweig, and Pamela J. Bjorkman

Supplementary Figures

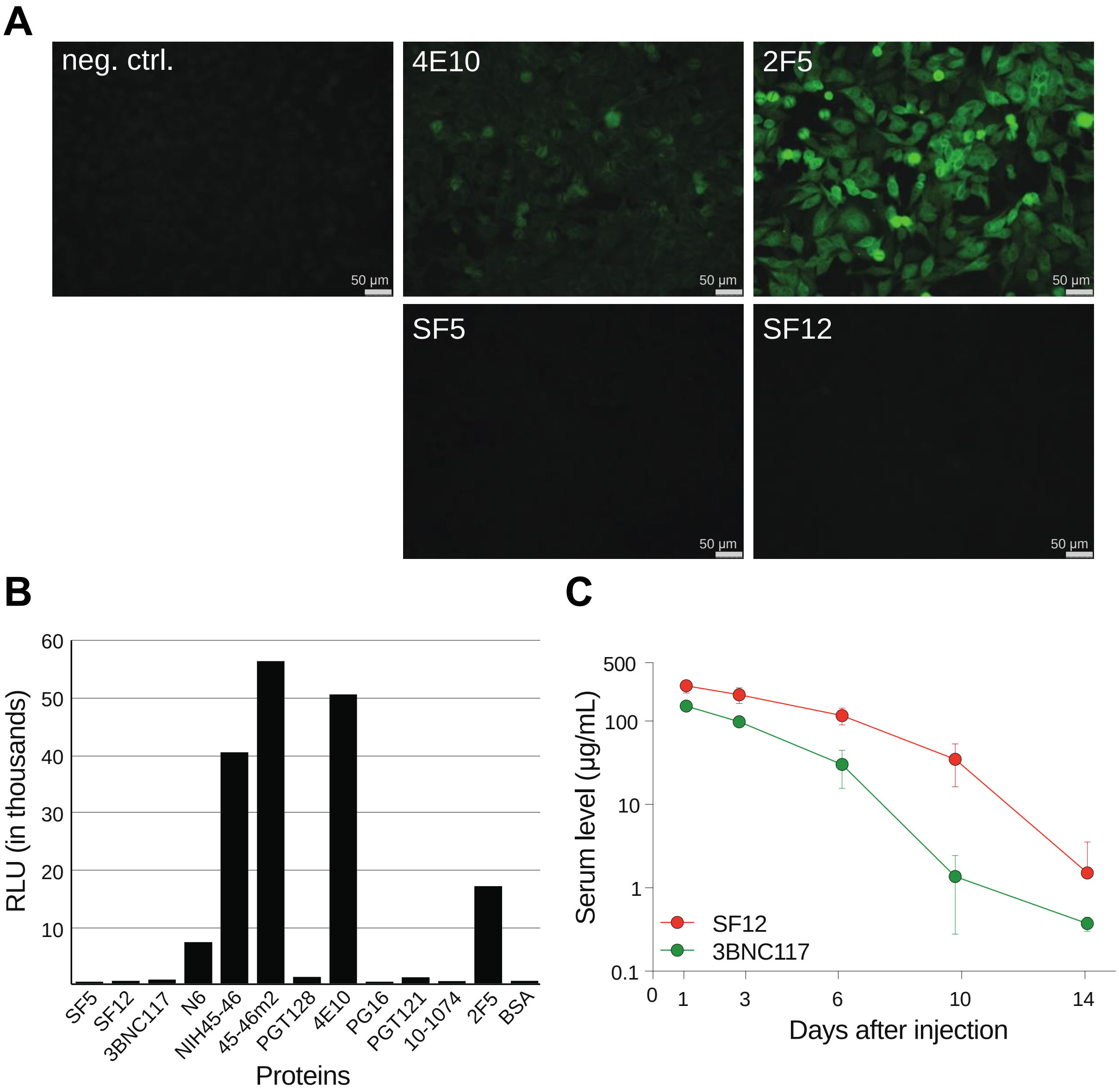

Figure S1. Polyreactivity and pharmacokinetics assays of SF12 and SF5 antibodies. Related to Figure 1. (A) HEp-2 assay for potential autoreactivity of indicated bNAbs. Data representative of 2 repeat assays. (B) Polyreactivity ELISA-based assay detecting non-specific binding of a panel of bNAbs and a control protein (BSA) to a baculovirus extract. (C) *In vivo* pharmacokinetics of SF12 IgG compared with 3BNC117 IgG measured in 6-week old non-reconstituted NRG mice (3 mice each antibody). One independent experiment. Shown is Mean of all 3 mice ± SD.

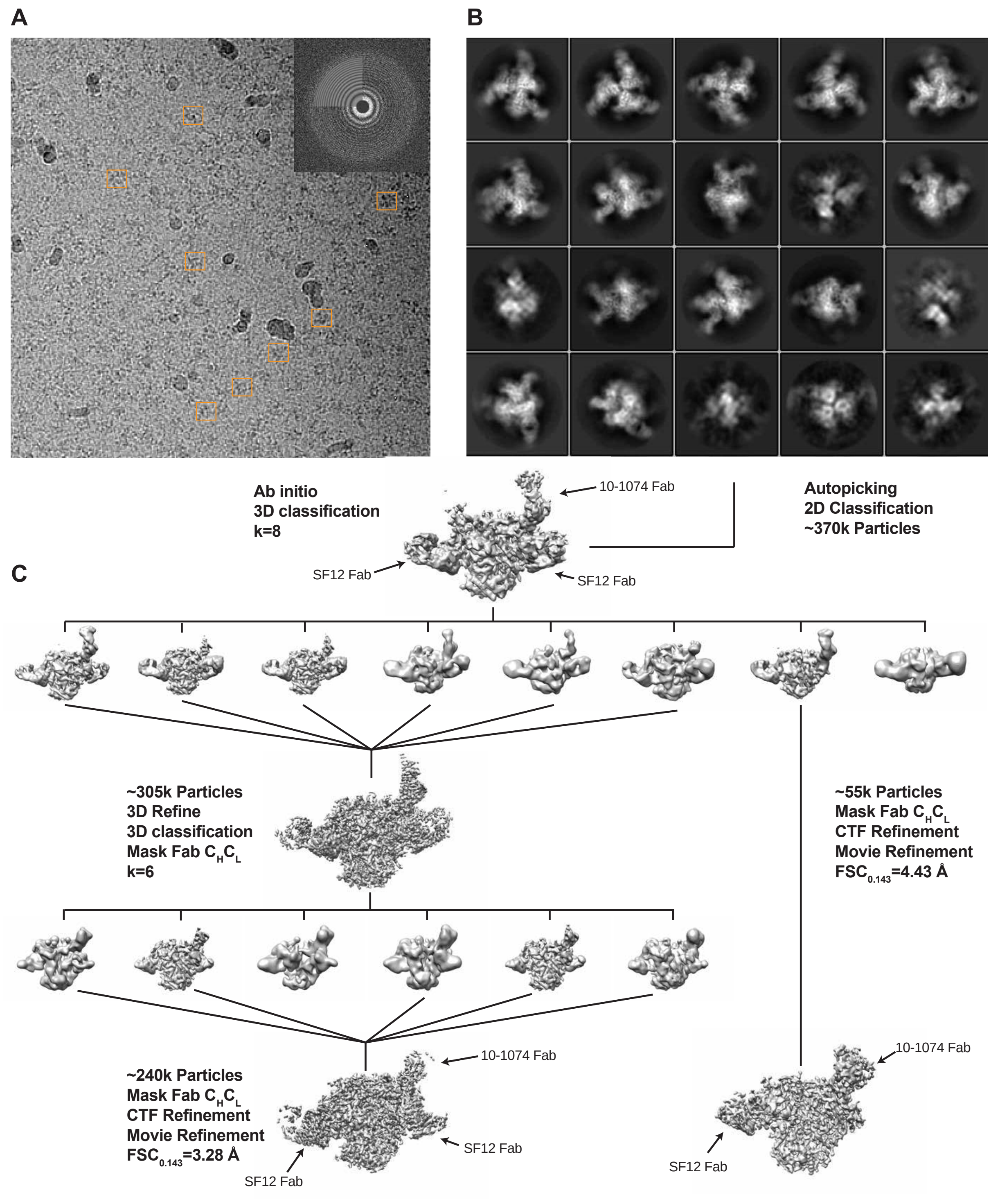

Figure S2. Data collection and processing for the SF12–B41–10-1074 complex. Related to Figure 3. (**A**) Representative micrograph of SF12-B41-10-1074 complex in vitreous ice with individual particles boxed (orange). Inset: power spectrum of micrograph determined during CTF estimation showing Thon rings to 3Å. (**B**) Reference-free 2D classification of 4x4 binned extracted particles. 2D class averages showing secondary structure were selected for further processing. (**C**) After 3D auto-refinement of ab initio model, good particles were subjected to rounds of 3D classification with C1 symmetry applied. 3D classes that showed similar features were pooled and 3D auto-refined to generate final reconstructions at 3.28Å and 4.36Å for class 1 and class 2, respectively.

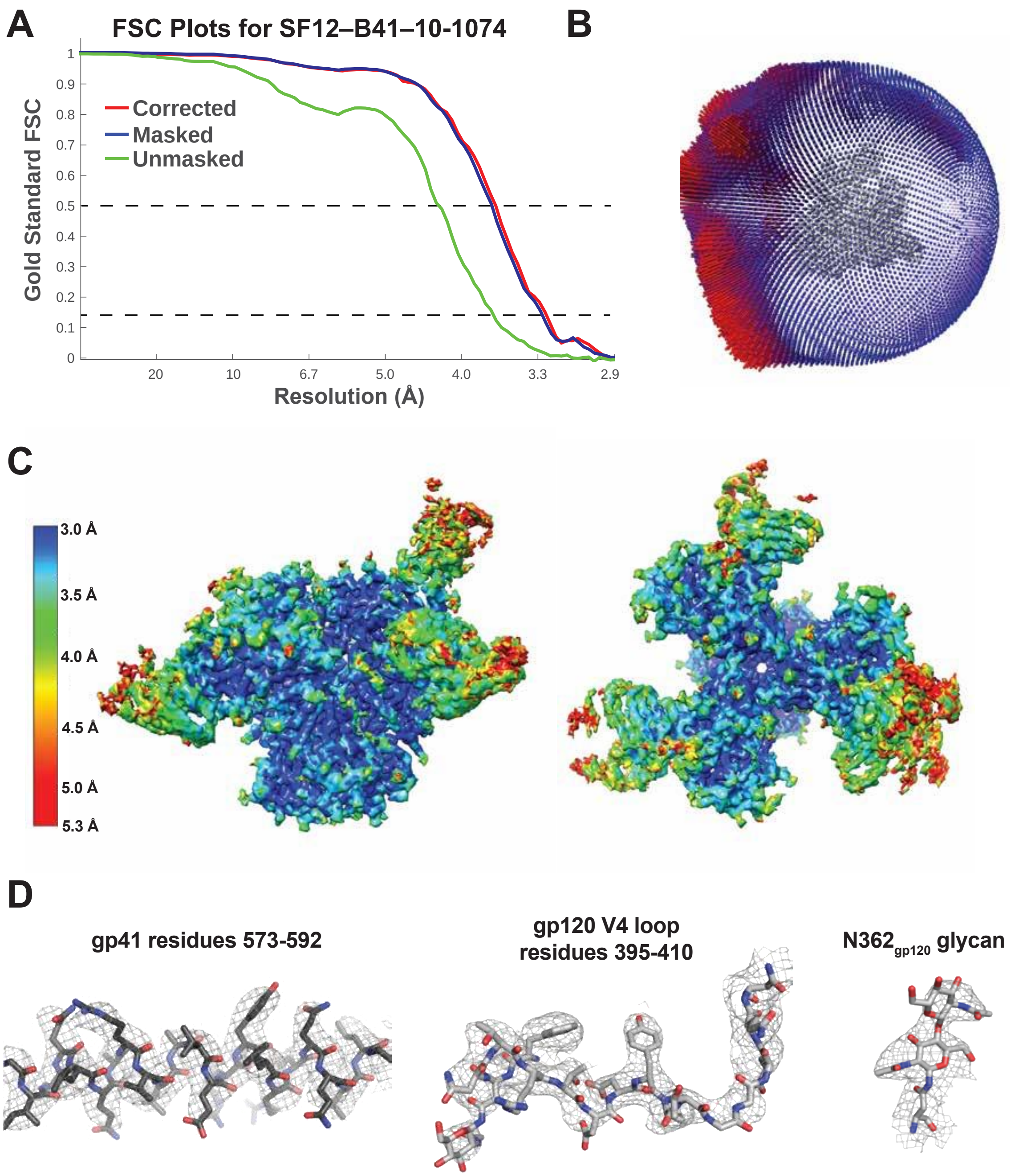

Figure S3. Validation and cryo-EM map quality. Related to Figure 3. (**A**) Fourier shell correlation (FSC) plots calculated from half-maps of masked (red), unmasked (blue), and corrected (black) data for Class 1. Dotted lines for FSC values of 0.5 and 0.143 are shown. (**B**) Angular distribution 3D histogram, (**C**) local resolution estimation (Resmap), and (**D**) representative density from Fab and Env regions of the Class 1 map.

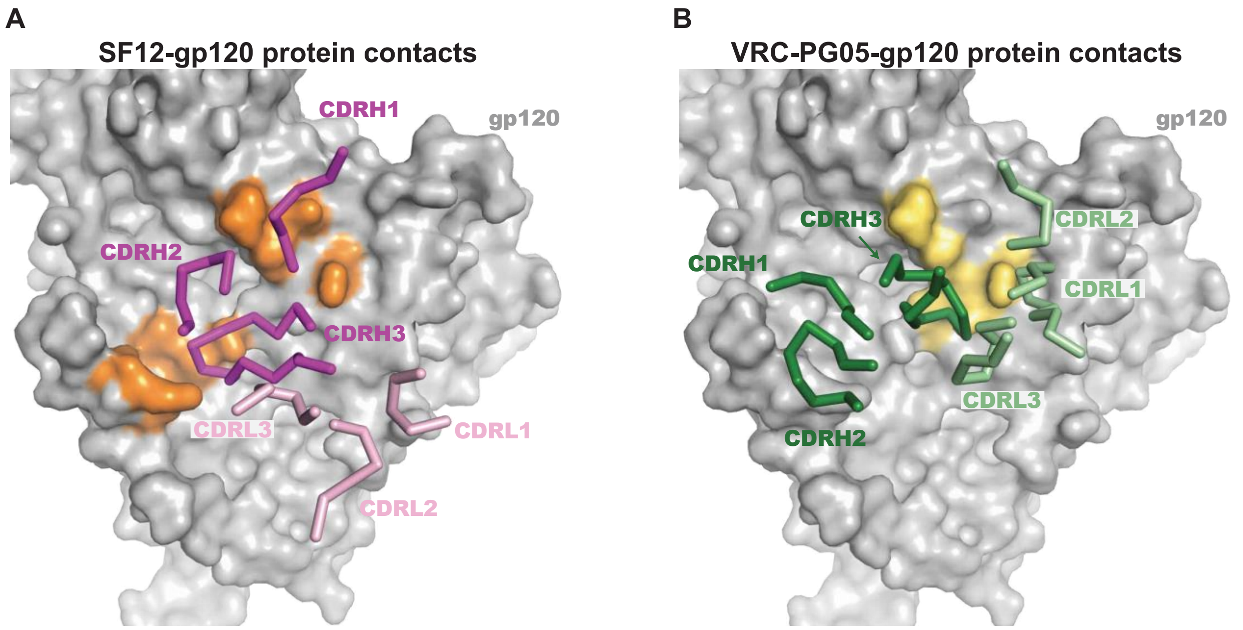

Figure S4. Comparison of SF12 and VRC-PG05 footprints on gp120. Related to Figure 3 and Figure 4. Differences in CDR loop orientations (ribbon) on gp120 (gray surface) by (**A**) SF12 (magenta, CDRH1-3; light pink, CDRL1-3) and (**B**) VRC-PG05 (forest green, CDRH1-3; light green, CDRL1-3). The protein epitope on the gp120 surface is highlighted in orange (SF12) or yellow (VRC-PG05). The glycan portion of the epitope was removed for clarity.

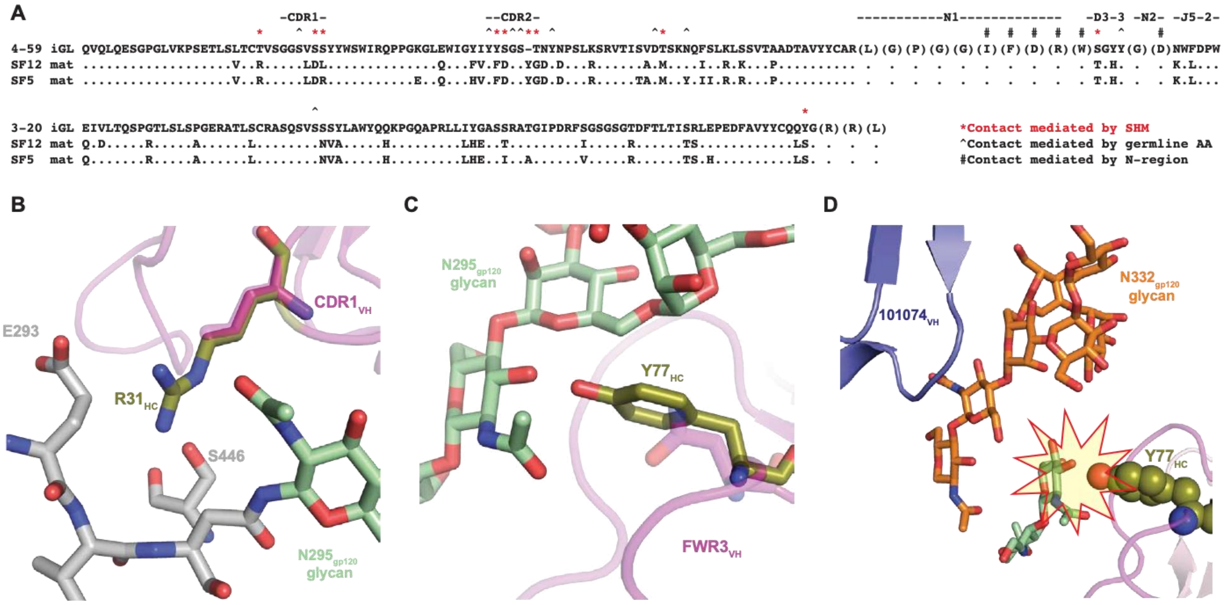

Figure S5. Comparison of SF12 and SF5 clonal variants. Related to Figure 2, Figure 5, and Figure 6. (**A**) Sequence alignment of SF12 and SF5 with deduced germline sequences. SHMs are shown for each antibody with paratope residues denoted. (**B, C**) Modeling of SF5 residues (olive) onto the SF12-Env structure. In each case, SHMs in SF5 potentially enhance recognition of the Env epitope through hydrogen bonding. (**D**) Modeling of SF5 $Y77_{HC}$ onto the 10-1074 bound protomer shows clashes between this residue and the slightly shifted $N295_{gp120}$ glycan (pale green).

Supplementary Tables

Table S1. Sequence analysis of silent face antibodies from donor 27845. Related to Figure 1.

	Antibodies cloned from microculture double hits									
	Heavy chain					Light chain				
MC Well	V-gene	% nt mutation	% AA mutation	CDRH3 (AA)	CDRH3 (seq)	V-gene	% nt mutation	% AA mutation	CDR3 (AA)	CDRL3 (seq)
SF3	IGVH4-59*01	25.3%	39.2%	23	GRVGPGGLFDRWRGYHGHKWVDA	IGKV3-20*01	21.4%	29.0%	6	QQYGRT
**SF5**	**IGVH4-59*01**	**19.0%**	**25.5%**	**23**	**ARLGPGGIFDRWTGHYGDKWLDP**	**IGKV3-20*01**	**16.0%**	**21.5%**	**6**	**QLSGRR**
SF7	IGVH4-59*01	25.3%	39.2%	23	GRVGPGGLFDRWRGYHGHKWVDA	IGKV3-20*01	21.4%	29.0%	6	QQYGRT
SF2	IGVH4-59*01	24.4%	32.0%	23	ARLGPGGLFDRYTGYHGRKWLDA	IGKV3-20*01	18.0%	23.9%	6	QQYGRT
SF8	IGVH4-59*01	24.4%	32.0%	23	ARLGPGGLFDRYTGYHGRKWLDA	IGKV3-20*01	16.5%	21.7%	6	QQYGRT
SF10	IGVH4-59*01	25.3%	37.1%	23	GRVGPGGLFDRWTGYHGHKWVDA	IGKV3-20*01	19.9%	29.0%	6	QQYGRT

	Clone members isolated by BG505-sorting upon microculture identification of clone									
	Heavy chain					Light chain				
Sort Well	V-gene	% nt mutation	% AA mutation	CDRH3 (AA)	CDRH3 (seq)	V-gene	% nt mutation	% AA mutation	CDR3 (AA)	CDRL3 (seq)
**SF12**	**IGVH4-59*01**	**17.0%**	**21.4%**	**23**	**ARLGPGGIFDRWTGHYGDKWLDP**	**IGKV3-20*01**	**14.6%**	**20.4%**	**6**	**QLSGRR**
SF10	IGVH4-59*01	25.3%	37.1%	23	GRVGPGGLFDRWTGYHGHKWVDA	IGKV3-20*01	19.9%	29.0%	6	QQYGRT

Table S2. 119 virus cross-clade panel neutralization data. Related to Figure 1.

Virus ID	Clade*	SF5			SF12		
		IC_{50}	IC_{80}	MPI	IC_{50}	IC_{80}	MPI
6535.3	B	0.13	0.48	100	0.11	0.58	100
QH0692.42	B	0.55	1.45	100	0.49	1.32	100
SC422661.8	B	0.19	0.64	100	0.20	0.69	100
PVO.4	B	0.08	0.31	100	0.07	0.35	100
TRO.11	B	0.44	2.32	99	0.35	1.40	100
AC10.0.29	B	0.03	0.08	100	0.01	0.06	100
RHPA4259.7	B	0.73	5.23	92	0.14	0.50	100
THRO4156.18	B	0.17	0.49	99	0.14	0.55	99
REJO4541.67	B	0.03	0.10	100	0.02	0.08	100
TRJO4551.58	B	0.63	2.18	99	0.33	0.92	100
WITO4160.33	B	>50	>50	14	0.75	15.37	88
CAAN5342.A2	B	0.04	0.11	100	0.12	0.25	100
WEAU_d15_410_787	B (T/F)	0.33	1.10	100	0.43	1.41	100
1006_11_C3_1601	B (T/F)	>50	>50	37	0.44	2.33	98
1054_07_TC4_1499	B (T/F)	0.12	0.82	100	0.21	1.58	100
1056_10_TA11_1826	B (T/F)	0.20	0.72	100	0.12	0.56	100
1012_11_TC21_3257	B (T/F)	>50	>50	17	0.23	1.11	100
6240_08_TA5_4622	B (T/F)	4.58	29.42	88	0.62	1.97	100
6244_13_B5_4576	B (T/F)	0.21	0.57	100	0.95	6.94	87
62357_14_D3_4589	B (T/F)	0.07	0.22	100	0.10	0.35	100
SC05_8C11_2344	B (T/F)	0.34	0.93	100	0.26	0.94	100
Du156.12	C	>50	>50	18	>25	>25	37
Du172.17	C	>50	>50	25	>25	>25	29
Du422.1	C	>50	>50	36	9.07	>25	73
ZM197M.PB7	C	0.19	0.67	99	10.48	>25	68
ZM214M.PL15	C	>50	>50	29	>25	>25	22
ZM233M.PB6	C	>50	>50	23	>25	>25	35
ZM249M.PL1	C	>50	>50	21	>25	>25	22
ZM53M.PB12	C	0.29	1.10	97	4.48	>25	75
ZM109F.PB4	C	0.10	0.22	100	0.24	0.88	99
ZM135M.PL10a	C	>50	>50	23	>25	>25	37
CAP45.2.00.G3	C	>50	>50	8	>25	>25	27
CAP210.2.00.E8	C	>50	>50	28	>25	>25	32
HIV-001428-2.42	C	0.04	0.11	100	0.05	0.20	100
HIV-0013095-2.11	C	>50	>50	13	>25	>25	21
HIV-16055-2.3	C	>50	>50	36	>25	>25	33
HIV-16845-2.22	C	>50	>50	35	4.10	>25	74
Ce1086_B2	C (T/F)	0.05	0.19	97	0.02	0.08	100
Ce0393_C3	C (T/F)	>50	>50	22	>25	>25	23
Ce1176_A3	C (T/F)	>50	>50	22	>25	>25	20
Ce2010_F5	C (T/F)	0.07	0.18	100	0.12	0.33	100
Ce0682_E4	C (T/F)	>50	>50	11	>25	>25	15
Ce1172_H1	C (T/F)	>50	>50	30	7.09	>25	76
Ce2060_G9	C (T/F)	26.93	>50	66	>25	>25	34
Ce703010054_2A2	C (T/F)	0.05	0.14	100	0.06	0.16	100
BF1266.431a	C (T/F)	7.51	>50	78	>25	>25	30
246F C1G	C (T/F)	>50	>50	14	>25	>25	14
249M B10	C (T/F)	>50	>50	1	>25	>25	6
ZM247v1(Rev-)	C (T/F)	>50	>50	39	>25	>25	25
7030102001E5(Rev-)	C (T/F)	>50	>50	23	>25	>25	21
1394C9G1(Rev-)	C (T/F)	2.25	18.61	87	2.03	23.15	81
Ce704809221_1B3	C (T/F)	34.15	>50	57	>25	>25	34
MS208.A1	A	>50	>50	22	>25	>25	23
Q23.17	A	0.27	7.04	90	0.11	0.68	95
Q461.e2	A	>50	>50	17	>25	>25	19
Q769.d22	A	0.27	>50	74	0.21	2.06	90
Q259.d2.17	A	1.18	>50	79	0.56	13.04	83
Q842.d12	A	0.07	0.26	100	0.07	0.25	100
0260.v5.c36	A	>50	>50	17	>25	>25	20
3415.v1.c1	A	>50	>50	10	>25	>25	14
3365.v2.c20	A	0.08	0.41	95	0.10	0.45	100
191955_A11	A (T/F)	0.58	3.01	95	0.16	0.52	100
191084 B7-19	A (T/F)	0.11	0.31	100	0.07	0.21	100
9004SS_A3_4	A (T/F)	6.51	>50	69	>25	>25	46

Virus ID	Clade*	SF5			SF12		
		IC_{50}	IC_{80}	MPI	IC_{50}	IC_{80}	MPI
CNE19	BC	>50	>50	31	>25	>25	38
CNE20	BC	>50	>50	25	>25	>25	39
CNE21	BC	>50	>50	25	>25	>25	28
CNE17	BC	0.09	0.31	100	0.11	0.38	100
CNE30	BC	>50	>50	13	>25	>25	12
CNE52	BC	>50	>50	8	>25	>25	8
CNE53	BC	46.80	>50	55	>25	>25	36
CNE58	BC	0.02	0.06	100	0.02	0.07	100
T257-31	CRF02_AG	>50	>50	11	>25	>25	4
928-28	CRF02_AG	>50	>50	15	>25	>25	10
263-8	CRF02_AG	3.00	17.90	89	4.19	24.51	80
T250-4	CRF02_AG	>50	>50	17	>25	>25	15
T251-18	CRF02_AG	>50	>50	49	12.13	>25	65
T278-50	CRF02_AG	>50	>50	29	>25	>25	50
T255-34	CRF02_AG	11.93	>50	62	>25	>25	34
211-9	CRF02_AG	16.28	>50	67	0.10	0.28	100
235-47	CRF02_AG	>50	>50	43	>25	>25	37
620345.c01	CRF01_AE	0.02	0.06	100	0.02	0.08	100
CNE8	CRF01_AE	0.05	0.18	99	0.12	0.59	97
C1080.c03	CRF01_AE	0.03	0.09	100	0.05	0.18	100
R2184.c04	CRF01_AE	0.05	0.14	100	7.43	>25	66
R1166.c01	CRF01_AE	0.22	0.59	97	0.61	2.59	93
R3265.c06	CRF01_AE	0.01	0.05	100	0.01	0.04	100
C2101.c01	CRF01_AE	0.03	0.09	100	0.04	0.19	100
C3347.c11	CRF01_AE	0.03	0.07	100	0.04	0.12	100
C4118.c09	CRF01_AE	0.06	0.20	98	0.10	0.45	98
CNE5	CRF01_AE	0.04	0.11	100	0.26	1.03	98
BJOX009000.02.4	CRF01_AE	0.02	0.06	100	0.02	0.07	100
BJOX015000.11.5	CRF01_AE (T/F)	0.02	0.10	100	0.03	0.15	100
BJOX010000.06.2	CRF01_AE (T/F)	>50	>50	18	11.20	>25	63
BJOX025000.01.1	CRF01_AE (T/F)	0.01	0.02	100	0.01	0.03	100
BJOX028000.10.3	CRF01_AE (T/F)	30.33	>50	57	0.04	0.18	98
X1193_c1	G	>50	>50	10	>25	>25	6
P0402_c2_11	G	0.03	0.08	100	0.03	0.07	100
X1254_c3	G	0.17	1.11	100	0.07	0.24	100
X2088_c9	G	>50	>50	12	>25	>25	9
X2131_C1_B5	G	>50	>50	21	>25	>25	25
P1981_C5_3	G	0.13	0.55	100	0.06	0.14	100
X1632_S2_B10	G	0.06	0.17	100	0.04	0.14	100
3016.v5.c45	D	0.04	0.12	100	0.10	0.33	100
A07412M1.vrc12	D	0.07	0.19	100	0.10	0.28	100
231965.c01	D	7.40	>50	70	0.26	0.57	100
231966.c02	D	0.17	0.59	99	0.12	0.42	100
6405.v4.c34	D	2.00	29.07	82	0.52	1.75	100
3817.v2.c59	CD	>50	>50	33	0.73	2.44	100
6480.v4.c25	CD	>50	>50	16	>25	>25	35
6952.v1.c20	CD	>50	>50	36	0.33	1.30	98
6811.v7.c18	CD	>50	>50	44	1.59	>25	76
89-F1_2_25	CD	>50	>50	15	>25	>25	19
3301.v1.c24	AC	>50	>50	21	>25	>25	22
6041.v3.c23	AC	0.20	0.76	96	0.43	1.31	97
6540.v4.c1	AC	7.07	>50	61	0.33	>25	70
6545.v4.c1	AC	5.04	>50	70	0.18	0.87	96
0815.v3.c3	ACD	>50	>50	21	>25	>25	45
3103.v3.c10	ACD	>50	>50	27	>25	>25	26
MuLV	Neg. Control	>50	>50	27	>25	>25	25

* (T/F): Transmitted / Founder Virus
MPI: Maximum Percent Inhibition

mAb titers (µg/ml)
- 0.001 - 0.01
- 0.01 - 0.1
- 0.1 - 1.0
- 1.0 - 20.0
- >20

Table S3. Cryo-EM data collection and refinement statistics. Related to Figure 3.

	**SF12 B41 SOSIP.664 v4.2 10-1074**
**PDB**	**6OKP**
**EMD**	**20100**
**Data collection and processing**	
Microscope	Titan Krios
Camera	Gatan K2 Summit
Magnification	130,000x
Voltage (kV)	300
Recording mode	counting
Dose rate (e^-/pixel/s)	4.8
Electron dose (e^-/Å^2)	40
Defocus range (µm)	1.2 - 3.0
Pixel size (Å)	1.09
Micrographs collected	2,732
Micrographs used	2,209
Total extracted particles	676,161
Refined particles	371,289
**Reconstruction**	
Final particles	301,920
Symmetry imposed	C1
Nominal Resolution (Å)	
FSC 0.5 (unmasked/masked)	4.15/3.67
FSC 0.143 (unmasked.masked)	3.71/3.28
Map sharpening *B*-factor	-110
**Refinement and Validation**	
Number of atoms	
Protein	20,391
Ligand	2,271
MapCC (global/local)	0.794/0.766
R.m.s. deviations	
Bond lengths (Å)	0.01
Bond angles (˚)	1.27
MolProbity score	1.78
Clashscore (all atom)	4.11
Poor rotamers (%)	0.48
Ramachandran plot	
Favored (%)	88.8
Allowed (%)	10.9
Disallowed (%)	0.31

Table S4. Crystallographic data collection and refinement statistics. Related to Figure 3.

PDB ID	**SF12 Fab** *(12-2, SSRL)* **6OKQ**
**Data collection**[a]	
Space group	$P6_222$
Unit cell (Å)	223, 223, 288
α, β, γ (°)	90, 90, 120
Wavelength (Å)	1.0
Resolution (Å)	39.28-3.2 (3.26-3.19)
Unique Reflections	70,865 (4,473)
Completeness (%)	99.6 (98.7)
Redundancy	60.4 (56.8)
$CC_{1/2}$ (%)	90.3 (86.4)
$\langle I/\sigma I \rangle$	22.8 (1.4)
Mosaicity (˚)	0.07
R_{merge} (%)	21.6 (255)
R_{pim} (%)	4.0 (57.8)
Wilson *B*-factor	81.6
**Refinement and Validation**	
Resolution (Å)	39.2-3.2
Number of atoms	
Protein	9,980
Ligand	0
R_{work}/R_{free} (%)	27.3/29.9
R.m.s. deviations	
Bond lengths (Å)	0.01
Bond angles (˚)	1.4
MolProbity score	2.86
Clashscore (all atom)	16.4
Poor rotamers (%)	6.6
Ramachandran plot	
Favored (%)	92
Allowed (%)	6.4
Disallowed (%)	0.54
Average *B*-factor (Å)	158.3

[a]Numbers in parentheses correspond to the highest resolution shell

Table S5. Buried surface area calculations at the SF12-Env interface. Related to Figure 4.

Components	Total Area (Å^2)	Heavy Chain	Light Chain	Percentage (%)
N262-glycan	542	542	0	27.2
N295-glycan	470	470	0	23.6
N448-glycan	545	392	153	27.3
Peptide	436	436	0	21.9
**Total**	**1993**			100.0

Table S6. Effects of site-specific mutations in HIV-1 gp120 on SF12 and SF5 neutralization. Related to Figure 5 and 6.

	Titer in TZM.bl cells (ug/ml)									
	SF12		SF5		3BNC117		10-1074		PGDM1400	
Virus ID	IC_{50}	IC_{80}	IC_{50}	IC_{80}	IC_{50}	IC_{80}	IC_{50}	IC_{80}	IC_{50}	IC_{80}
BG505 WT	0.11	0.30	0.14	0.62	0.04	0.13	0.07	0.22	<0.01	0.03
BG505 WT	0.13	0.40	0.17	0.76	0.05	0.15	0.07	0.22	<0.01	0.03
BG505 P214Q	0.24	0.83	>25	>25	0.06	0.20	0.08	0.26	<0.01	0.03
BG505 P214I	0.27	0.87	>25	>25	0.05	0.19	0.07	0.24	<0.01	0.03
BG505 N262S	>25	>25	>25	>25	0.02	0.09	0.05	0.15	<0.01	0.02
BG505 N262W	>25	>25	>25	>25	0.03	0.27	0.10	0.28	<0.01	0.08
BG505 Q293E	0.25	0.86	0.17	0.57	0.03	0.14	0.09	0.29	<0.01	0.03
BG505 Q293K	0.06	0.21	2.23	>25	0.04	0.15	0.08	0.25	<0.01	0.03
BG505 Q293R	0.06	0.18	0.30	6.24	0.05	0.16	0.06	0.23	<0.01	0.03
BG505 N295T	0.03	0.10	0.04	0.18	0.04	0.13	0.03	0.11	<0.01	0.03
BG505 N295V	0.04	0.12	0.07	0.44	0.04	0.15	0.04	0.13	<0.01	0.03
BG505 R444T	0.27	0.90	>25	>25	0.04	0.13	0.08	0.25	<0.01	0.03
BG505 N448K	>25	>25	>25	>25	0.05	0.17	0.06	0.21	<0.01	0.03
BG505 N448S	>25	>25	>25	>25	0.04	0.12	0.06	0.19	<0.01	0.02

	Titer in TZM.bl cells (ug/ml)									
	SF12		SF5		3BNC117		10-1074		PGDM1400	
Virus ID	IC_{50}	IC_{80}	IC_{50}	IC_{80}	IC_{50}	IC_{80}	IC_{50}	IC_{80}	IC_{50}	IC_{80}
YU2 WT	0.52	1.64	0.70	2.29	<0.01	0.06	0.15	0.58	0.27	0.89
YU2 WT	0.61	1.81	0.89	2.67	0.01	0.09	0.17	0.59	0.23	0.86
YU2 P214Q	0.44	1.52	8.72	>25	<0.01	0.07	0.07	0.32	0.21	0.85
YU2 P214I	0.23	0.85	6.04	>25	<0.01	0.03	0.02	0.15	0.12	0.65
YU2 N262S	>25	>25	>25	>25	<0.01	0.02	<0.01	0.08	0.15	0.60
YU2 N262W	>25	>25	>25	>25	<0.01	<0.01	<0.01	0.09	0.19	1.47
YU2 S291P	0.58	1.72	0.71	2.15	0.02	0.07	0.14	0.50	0.31	1.13
YU2 S291T	0.54	1.65	0.86	2.30	0.02	0.08	0.18	0.60	0.30	0.98
YU2 V293E	0.86	2.46	0.62	2.02	0.01	0.06	0.11	0.42	0.28	1.09
YU2 V293K	0.32	0.90	4.73	>25	0.01	0.06	0.13	0.52	0.24	0.87
YU2 V293R	0.22	0.71	4.83	>25	<0.01	0.05	0.12	0.50	0.24	0.92
YU2 N295T	0.07	0.24	0.06	0.20	0.02	0.08	0.07	0.28	0.27	1.01
YU2 N295V	0.04	0.20	0.03	0.17	0.01	0.06	0.05	0.32	0.24	0.79
YU2 R444T	1.06	3.36	3.13	16.23	0.01	0.06	0.11	0.51	0.22	0.91
YU2 N448K	>25	>25	>25	>25	0.01	0.08	0.16	0.56	0.32	1.15
YU2 N448S	>25	>25	>25	>25	<0.01	0.07	0.15	0.55	0.26	1.14

Table S7. Computational analysis of neutralization data for silent face antibodies. Related to Figure 5 and 6.

	SF12		SF5		VRC-PG05	
	IC_{50} (µg/ml)[a,b]	Coverage (%)	IC_{50} (µg/ml)	Coverage (%)	IC_{50} (µg/ml)	Coverage (%)
**Viruses**						
Cross-clade panel	0.20 (n=119)	62.2%	0.25 (n=119)	58.0%	0.84 (n=220)	27.3%
Clade AE	0.09 (n=15)	100.0%	0.05 (n=15)	93.3%	1.56 (n=35)	57.1%
Clade B	0.19 (n=21)	100.0%	0.2 (n=21)	85.7%	0.79 (n=41)	31.7%
Clade C	0.69 (n=31)	35.5%	0.49 (n=31)	33.3%	0.55 (n=71)	22.5%
**Glycans**						
N448+	0.21 (n=98)	75.5%	0.23 (n=98)	69.4%	0.81 (n=168)	32.1%
N448-	>50 (21)	0.0%	>50 (21)	0.0%	>50 (24)	0.0%
N295+	0.19 (n=56)	73.2%	0.29 (n=56)	64.3%	2.20 (n=94)	26.6%
N295-	0.25 (n=63)	51.6%	0.21 (n=63)	51.6%	0.34 (n=98)	29.6%
N442+	0.29 (n=41)	41.5%	0.3 (n=41)	39.0%	0.84 (n=60)	21.7%
N442-	0.19 (n=78)	72.4%	0.24 (n=78)	67.5%	0.8 (n=132)	31.1%
**Peptide**						
E293+	0.2 (n=54)	64.8%	0.16 (n=54)	63.3%	0.82 (n=95)	51.6%
E293-	0.22 (n=65)	59.4%	0.38 (n=65)	53.8%	0.66 (n=97)	5.2%
S291+	0.2 (n=74)	67.6%	0.2 (n=74)	64.9%	0.7 (n=118)	39.0%
S291-	0.25 (n=45)	52.3%	0.43 (n=45)	45.5%	1.9 (n=74)	10.8%
T444+	0.38 (n=46)	37.0%	0.29 (n=46)	34.8%	0.35 (n=69)	21.7%
T444-	0.18 (n=73)	77.8%	0.24 (n=73)	72.2%	1.11 (n=123)	31.7%

[a]Mean IC_{50} of neutralized strains

[b]Number in paranthesis represents number of strains that fit the filtered criteria
